# Epithelial cell extrusion underlies starvation-induced cell loss in a sea anemone

**DOI:** 10.1038/s41467-026-74437-7

**Published:** 2026-06-17

**Authors:** Inés Fournon-Berodia, Noah Bruderer, Lionel Christiaen, Patrick R. H. Steinmetz

**Affiliations:** Michael Sars Centre, University of Bergen, Bergen, Norway

**Keywords:** Evolutionary developmental biology, Cellular imaging, Cell death, Self-renewal

## Abstract

Epithelia likely predate the last common animal ancestor, yet the evolutionary origin and nutritional regulation of epithelial remodelling remain poorly understood. Here, we show that extensive, starvation-induced cell loss in the sea anemone *Nematostella vectensis* is associated with epidermal cell extrusion. This process involves formation of a rosette-like arrangement in which an apoptotic, extruding cell is surrounded by a phospho-ERK1/2-positive ring of cells, accompanied by basal translocation of adherens junction components. Combining chemical perturbations with computational quantification of extrusion and cell density, we show that apoptosis is necessary but not sufficient for rosette formation, and that ERK1/2 signalling limits epidermal extrusion density. Furthermore, we find increased extrusion activity during starvation, and potential nutrient recycling via phagocytosis of extruded cells. Together, our findings indicate that epithelial cell extrusion has physiological roles in sea anemones and that its key hallmarks are likely evolutionarily ancient, predating the last common cnidarian-bilaterian ancestor.

## Abstract (web summary)

Epithelial cell extrusion in a sea anemone shares key hallmarks with bilaterians and is dynamically regulated by nutritional status. During starvation-induced body shrinkage, extrusion likely contributes to cell loss and nutrient recycling.

## Introduction

**Fig. 1:**
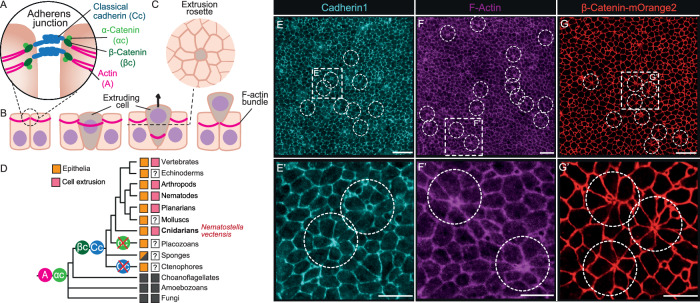
Identification of rosette-like cell arrangements in the epidermis of juvenile *Nematostella vectensis*. **A**–**C** Schematic summary of bilaterian epithelial cell extrusion process, focussing on the composition of adherens junction (AJ) components **B** and the shape of a rosette cell arrangement (**C**). From left to right: cell extrusion was found to be driven by the constriction of F-actin (magenta) rings in the extruding cell and adjacent cells, leading to detachment of the extruding cell from the basement membrane. **D** Simplified phylogenetic tree highlighting the phylogenetic occurrence of epithelia, cell extrusion and key AJ components, and the position of Cnidaria (incl. *Nematostella vectensis*) as sister group to Bilateria. Crossed circles indicate secondary losses within taxon. Grey boxes represent full (full grey) or partial (half grey) character absence within taxon. **E**–**G’** Projections of confocal microscopy images show rosette-like structures in the epidermis as highlighted by fixed epidermal tissue labelling using an antibody detecting *Nematostella* Cadherin-1 (**E**, **E’**), Phalloidin to detect F-actin (**F**, **F’**), or by live imaging of epidermis labelled by a β-Catenin-mOrange2 fusion protein in transgenic reporter polyps (**G**, **G’**). Scale bars: 10 µm (**E**–**G**), 20 µm (**E’**–**G’**). Each experiment in **E**–**G’** was performed independently 3 or more times with similar results.

Epithelia are a key evolutionary novelty of animals found throughout metazoans, including sponges, where their existence has long been debated^1,2^. They form a protective barrier and allow the development of structurally distinct tissues and organs. Key molecular components of epithelia, such as the Catenin/Cadherin complex at adherens junctions (AJ) (Fig. 1A, B) or the Integrin-based cell-matrix junction components, are widely conserved across nearly all major metazoan groups (Fig. 1D)^3^. Together, these findings led to the commonly accepted hypothesis that epithelia predate the last common metazoan ancestor^4^. Epithelia undergo a constant cell turnover that is essential for their renewal and integrity, and for responding to environmental insults, such as wounding. An imbalance in this turnover can have dramatic effects leading to over-proliferation or reduced cell loss, which are prime causes of many cancer types^5,6^. Epithelial homeostasis is therefore important and achieved by tightly balancing cell proliferation and cell loss, which occurs by apoptosis and epithelial cell extrusion.

Over the last decade, studies in mammals^7^, zebrafish^8^, nematodes^9^ and flies^10^ have uncovered epithelial cell extrusion as a tightly regulated process to remove single epithelial cells without compromising the structural integrity of the epithelium. So far, it has been mainly studied in the context of development, during morphogenesis and as response to mechanical tissue stress, but little is known about its regulation by metabolic factors such as nutrient availability. In vertebrates and flies, cells surrounding the extruding cell adopt a characteristic ‘rosette’ formation, accompanied by actomyosin and AJ remodelling in both extruding and rosette cells (Fig. 1A–C). While rosette formation is a hallmark of epithelial cell extrusion, it can also occur during morphogenetic cell rearrangements^11^. During extrusion, the central cell typically undergoes Caspase-mediated apoptosis. However, it remains unclear whether apoptosis triggers extrusion or occurs as a secondary consequence, for example through anoikis, where apoptosis is initiated by loss of basal membrane contact^12^. Contraction of the supracellular ring-like actomyosin system in rosette and central cells leads to apical, basal or lateral extrusion^13,14^. A hallmark of extrusion in flies and vertebrates is the activity of extracellular signal-regulated kinases (ERK) pathway signalling in single or multiple rows of rosette cells, depending on the species and tissues^15–17^. ERK signalling is currently thought to mediate extrusion in a non-cell autonomous fashion and protect the surrounding epithelium against pro-apoptotic signals from the extruding cell^15,16^. A major cause of cell extrusion is epithelial crowding, but how cells are selected for extrusion remains poorly understood^10,18,19^.

In many bilaterian animals, nutrient availability greatly affects tissue homeostasis, as shown by starvation-induced atrophy of the intestinal epithelium in mice^20^, snakes^21^, and insects^22,23^. In extreme cases, starvation leads to whole-body shrinkage that is rare among vertebrates (e.g., fish, amphibians, reptiles)^24–26^, but common among planarians^27^, acoels^28^ and non-bilaterian taxa, including sea anemones^29,30^ (Fig. 1D). However, the environmental control of body and tissue plasticity remains poorly understood on cellular and molecular levels. In *Drosophila*, starvation induces shrinkage of the midgut epithelium by apoptosis- and cell extrusion-mediated cell loss^23,31^. Similarly, starvation-induced shrinkage in planarians is mediated by basal cell extrusion in the epidermis, although the underlying cellular and molecular mechanisms remain unclear^32,33^.

Recently, the sea anemone *Nematostella vectensis* has emerged as a powerful model system for studying the molecular and cellular basis of body plasticity. As is typical for cnidarians, *Nematostella* is composed of only two epithelial cell layers: the outer epidermis and inner gastrodermis^34^. *Nematostella* juveniles survive months of starvation and lose >50% of their total cell count over three weeks, starting as soon as 2 days after the last meal^30,35^. Such extensive cell loss necessitates large-scale epithelial rearrangement, making this system uniquely suited to study how environmental cues regulate epithelial remodelling. Here, we show that nutritional fluctuations trigger cell extrusion in *Nematostella*, revealing an ancient epithelial cell elimination mechanism that likely predates the cnidarian–bilaterian split.

## Results

### Cell extrusion rosettes in the *Nematostella* epidermis

**Fig. 2:**
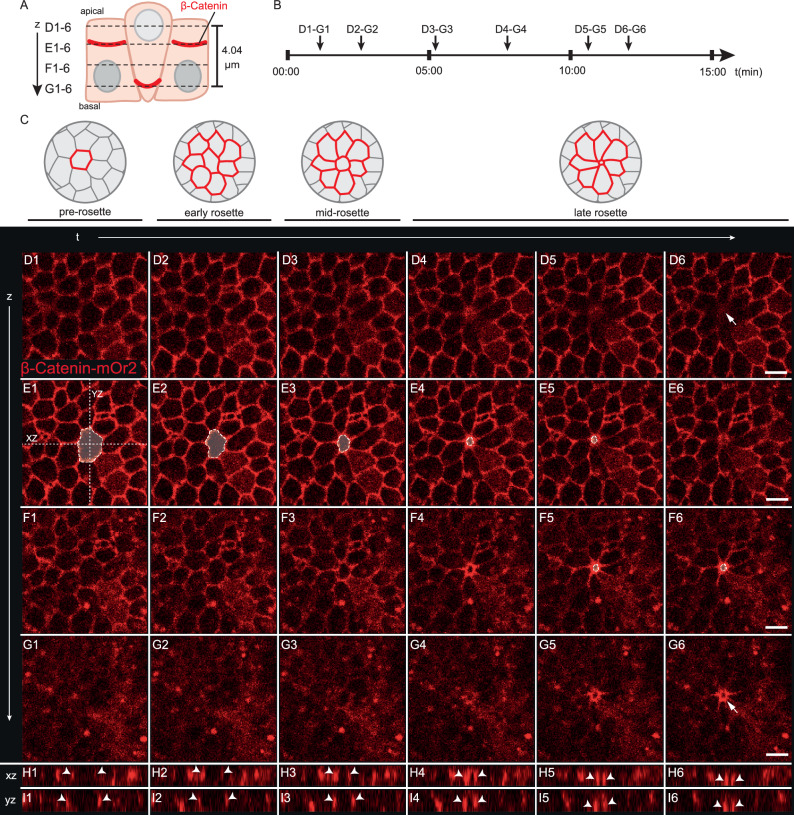
Live imaging of β-catenin-mOrange2 revealed the presence of epidermal cell extrusion in juvenile *Nematostella* polyps. Schematics indicating the stack height and relative *z*-axis positions (**A**), and the timestamps **B** corresponding to images in (D1-I6). **C** Schematics representing the four stages of rosette formation. (D1-I6) Single plane in vivo confocal microscopy images of β-Catenin-mOrange2, depicting the formation of an epithelial rosette from pre-rosette stage (D1-I1) through progressive rosette formation, constriction and extrusion of the central cell (shaded in E1-E5, F5-F6). XZ (H1-H6) and YZ (I1-I6) views were reconstructed along the planes depicted in E1 (dotted lines). Note the basal translocation of the central ring (arrows and arrowheads). See also Supplementary Movie 1 and Supplementary Data 1. Scale bars: 5 µm. In addition to the cell extrusion event depicted in D1-I6, we found 12 additional, independent cases of similar events (see also Supplementary Fig. 3).

We aimed to monitor epithelial cell extrusion in *Nematostella* *vectensis* (*Nv*) by searching for rosette-like cell arrangements. We visualised cell outlines in the epidermis of the body column by immunolabelling three AJ components: NvCadherin1^36^ (Fig. 1E, E’), F-actin (Fig. 1F, F’) and a β-Catenin-mOrange2 fusion protein in a newly generated transgenic knock-in line (Fig. 1G, G’ and Supplementary Fig. 1B). Throughout this study, we focused on the body column epidermis of juvenile polyps, which is particularly accessible to in vivo confocal microscopy (Supplementary Fig. 1B, C). In all three labelling assays, we observed rosette-like cell arrangements in the *Nematostella* epidermis (Fig. 1E–G’). Because rosettes can also occur during morphogenetic cell rearrangements^11^, we established in vivo confocal time-lapse imaging for the *Nematostella* juvenile epidermis to visualise and characterise cell extrusion events (Fig. 2 and Supplementary Fig. 1B). These recordings confirmed progressive constriction and eventual disappearance of the central cell, accompanied by a shift of the intracellular β-Catenin-mOrange2 and F-actin from the apical cortex towards the basal side of the cell (Fig. 2D1–I6 and Supplementary Fig. 2C1–G3). By contrast, the transmembrane AJ component NvCadherin1 remained more apically distributed along the basolateral membrane, without specific enrichment from the β-Catenin-mOrange2-/F-actin-rich basal ring structure (Supplementary Fig. 2J1–N3).

Based on these observations, we propose four stages of epithelial extrusion in the *Nematostella* epidermis: (i) a ‘pre-rosette’ stage (Fig. 2D1–I1), in which the future extruding cell is indistinguishable from its neighbours; (ii) an ‘early rosette’ stage (Fig. 2D2–I2 and Supplementary Fig. 2C1–G1), marked by the initiation of rosette formation and central cell constriction; (iii) a ‘mid-rosette’ stage (Fig. 2D3–I3 and Supplementary Fig. 2C2–G2), during which β-Catenin and F-actin become apparent basally within a clearly defined rosette; and (iv) a ‘late rosette’ stage (Fig. 2D4–I6 and Supplementary Fig. 2C3–G3), characterised by a basal ring or dot of β-Catenin or F-actin and culminating in extrusion. In vivo, the entire process took ~12 min (11:33 ± 2:41 min, *n* = 13 extrusion events) from the early rosette stage initiation to complete extrusion (Supplementary Fig. 3B), without clear differences in duration between fed and starved animals (Supplementary Fig. 3B, Supplementary Data 1). This is much shorter than the hours-long extrusion process reported for the *Drosophila* gut epithelium^31^ and similar in duration to extrusion in the zebrafish periderm^37^ and in the *Drosophila* pupal notum, leg, and abdominal epithelia^38–41^.

To get insights into the direction of cell extrusion, we studied the location of nuclei within the epithelium. Live imaging of β-Catenin-mOrange2 combined with nuclear staining revealed cases of apical cell extrusion, where the nucleus of an extruding cell appeared apically in the plane of the adherens junction (Supplementary Fig. 3A–A” and Supplementary Movie 2). The pronounced nuclear DNA stain is consistent with genomic DNA condensation, one of several hallmarks of cell death (Supplementary Fig. 3C–C”). We also found cases where highly fragmented nuclei, indicative of late apoptosis, were located near the basal side of rosette cells (Supplementary Fig. 3D–E’). These may have resulted from basal or lateral extrusion events followed by phagocytosis into rosette cells (see also later Results sections). Our observations indicate that epithelial cell extrusion in the *Nematostella* juvenile epidermis occurs primarily apically, but occasional basal or lateral extrusion events remain possible.

### Rosette and cell density dynamics in fed and starved polyps

**Fig. 3:**
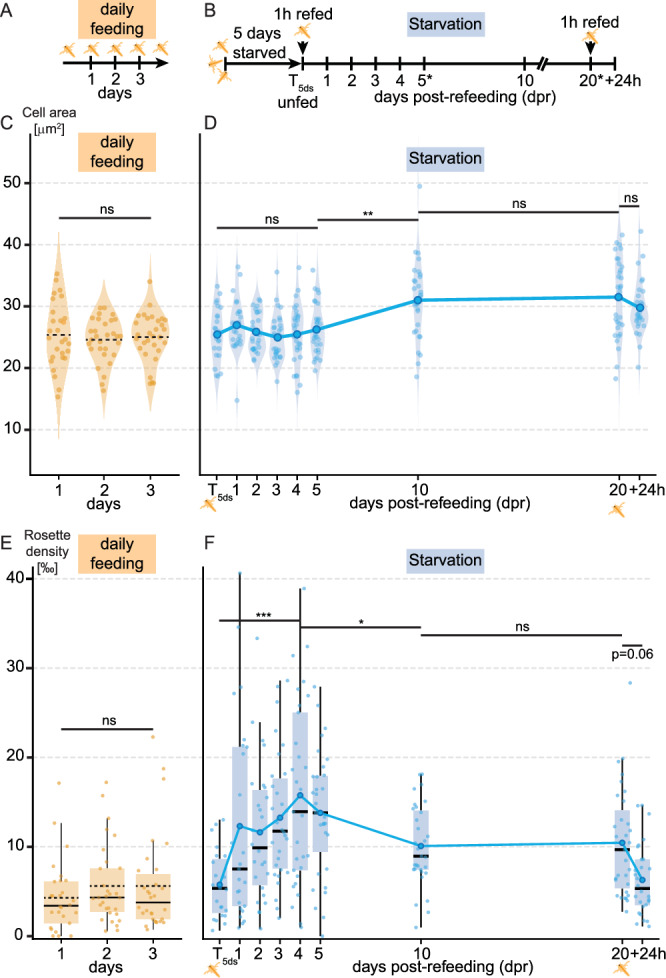
Following rosette density and cell area changes during daily feeding and starvation. **A**, **B** Sampling strategy. Labelled vertical stick-marks represent sampled timepoints. **A** Animals were fed daily for 5 h and sampled over 3 consecutive days. **B** Polyps sampled at T_5ds_ were starved for 5 days following continuous daily feeding. At T_5ds_, animals were refed for 1 h and then sampled as depicted between 1 and 20 days post-refeeding (dpr). At 20dpr, animals were refed for 1 h and sampled 24 h later (+24 h). Asterisks indicate stages used for inhibitor studies in Figs. 4 and 5 and Supplementary Figs. 8 and 9. Violin plots show cell area distribution of large cells. Means per timepoint are represented as black dashed lines (**C**) or a central circle (**D**) joined to form a trend line. **C** Mean cell area of large cells was not significantly different across 3 days of daily feeding. **D** Mean cell area of large cells remained stable between T_5ds_ and 5 dpr, significantly increased between 5 and 10 dpr, and remains stable between 10 and 20 dpr. The decrease in mean cell area upon refeeding (+24 h) was not significant. Box plots depicting rosette density, the number of rosettes identified per 1000 large cells. Means are shown as a black dashed line (**E**) or as central circles, joined to form a trend line (**F**). Median values are shown as black full lines (**E**, **F**). Box plots show the first to third interquartile ranges (box); whiskers indicate 1.5x IQR. **E** Rosette density was not significantly different across 3 days of daily feeding. **D** Rosette density increased by ~2.5-fold between *T*_5ds_ and 4 dpr, decreased to intermediate levels between 4 dpr and 10 dpr and stayed stable between 10 dpr and 20 dpr. The decline in rosette density after refeeding at +24 h was close to statistical significance. **C**–**F** Two-sided linear mixed-effects models with Tukey-adjusted EMM pairwise comparisons were used for data analysis. Adjusted *P*-values significance codes: *:*P* < 0.05, **:*P* < 0.01, ***:*P* < 0.001; ns non-significant. Single data points represent mean cell area of large cells (**C**, **D**) or mean rosette density (**E**, **F**) per animal. Abbreviations: h hour, ds days starved. See Supplementary Data 2–6.

Our findings suggest that epidermal cell extrusions contribute to the substantial cell loss reported during starvation-induced body shrinkage^30^. We therefore set out to characterise the dynamics of extrusion during feeding and starvation. To quantify epidermal rosettes in a reproducible, high-throughput manner, we developed an image segmentation and rosette prediction pipeline to analyse large numbers of maximum intensity projection (MIP) in vivo confocal 2D images of the β-catenin-mOrange2-labelled epidermis in transgenic juvenile polyps (Supplementary Fig. 4; Methods and Supplementary Notes). Using these images, we segmented individual epidermal cells with a fine-tuned Cellpose model to automatically predict cell outlines^42^. This semi-automated approach accelerated quantification by ~5-fold compared with manual rosette identification (Supplementary Fig. 4, Methods and Supplementary Notes). It enabled unbiased identification and quantification of cell outlines, cell area and rosettes density across 684 images over both timeseries datasets, during daily feeding (Fig. 3A) and between 1 and 20 days of starvation (Fig. 3B).

In *Nematostella* juveniles, cell proliferation rates are strongly feeding-dependent^30,35^. Strict control of feeding history is therefore required to control proliferation and, consequently, cell density, which influences cell extrusion rates in both vertebrates and flies^10,43^. We therefore sampled polyps either during daily feeding or following a standardised starvation/refeeding protocol consisting of a 1-hour refeeding pulse after 5 days of prior starvation (T_5ds_; sampled before refeeding) (Fig. 3A, B). This induces a controlled and relatively synchronised proliferative response in >50% of all cells within 48 h, before returning to baseline during continued starvation^35^. Notably, a 1-h refeeding pulse is sufficient to elicit a full proliferative response, comparable to 1 day of *ad libitum* refeeding^35^. This protocol thus ensures controlled feeding history across animals and precise control over the onset and duration of starvation.

Using cell segmentation masks, we quantified epidermal cell area as a proxy for tissue density. We found a bimodal distribution of cell area measurements into two populations (Supplementary Fig. 5A): (i) ~25% small cells of ~6.3 µm^2^ (95% CI = 6.2–6.4)), likely including stinging cells (i.e., cnidocytes) and sensory neurons; and (ii) ~75% larger cells measuring ~27 µm^2^ (95% CI = 26.2–27.9), likely consisting predominantly of epidermal cells (Supplementary Fig. 5B, C). The population of small cells showed no significant changes in mean cell area after feeding or during starvation, consistently remaining within a narrow range (6.1–6.8 µm²) (Supplementary Fig. 5D–G). We thus focussed our analysis on changes in the population of larger epidermal cells (Fig. 3C, D). Within this population, mean cell areas did not significantly differ between any time points over three days of daily feeding (Fig. 3C), between T_5ds_ and 5 days post-refeeding (dpr) or between 10 and 20 dpr (Fig. 3D). However, we found a significant ~20% increase in mean cell area between 5 and 10 dpr (Tukey-adjusted *p* < 0.001), from ~26 µm² (95% CI: 24.4–27.6) to ~30 µm² (95% CI: 28.6–32.4) (Fig. 3D). Notably, cell areas at 10 and 20 dpr (~31 µm², 95% CI: 29.4–34.0) are also significantly higher (~27% adjusted *p*-value < 0.001) compared to daily fed samples (~25 µm², 95% CI: 23.3–26.8). Together, this data shows that cell density is similar between daily fed, *T*_5ds_ and over the first 5 days after refeeding at *T*_5ds_, but that the epidermis becomes significantly less dense during prolonged starvation.

Across all timeseries datasets (Fig. 3A, B), epidermal rosettes typically comprised an average of 6.0 ± 1.8 cells, which is nearly identical to the number reported in the mammalian gut (~6 cells)^44^. We then quantified epithelial rosette density, corresponding to the number of rosettes per 1000 large cells in the body column epidermis. In daily fed polyps, we detected ~5‰ epidermal rosettes (Fig. 3E; 95% CI: 3.4–6.9). Refeeding at *T*_5ds_ led to a significant, ~2.5-fold increase in mean rosette density from ~6‰ at *T*_5ds_ (sampled before refeeding; 95% CI: 1.3–10.6) to ~15‰ at 4 days post-refeeding (dpr) (95% CI: 10.2–19.9; Tukey-adjusted *p* < 0.001) (Fig. 3F). Between 4 and 10 dpr, mean rosette density decreased significantly by ~30% to intermediate levels of ~10‰ (95% CI: 5.2–15.2; Tukey-adjusted *p* = 0.033) (Fig. 3F). Notably, rosette density at 10 and 20 dpr (~10‰; 95% CI: 8.1–13.3) remained ~2-fold higher compared to daily fed animals (~5‰, 95% CI: 2.6–8.2) or to T_5ds_ (~6‰; 95% CI: 1.3–10.6) (Fig. 3E, F and Supplementary Data 4). Refeeding for 1 h after 20 days of starvation led to a ~40% decline in mean rosette density to ~6‰ within 24 h, reflecting the approximate levels in daily fed polyps or at T_5ds_ (Fig. 3F) (Tukey-adjusted *p* = 0.0592, 95% CI: 1.3–11.5). This suggests that refeeding at 20 dpr had the opposite effect on extrusion dynamics compared to refeeding at *T*_5ds_ (Fig. 3F). In summary, our results reveal dynamic changes in extrusion activity across feeding and starvation conditions that are not solely determined by tissue density.

### ERK1/2 signalling regulates rosette density

**Fig. 4:**
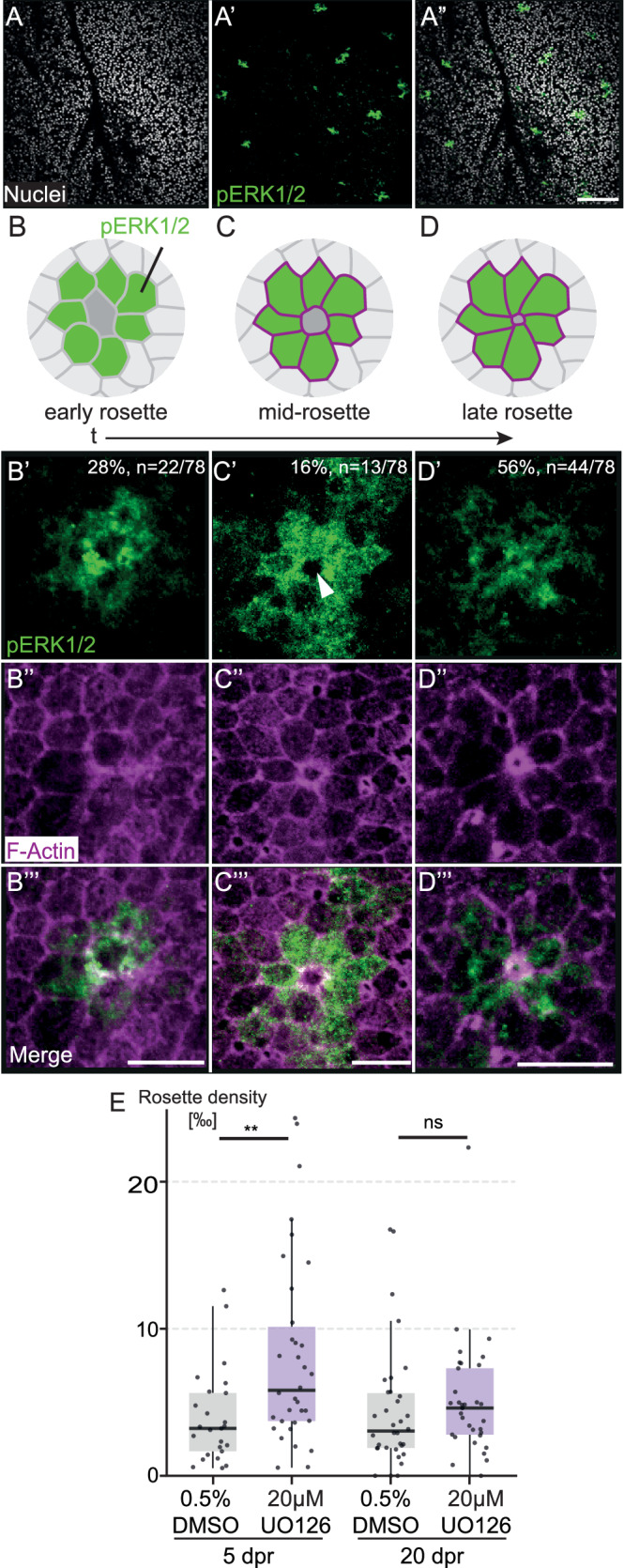
ERK1/2 signalling is activated in rosettes, and its inhibition increases rosette density. **A**–**A**” Projections of confocal microscopy images showing scattered cell patches immunolabelled by phospho-ERK1/2 (pERK1/2) in the fixed juvenile *Nematostella* epidermis. Schematics (**B**–**D**) summarising the results of Z-projections of confocal microscopy images depicting co-localisation of pERK1/2 label (**B**’, **B**”’, **C**’, **C**”’ **D**’, **D**”’) with F-actin-labelled rosettes (**B”**, **B”’**, **C”**, **C”’**, **D”**, **D”’**). The experiments shown in **A**–**A**” and **B**’–**D**”’ were performed independently three or more times with similar results. *n* = number of individual F-actin rosettes identified across images from more than three independent experiments (see Supplementary Data 1). **E** Box plots show mean rosette density (i.e., rosette number per 1000 large cells) in the epidermis of polyps at 5 or 20 days post-refeeding treated for 2 h in the MEK inhibitor U0126 or in 0.5% DMSO as control. Inhibition of MEK, the kinase directly upstream of ERK1/2, led to an increase in rosette density that was only significant at 5dpr. Normality was assessed using two-sided Shapiro–Wilk tests; control versus treatment comparisons were performed using two-sided Wilcoxon rank-sum tests, and LMM pairwise comparisons used Tukey-adjusted EMMs (See Supplementary Data 9). Box plots show median values (black full lines), the first to third interquartile ranges (box), whiskers indicate 1.5x IQR. Single data points represent mean rosette density per animal. Significance codes for model adjusted *P*-values: **:*P* < 0.01, ns non-significant. Abbreviations: dpr days post-refeeding. Scale bars: 50 µm **A** and 10 µm (**B**–**D**).

To gain mechanistic insights into cell extrusion in *Nematostella* and enable molecular comparisons with bilaterians, we studied the role of ERK signalling during extrusion (Fig. 4 and Supplementary Figs. 6, 7). In vertebrates and flies, the ERK pathway is induced in rosette cells, coordinates the spatiotemporal dynamics of extrusion and protects the surrounding epithelium from pro-apoptotic signals^15,16^. We tested whether ERK activity in rosettes is evolutionary conserved in *Nematostella* by performing immunolabelling of phosphorylated ERK1/2 (pERK1/2; Fig. 4A–D” and Supplementary Fig. 6A–C”’) using a commercial antibody previously validated in *Hydra*^45^. Using this antibody, we found pERK1/2+ cell clusters broadly co-localising with early to late-stage rosettes throughout the epidermis (Fig. 4A–D”’). Manual quantification showed that ~89% of all rosettes identified by F-actin labelling were pERK1/2+ (*n* = 78/88; 95% CI: 80-94%; Fig. 4B’, C’, D’ and and Supplementary Data 1). These observations support a strong association between ERK1/2 signalling activity and rosette formation beyond early stages. While the central, extruding cell lacked any detectable pERK1/2 signal (Fig. 4C’, white arrowhead), the outer pERK1/2 signal boundary was often not sharply delimitated, with weaker labelled cells found beyond the rosette cells (Fig. 4C’).

To assess the function of ERK during cell extrusion, we used the small molecule U0126 inhibitor, which was previously validated in *Nematostella* larvae as an inhibitor of the mitogen-activated extracellular signal-regulated kinase (MEK) that directly phosphorylates ERK1/2^46^. We confirmed that a 2-h treatment with 20 µM U0126 effectively decreased pERK levels below the detection limit also in the juvenile epidermis (Supplementary Fig. 7A–B’). Under these short-term experimental conditions, we found that MEK inhibition at 5 dpr, when cell and rosette densities are high, caused a significant increase in rosette density compared to the 0.5% DMSO-treated control (+88% change, Tukey-adjusted *p*-value = 0.0027) (Fig. 4E). At 20 dpr, when cell and rosette densities have declined, MEK inhibition led only to a mild, non-significant increase in mean rosette density (by ~14%; *p* = 0.57) (Fig. 4E). In contrast to rosette density, the mean cell area of large epidermal cells was not significantly different between U0126- and DMSO-treated polyps at 5 or 20 dpr (Supplementary Fig. 7C). The U0126-induced increase in rosette density is thus not indirectly caused by changes in tissue density. Our findings indicate that ERK signalling regulates cell extrusion density independently of tissue density in the *Nematostella* epidermis. Together, our results highlight ERK signalling activity in rosette cells as an evolutionary conserved feature of cell extrusion between *Nematostella*, flies and vertebrates.

### Caspase-mediated apoptosis is required for cell extrusion

**Fig. 5:**
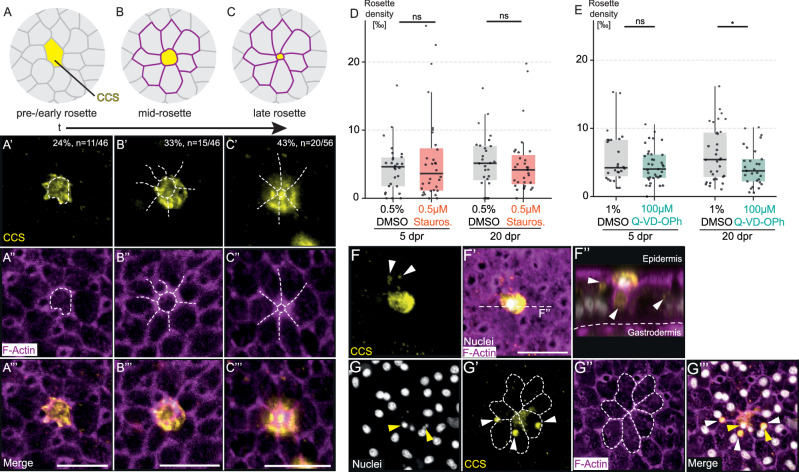
Caspase-mediated apoptosis is a hallmark of the extruding cell. Schematics (**A**–**C**) summarising the results of projections of confocal microscopy images depicting localisation of Cleaved Caspase substrate (CCS)-immunolabelled cells (**A’**, **A**”’, **B**’, **B**”’, **C**’, **C**”’) within the central cell of an F-actin-labelled rosette (**A**”–**A**”’, **B**”–**B**”’, **C**”–**C**”’). *n* = number of individual F-actin rosettes identified across images from more than three independent experiments (see Supplementary Data 1). Box plots show mean rosette density (i.e., rosette number per 1000 large cells) in the epidermis of polyps at 5 or 20 days post-refeeding treated for 2 h in the apoptosis inducer Staurosporine (**D**), the pan-Caspase inhibitor Q-VD-OPh (**E**) or in DMSO as control (**D**, **E**). Normality was assessed using Shapiro–Wilk tests, control versus treatment comparisons were performed using two-sided Wilcoxon rank-sum tests and LMM pairwise comparisons used Tukey-adjusted EMMs (see Supplementary Data 11, 13). **D** Induction of apoptosis by Staurosporine had no significant effect on rosette density at either timepoint. **E** Pan-caspase inhibition caused a decreasing trend in rosette density at both timepoints tested, which was only statistically significant at 20 dpr. Projections (**F**, **F’**) and *z*-axis reconstruction (**F**”) of confocal imaging stack of CCS/F-actin co-labelled epithelial cells showed apically extruded CCS+ cell, and presence of putative phagocytic vesicles containing CCS+ apoptotic cell debris in adjacent cells (white arrowheads). Dotted line in **F**’ depicts the plane of reconstruction in (**F**”). **G**–**G**”’ Projections of confocal imaging stack of CCS/F-actin/nuclear stain co-labelled epithelial cells showed the presence of putative phagocytic vesicles containing CCS+ (white arrowheads), including nuclear fragments (yellow arrowheads), in adjacent cells. Significance codes for model adjusted *P*-values: * *P* < 0.05, ns non-significant. Box plots show median values (black full lines), the first to third interquartile ranges (box), whiskers indicate 1.5x IQR. Abbreviations: dpr: days post-refeeding, Stauros.: Staurosporine. Nuclear stain: Hoechst. All scale bars: 10 µm. Each experiment in **A**’–**C**”’ and **F**–**G**”’ was performed independently three or more times with similar results.

To test whether apoptosis drives the cell death observed during *Nematostella* cell extrusion, we assayed morphological and molecular markers that have been associated with different stages of apoptosis in bilaterians. We first studied p38 mitogen-activated protein kinase (p38 MAPK) signalling, which has been linked to apoptosis in *Drosophila*, planarians and mammals^47–49^. Using a phospho-specific p38 antibody (p-p38) previously validated in the cnidarian *Hydra* to detect p38 activity^45^, we found that p-p38+ cells often exhibited condensed or fragmented nuclei, indicative of late apoptotic states (Supplementary Fig. 6A–A”). Co-staining with the pERK1/2 antibody further confirmed that at least some p-p38+ cells were surrounded by pERK1/2+ cells within an extrusion rosette (Supplementary Fig. 6B–C”’). To capture earlier stages of apoptosis, we used an antibody detecting the conserved Caspase cleavage substrate motif (CCS) that becomes exposed upon Caspase-catalysed proteolysis^50,51^. After aligning fixed, F-actin-stained rosettes along the proposed staging system, we found CCS+ central rosette cells throughout all stages starting at pre- or early rosette stage (Fig. 5A–C”’). At the earliest stage, CCS+ cells were of comparable size to their neighbours, making it difficult to determine if rosette constriction had already been initiated (i.e., early rosette) or not (i.e., pre-rosette) (Fig. 5A–A”’). At late rosette stages, we observed that some extruded CCS+ cells were retained at the epithelial surface (Fig. 5F–F”). Visual counting revealed that ~81% of F-actin-defined rosettes (*n* = 46/57; 95% CI: 69–89%) contained a central CCS+ cell (Fig. 5A’, B’, C’ and Supplementary Data 1), indicating that epithelial cell extrusion in the *Nematostella* epidermis is predominantly associated with Caspase-mediated apoptosis.

These observations prompted us to study the role of apoptosis during cell extrusion using Staurosporine, which induces apoptosis by non-selective inhibition of protein kinases^52,53^. We confirmed that 0.1 µM, 0.5 µM and 1 µM Staurosporine consistently induced apoptosis in the *Nematostella* body column epidermis in a concentration-dependent manner (Supplementary Fig. 8A–D’). Incubating polyps at 0.5 µM Staurosporine for 2 h predominantly induced apoptosis in single, spatially dispersed cells, allowing us to test whether isolated apoptotic cells can induce rosette formation (Supplementary Fig. 8C, C’). This treatment led to no significant changes in rosette density compared to 0.5% DMSO-treated controls at 5 dpr or 20 dpr (Fig. 5D). In contrast, Staurosporine caused a decrease in mean cell area at both 5 dpr (model adjusted *p*-value = 0.09) or 20 dpr (model adjusted *p*-value = 0.043) that may have resulted from cell loss by apoptosis (Supplementary Fig. 8G). Notably, Staurosporine treatment induced a broad increase in ERK activity across the epidermis, indicative of a global stress response^15^ (Fig. 5E–F’). Under these conditions, the lack of increased extrusion indicates that apoptosis per se is not sufficient to initiate epidermal cell extrusion in *Nematostella*.

To further explore whether apoptosis is essential for the initiation or progression of cell extrusion in *Nematostella*, we used the pan-Caspase inhibitor Q-VD-OPh to test whether blocking apoptosis affects rosette density^54,55^. Using confocal imaging, we confirmed that incubation in 100 µM Q-VD-OPh over 2 h robustly decreased epidermal CCS immunolabelling compared to 2% DMSO-treated controls (Supplementary Fig. 8H–I’). Under the same conditions, we found the inhibition of apoptosis suppressed rosette density at both 5 dpr (−20%, model-adjusted *p*-value = 0.17) and 20 dpr (−31%, model-adjusted *p*-value = 0.018) (Fig. 5E). Cell density, however, was not significantly affected between Q-VD-OPh and DMSO controls at any timepoint (Supplementary Fig. 8J). Together, our results suggest that Caspase-mediated apoptosis is necessary but not sufficient to trigger epithelial cell extrusion in the *Nematostella* epidermis.

### Nutrient recycling by phagocytosis of extruding cells?

**Fig. 6:**
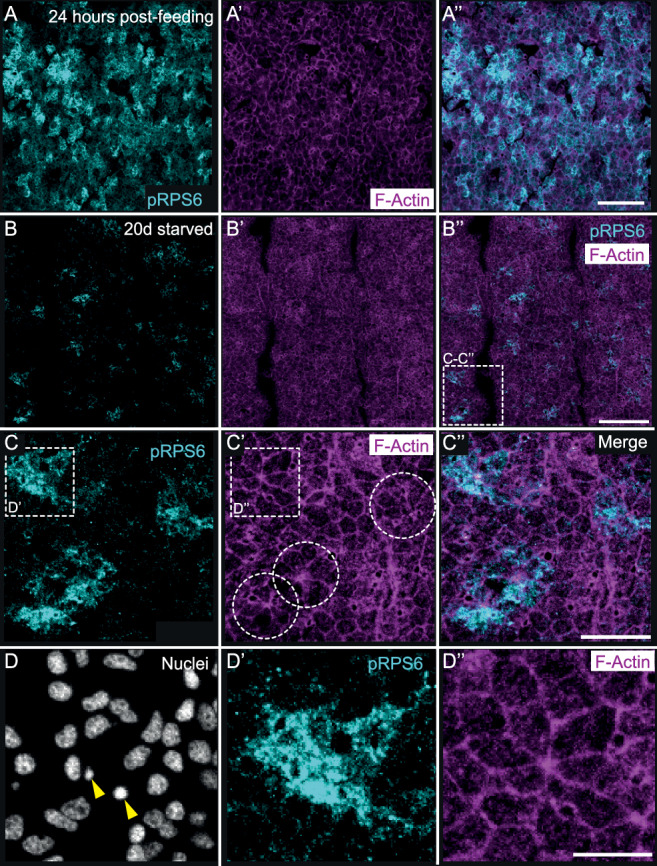
TOR signalling activation in epidermal rosettes of starved polyps. **A**–**D**” Projections of confocal imaging stacks of fixed epidermis labelled for phosphorylated Ribosomal Protein S6 (pRPS6), F-actin and nuclei in polyps at 24 h (**A**–**A**”) or 20 days post-feeding (**B**–**D**”). While pRPS6 signal, a readout for TOR signalling activity, was broadly distributed across the epidermis of juvenile *Nematostella* polyps (**A**–**A**”) at 24 h post-feeding, it was restricted to late extrusion rosettes after 20 days of starvation (**B**–**D**”). Notably, some pRPS6+ cells contained condensed nuclear fragments (yellow arrowheads), reminiscent of the CCS+ vesicles (Fig. 5G–G”’). Nuclear stain: Hoechst. Scale bars: 50 µm (**B**), 20 µm (**A**, **C**) and 10 µm (**D**). Each experiment in **A**’–**D**” was performed independently at least three or more times with similar results.

During late rosette stages, we occasionally observed CCS+ intracellular vesicles of 1–2 µm located within rosette cells (Fig. 5G–G”’ white arrowheads). These observations indicate efferocytosis, the engulfment and phagocytosis of apoptotic material by neighbouring rosette cells. It also intriguingly suggests that cell extrusion contributes to nutrient recycling during starvation. To test this assumption, we studied Target Of Rapamycin (TOR) signalling activity, a nutrient-sensitive regulator of anabolic processes (e.g., protein translation) conserved across fungi and animals^56^. Active TOR signalling was detected by immunolabelling of phosphorylated ribosomal S6 protein (pRPS6), a widely conserved TOR signalling readout previously validated in *Nematostella*^35,57^. As expected, we found broad epidermal pRPS6 staining in juvenile polyps at 24 h after feeding (Fig. 6A–A”). In contrast, after 20 days of starvation the pRPS6 signal was confined to distinct epidermal clusters overlapping almost specifically with late rosette stages (Fig. 6B–D”). The pRPS6 immunolabel was largely restricted to cells of late rosette stages (~92%; *n* = 22/24; 95% CI: 74–98%), with only few pRPS6+ rosettes observed at mid-rosette stage (*n* = 2/24). Notably, ~71% of pRPS6+ rosette cells contained condensed nuclear fragments (*n* = 17/24; 95% CI: 51–85%), reminiscent of CCS+ vesicles (Fig. 6D, yellow arrowheads).

To test whether TOR signalling is required for rosette formation, we assessed the effect of AZD8055, a small molecule inhibitor of TOR complexes 1 and 2, on extrusion density^35,58,59^ (Supplementary Fig. 9A–D). We found that incubating juvenile polyps in 1 µM AZD8055 for 2 h strongly decreased epidermal pRPS6 levels (Supplementary Fig. 9A–B’) but did not significantly affect rosette or tissue densities compared to 0.5% DMSO controls (Supplementary Fig. 9C, D). Altogether, these data indicate that activation of TOR signalling is downstream of rosette formation and may result from nutrient recycling.

## Discussion

Epithelia are found across all animal taxa, yet the evolutionary origins of their plasticity and regulation by metabolic cues remain poorly understood. In this study, we used the epidermis of the sea anemone *Nematostella vectensis* as a tractable system to investigate the cellular mechanisms underlying starvation-induced cell loss and body shrinkage. Juvenile polyps lose more than half of their cells within three weeks of starvation, whereas refeeding triggers a pronounced proliferative burst from previously quiescent cells^30,35^. These observations prompted us to investigate how the epithelium accommodates such extensive cell loss without compromising epithelial integrity. In vertebrates and *Drosophila*, epithelial cell extrusion plays a central role in maintaining epithelial homeostasis and integrity^31,60^. Using live imaging of a transgenic knock-in reporter line expressing the β-Catenin-mOrange2 fusion protein to visualise AJs, we discovered and characterised epithelial cell extrusion in the *Nematostella* epidermis.

**Fig. 7:**
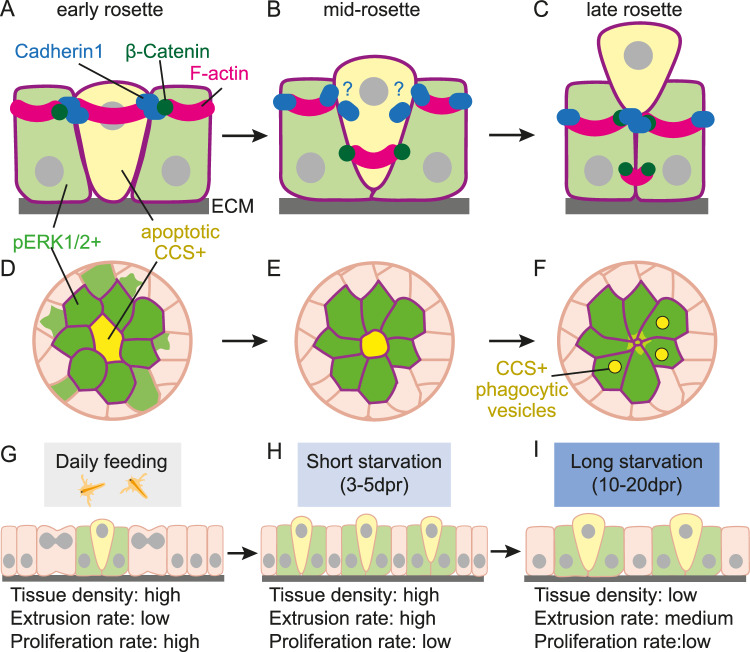
Models of cellular and tissue changes associated with epidermal cell extrusion in *Nematostella*. Schematics highlighting changes in adherens junction components (**A**–**C**; cross-section), ERK1/2 and Caspase signalling-associated changes (**D**–**F**; apical view), and epithelial dynamics associated with cell extrusion (**G**–**I**; cross-section) in the body wall epidermis of *Nematostella*. **A**–**C** During extrusion, F-actin and β-Catenin translocate from their apical position to form a basal, constricting ring, likely providing force for apical extrusion. It remains unclear whether their translocation is specific to the extruding cell or also occurs also in surrounding rosette cells, and whether Cadherin1 proteins likewise translocate extrusion. **D**–**F** ERK signalling is highest within rosettes but is active at lower levels beyond. The extruding cell undergoes apoptosis as indicated by cleaved Caspase substrate (CCS) immunolabelling, nuclear fragmentation and p38 signalling. Late rosettes occasionally contain CCS+ phagocytic vesicles and active TOR signalling, indicating nutrient recycling by efferocytosis (**F**). **G**–**I** Schematic model of the dynamic changes in tissue and rosette density change after a feeding and during starvation. A 1-h feeding pulse triggers cell proliferation within 48 h (**G**), leading to tissue crowding and increased extrusion density (**G**, **H**). As starvation progresses, the tissue density decreases, but rosette density remains at intermediate levels indicating continuous, starvation-induced cell loss (**I**).

Together with F-actin labelling and immunostaining of *Nematostella* Cadherin-1 and pERK1/2, we found that extrusion involves the formation of pERK1/2+ rosette cells, basal translocation of AJ-associated F-actin and β-Catenin-mOrange2, and predominantly apical extrusion of a central apoptotic cell. Time-lapse imaging revealed four successive stages defined by the degree of junctional constriction and the apical-basal position of F-actin and β-Catenin (Figs. 2 and 7).

In vertebrates and flies, a cell-autonomous contractile F-actin ring in the extruding cell and a supracellular F-actin ring undergoing apical-basal translocation in surrounding cells are considered hallmarks of the extrusion process^7,13^. However, comparisons between *Drosophila* and different vertebrate systems revealed a substantial diversity in the timing, direction and coordination of ring contraction and AJ remodelling^40,61–63^. In *Nematostella*, our current methods did not allow us to resolve whether the translocating F-actin and β-Catenin rings reside primarily in the central cell, in neighbouring rosette cells or in both. Nonetheless, our data suggest that translocating AJs may actively contribute to extrusion: the basal shift of both F-actin and β-catenin during constriction, together with dynamic interactions involving Cadherin-1, likely maintains the epithelial seal while pushing the extruding cell apically (Fig. 7). Extrusion directionality also shows substantial variation across animals, developmental stages and tissues^64^. Vertebrate epithelia generally shed cells apically^7,8^, whereas delamination in the *Drosophila* larval epidermis typically occurs basally^65^. However, exceptions such as the apical extrusion in the fly gut^31^ and bidirectional extrusion in the *Xenopus* embryonic ectoderm^66^ highlight that the direction of extrusion is also context-dependent. In *Nematostella*, we predominantly observed apical extrusion events, although basal or lateral extrusion may also occur but be more difficult to detect in our imaging setup.

Another molecular hallmark of extrusion in flies and vertebrates is the wave-like activation of the ERK pathway in surrounding rosette cells. During extrusion, ERK1/2 signalling coordinates the spatial distribution of rosettes and provides a pro-survival signal that protects the surrounding epithelium from initiating additional apoptotic extrusion events^15,16,67^. In *Nematostella*, we observed consistently high levels of pERK1/2 in surrounding rosette cells from early stages through extrusion, whereas pERK1/2 remained undetectable in central cells. In some cases, weaker pERK1/2 signal extended beyond the rosette into adjacent cells. Whether this variable spatial extent reflects different phases of a potential wave-like ERK1/2 phosphorylation^15^ or a pulse-like signalling pattern^16^ will require future in vivo imaging studies using ERK activity sensors^68,69^.

Pharmacological inhibition of ERK1/2 phosphorylation using the MEK inhibitor U0126 increased rosette density, supporting a role for ERK1/2 in spatially restricting extrusion events. However, because our analyses relied on single time-point measurements, we cannot exclude the possibility that MEK inhibition prolonged the duration of extrusion events, which would have also increased apparent rosette density. Developing a segmentation and tracking pipeline for time-lapse imaging, similar to those developed for *Drosophila* pupal notum or wing disc^16,70^, would enable quantitative analysis of extrusion dynamics and help resolve this question. We also observed that increased rosette density was not accompanied by a change in cell density. This is unlikely to be explained by compensatory proliferation, as ERK inhibition typically reduces cell proliferation rates in *Nematostella*^71^ and other systems^72^. Instead, the 2-hour incubation, intended to capture acute effects while minimising secondary effects, may have been too short to result in a measurable decrease in cell density despite increased cell loss.

In vertebrates and flies, the extruding cell consistently undergoes apoptosis, but the causal relationship between the Caspase-mediated apoptosis initiation and cell extrusion initiation remains debated. For example, effector Caspase activation precedes and is required for extrusion in the *Drosophila* pupal notum^10,73^, whereas in *C. elegans*, extrusion can occur independently of Caspases^9^. In *Nematostella*, the central rosette cells consistently exhibited condensed or fragmented nuclei, activated p38 MAPK signalling and high levels of cleaved Caspase substrate (CCS) immunolabelling. Phospho-p38 has been associated with apoptotic cells in *Hydra*, planarians and mice^45,48,49^, and CCS likewise marks apoptotic cells in human cancer cell lines and in *Nematostella*^50,51^. Because CCS+ cells appear already during early, and possibly even pre-rosette stages, Caspase-mediated apoptosis in *Nematostella* likely precedes extrusion initiation, consistent with observations in the *Drosophila* pupal notum^10^. This timing argues against a major role for anoikis, in which loss of basal membrane attachment induces apoptosis^12^.

Experimentally inducing apoptosis with Staurosporine, which produced predominantly isolated apoptotic events in the context of a global stress response, did not alter extrusion density, indicating that apoptosis alone is not sufficient to trigger cell extrusion. This contrasts with mammalian cell lines, where apoptosis induction increases the frequency of extrusion^7,74^, and suggests that cell loss in *Nematostella* may also occur through alternative, rosette-independent expulsion mechanisms (e.g., by T1 or T2 transition remodelling^75^). It also remains possible that Staurosporine-induced apoptosis accelerates extrusion, which would not be captured by our single time-point recordings.

Conversely, inhibiting Caspases in *Nematostella* using Q-VD-OPh reduced extrusion density, consistent with findings in fly and mammalian systems^74,76^. As with U0126 treatment, the 2-hour Q-VD-OPh incubation may have been too short for these changes to translate into measurable differences in cell density. Altogether, our results indicate that epidermal extrusion events in *Nematostella* are tightly associated with Caspase-mediated apoptosis, which appears necessary but not sufficient for extrusion initiation.

The extensive loss of cells – and thus nutritive biomass – during starvation could be partially mitigated by local nutrient recycling. Indeed, we observed intracellular, ~1–2 µm-sized vesicles containing nuclear fragments derived from CSS+ apoptotic cells within rosette cells, consistent with efferocytosis-mediated recycling of extruded material^14,77^. After prolonged starvation, epidermal TOR signalling became largely restricted to late-stage rosette cells, the majority of which (71%) contained such nuclear debris. However, TOR inhibition by AZD8055 did not affect rosette density, indicating that TOR activity is not required for extrusion. Together, these observations are consistent with a model in which efferocytosis of apoptotic material contributes to local nutrient recycling during prolonged starvation and may activate TOR signalling in rosette cells. On a cellular level, similar processes have been described in planarians, where extruded epidermal cells are engulfed and digested by intestinal phagocytes as part of ongoing tissue turnover^32,33^, and in *Hydra*, where starvation-induced cell loss is accompanied by efferocytosis of Caspase-3+ apoptotic cells in the epidermis^78,79^.

Although extrusion has been studied primarily in the contexts of development and disease, its role in feeding-dependent tissue or body plasticity remains poorly understood. Starvation-induced intestinal shrinkage has been documented in snakes, mice and *Drosophila*, but a dependence on extrusion-driven cell loss has only been demonstrated for *Drosophila* midgut shrinkage^23,31^. We therefore developed a machine-learning-based pipeline to quantify extrusion dynamics in *Nematostella* and assess their potential contribution to starvation-induced tissue remodelling.

Our results reveal a complex relationship between cell extrusion, proliferation and tissue density in the *Nematostella* juvenile epidermis. Although extrusion remained low during daily feeding despite relatively high tissue density (Fig. 7G), a marked increase in extrusion occurred following refeeding after five days of starvation (*T*_5ds_; Fig. 7H). The absence of a preceding increase in tissue density argues against a simple density-driven mechanism. Instead, we propose that increased extrusion reflects a transient mismatch between proliferation and the tissue’s capacity to accommodate newly added cells. One possible explanation is that rapid, refeeding-induced proliferation is not matched by extracellular matrix production, potentially due to limited nutrient availability after a short refeeding pulse. In contrast, low extrusion at *T*_5ds_, when proliferation is low and tissue density remains comparable to fed animals, may reflect a state in which tissue accommodation is not limiting. It is also possible that extrusion transiently increased prior to T_5ds_, consistent with previous observations of elevated cell loss during this period^30,35^. During prolonged starvation (Fig. 7I), extrusion persists at intermediate levels despite reduced tissue density and low proliferation^30,35^, suggesting that additional, potentially metabolic or energetic factors contribute to extrusion in this regime, as recently reported in mammalian systems^80^. Together, these observations suggest that extrusion dynamics are shaped by multiple interacting factors, including proliferation dynamics and metabolic state, rather than tissue density alone.

Overall, we conclude that cell extrusion in the *Nematostella* epidermis shares key molecular and morphological hallmarks with extrusion in *Drosophila* and vertebrates, including rosette formation, translocation of AJ components, active ERK signalling in rosette cells and apoptosis induction in the extruding cell^7,15,16,64^. These similarities strongly support the view that epithelial cell extrusion represents an evolutionary conserved mechanism of epithelial remodelling, dating back at least to the last common ancestor of cnidarians and bilaterians. This interpretation is consistent with previously described forms of epithelial cell shedding in other cnidarians, such as continuous shedding of cnidocytes at *Hydra* tentacle tips^81,82^, or the loss of symbiont-containing gastrodermal cells during coral bleaching^83,84^. Comparable epithelial cell loss was also observed in other non-bilaterian taxa, including expulsion of apoptotic cells during sponge metamorphosis^85^ and from choanocyte chambers^86,87^, and radiation-induced cell shedding in the placozoan *Trichoplax adhaerens*^88^. Notably, placozoans, sponges and ctenophores also undergo starvation-induced shrinkage, raising the possibility that epithelial extrusion-like processes contribute to cell loss and nutrient recycling across non-bilaterian lineages^89–91^. However, because these processes have not yet been resolved at the molecular and cellular levels, it remains unclear whether they represent bona fide extrusion or distinct modes of epithelial cell removal. Future studies will be required to determine whether epithelial extrusion represents not only a conserved mechanism of tissue remodelling but also an evolutionarily ancient strategy for nutrient-dependent tissue plasticity.

## Methods

### *Nematostella* breeding and husbandry

*Nematostella vectensis* specimens originate from CH6 females and CH2 males collected at Rhode River, MA^92^. Adult males and females are kept separately in the dark at 18 °C in ~16 ppt filtered seawater (*Nematostella* medium, NM) and fed with fresh *Artemia nauplii* five times per week, with a daily partial water exchange and a monthly full cleaning of the culture boxes. Spawning is induced every three weeks triggered by temperature increase (25 °C) and a 12-h light cycle overnight^93^. Embryos are maintained at 25 °C, and feeding with *Artemia nauplii* begins approximately 7–10 days post-fertilization. Sex was not determined in juveniles; therefore, all data shown include both males and females. All experiments were performed on juvenile polyps of ∼5 mm in length, approximately 1 month after fertilisation

### Immunofluorescence

Juvenile polyps were relaxed in 0.1 M MgCl₂ in *Nematostella* Medium (NM) and then transferred to a plate containing 3.7% formaldehyde/ 0.5% dimethyl sulfoxide (DMSO) in NM (“killing solution”). They were then carefully transferred into a fixation solution of 1× Phosphate-Buffered Saline (PBS)/ 3.7% formaldehyde/ 0.5% DMSO/ 0.1% Tween-20 and fixed overnight at 4 °C. Following fixation, samples were thoroughly washed with 1× PBS/ 0.1% Tween-20 and stored at 4 °C for a maximum of 10 days. Samples were then blocked in 1× PBS/ 5% normal goat serum (NGS)/ 1% bovine serum albumin (BSA)/ 0.2% Triton X-100 for two hours at room temperature. Primary antibody incubation was performed in 5% NGS/ 1% BSA/ 0.2% Triton X-100 overnight at 4 °C. Primary antibody dilutions were used as follows: 1:200 for Cleaved Caspase Substrate, 1:50 for phospho-p38 conjugate, 1:200 for phospho-S6 ribosomal, 1:200 for phospho-ERK1/2, 1:200 for phospho-p38 and 1:500 for Cadherin1 (see Supplementary Table 1 for commercial catalogue numbers). Following overnight incubation, tissues were washed in 1× PBS/ 0.2% Triton X-100 and then blocked in 1× PBS/ 5% NGS/ 0.2% Triton X-100 for 30 minutes at room temperature. Hoechst nuclear staining (1:10,000 dilution) (ThermoFisher) and Phalloidin F-actin staining (1:50, dilution) and secondary antibodies (1:1000 dilution) (see Supplementary Table 1 for commercial catalogue numbers) incubated in 1× PBS/ 5% NGS/ 0.2% Triton X-100 overnight at 4 °C. Finally, samples were thoroughly washed in 1× PBS/ 0.2% Triton X-100, cleared in glycerol and mounted on slides (Electron Microscopy Sciences, 63418-11) in 80% glycerol. Coverslips (Menzel-Gläser, 18 × 18 mm) were sealed with clear nail polish. Exceptionally, rabbit anti-phospho-RPS6 was blocked for three nights^35^. For the Cadherin1 antibody, tissues were instead fixed for 1 h at 4 °C with Lavdovsky’s fixative (3.7% formaldehyde/ 50% ethanol/ 4% acetic acid)^36^. For double immunolabelling, polyps were incubated with phospho-p38 Alexa Fluor 594 conjugate at a 1:50 dilution along with the phospho-ERK1/2 antibody at a 1:100 dilution in blocking buffer at 4 °C overnight. The protocol then proceeded as usual, except that no secondary antibody was necessary for the phospho-p38 Alexa Fluor 594 conjugate^45^. Tissue staining and immunohistochemistry have been performed on different feeding states, including early post-feeding (1-2, 5 days starved), as well as late starvation (20 days starved). In the manuscript, exemplary images represent the results found across multiple feeding states unless a significant different result was found between fed and starved states.

### Drug treatments

All pharmacological inhibitors were prepared from 100% stocks and diluted in NM to their working concentrations: AZD-8055 from 10 mM stock to 0.1 µM^35^, Staurosporine from 500 µM to 0.5 μM; U0126 from 10 mM to 20 μM, and QV-D-OPh from 10 mM to 100 μM. All treatments had additional DMSO added to a final concentration of 0.5% in NM, and the negative controls included 0.5% DMSO to match treatments (see Supplementary Table 1 for commercial catalogue numbers), except for QV-D-OPh which was used at 1% DMSO for live imaging. For all drug treatments, polyps were incubated in drug solution for 2 h at 25 °C standard culturing temperature prior to imaging. This incubation time was chosen as a compromise between allowing sufficient drug penetration and effective perturbation while minimising potential secondary effects. Because individual extrusion events occur on a timescale of ~10–15 min, the aim was to capture acute rather than long-term or potentially irreversible effects. For live imaging, animals were relaxed by some drops of 1 M MgCl_2_ added 30 min before drug removal, followed by transfer into fresh NM with 0.1 M MgCl_2_ followed by mounting into in Ibidi µ-Slide VI 0.4 (Cat. # 80606) and confocal imaging.

### Transmitted light and confocal imaging

Immunofluorescence samples were imaged using an Olympus FV3000 confocal microscope (standard PMT detectors, 63× silicon-immersion objective). Live juvenile polyps were relaxed in 0.1 M MgCl₂ in NM, mounted on an Ibidi µ-Slide VI 0.4 (Cat. # 80606) and also imaged on the Olympus FV3000 confocal microscope. Confocal room temperature was at a range between 20 and 22 °C, and therefore live imaging was performed at that temperature. Images and confocal stacks were processed, cropped, and adjusted for levels and colour balance using ImageJ/Fiji.

### CRISPR/Cas9-mediated generation of β-Catenin-mOrange2 knock-in line

The used protocols were adapted from previously established protocols for the generation of CRISPR/Cas9-mediated knock-in lines in *Nematostella vectensis*^94,95^. A single guide RNA (gRNA) was designed with a predicted cut site 14 bp upstream of the Stop codon of the *Nematostella* β-catenin gene (Nvec200_v1.10389.1.p1) using CRISPOR^96^. The gRNA template was generated by annealing and elongating an invariant T7 promotor/tracrRNA-encoding and a sequence-specific oligos^97^ (ThermoFisher, desalted, see Supplementary Table 2 for sequences). In vitro transcription of the gRNAs was performed using a T7 MegaScript Transcription Kit (ThermoFisher), followed by ammonium chloride precipitation and dilution in nuclease-free water to a final concentration of 1.5 μg/μl. The donor DNA fragment for homology-mediated repair (HDR) consisted of the 3’-end of the *β-catenin* gene open reading frame (ORF) cloned in-frame with a (GGGGS)2 linker and the mOrange2 protein-coding sequence. The construct also contained 994 bp long ‘left’ (upstream) and 936 bp long ‘right’ (downstream) homology arms (see Supplementary Table 2 for sequences). This entire construct was cloned into a pJet1.2 plasmid backbone (ThermoFisher) using Gibson Assembly Master Mix (NEB)^98^. The donor DNA fragment was PCR-amplified using biotin-labelled oligos (ThermoFisher; desalted, see Supplementary Table 2 for sequences) flanking the donor fragment to enhance integration efficiency^99^. Cas9-mediated knock-in injections were carried out by modifying existing protocols for CRISPR/Cas9-mediated mutagenesis, using 0.75 μg/μl TrueCut Cas9 protein v.2 (Thermofisher), 75 ng/μl of each guide RNA, 70 ng/μl column-purified donor DNA, and a modified injection buffer containing 220 mM KCl^100^. Approximately 1600 zygotes were injected, and the resulting juvenile polyps displaying mOr2+ signal were selected and raised to sexual maturity. At the adult stage, polyps exhibiting mOrange2 signal in the developing gametes were chosen as founder animals and outcrossed with wild-type animals to generate the F1 generation. The successful integration of the *linker-mOrange2* fragment knock-in was validated at the transcript level by cDNA synthesis from total RNA extracted from a mix of larvae tissue resulting from a cross of two heterozygous F1 animals. Using this cDNA, regions spanning the transition between the 3’ UTR and mOrange2-encoding region, and between the mOrange2-encoding region and the 3’ end of the *β-catenin* ORF were amplified using oligos outside the homology arm regions (see Supplementary Table 2). Sanger sequencing of PCR fragments following gel elution and column purification confirmed the flawless, in-frame integration of the *linker-mOrange2* fragment into the locus. The successful, flawless genomic integration was further validated at the genomic level by extracting genomic DNA from mixed tentacle clips of several homozygous F2 animals from the same founder F1 animals. Based on this genomic DNA, the transitions between the 3’-end of the *β-catenin* ORF and the (GGGGS)2 linker/mOr2 and between and the 3’ end of the mOr2 ORF and the 3’UTR of the *β-catenin* gene were amplified using oligos outside the homology arm regions (see Supplementary Table 2 for sequences). Gel elution and Sanger sequencing confirmed successful integration.

### Image acquisition and experimental design

Data acquisition was performed using a confocal microscope. For each β-catenin-mOrange2 transgenic juvenile *Nematostella vectensis* polyp, two to three sets of image stacks were acquired, focusing on the epidermal cell layer. Approximately 10–15 animals per treatment condition or timepoint were imaged to ensure sufficient statistical power. In the starvation experiment, animals were initially starved for five days following continuous daily feeding (T_5ds_) to control for their feeding history. They were sampled at *T*_5ds_ or refed for 1 h and subsequently sampled at the following timepoints: 1 day post-refeeding (dpr) through to 5 dpr, 10 dpr, and 20 dpr. At 20 dpr timepoint, animals were fed again for 1 h and sampled twenty-four hours later to obtain a final timepoint (+24 h). This starvation experiment was conducted with two biological replicates.

For the drug treatments, animals were starved for 5 days (*T*_5ds_) fed for 1 h and then starved for either 5 dpr or 20 dpr before being treated with one of four compounds: AZD8055, U0126, QV-D-OPh, or Staurosporine (see details above for Drug treatments). Each treatment condition had 3 biological replicates and corresponding DMSO controls. In total, this resulted in two timepoints (5 and 20 dpr), four inhibitors, four respective controls, and three independent replicates per condition, a total of 937 images. As with the starvation data, two to three image stacks were acquired per animal, with approximately 10–15 animals imaged per treatment or control group.

Juvenile polyps used for the daily fed dataset were maintained under the normal daily feeding regime used for general *Nematostella vectensis* husbandry. Animals were fed daily each morning and then cleaned in the early afternoon to remove residual food and waste before imaging. Imaging was performed at the same time of the day (14.00–16.00) as all other experiments (starvation and drug) to standardise conditions across all samples. For each of the three consecutive days, a different biological batch of animals was used, providing three independent replicates. This resulted in three separate datasets representing animals under daily feeding conditions. As with the starvation and drug experiments, two to three image stacks were acquired per animal, with approximately 10–15 animals imaged per biological replicate.

### Image curation and cell segmentation pipeline

Following image acquisition, the image stacks were manually curated to select the most appropriate z-planes, specifically those where the surface of the epidermis was best visualised. Image z-stacks were then edited into 2D maximum intensity projections with the use of ImageJ/Fiji^101^ to improve the coverage of the epithelium especially in cases of capturing areas in which the epidermis was not entirely flat while also improving the focus of the image. These curated images were then subjected to initial segmentation using Cellpose software (https://www.cellpose.org)^42^. For the time-series dataset, approximately 250 of 500 images were manually corrected after initial segmentation to refine cell boundaries and eliminate segmentation errors. These manually corrected masks were then used to train a custom Cellpose Cyto3 base model, which improved performance and was subsequently used to segment the remainder of the time-series dataset as well as the inhibitor dataset. Post-segmentation processing included computational quality control steps to remove background artefacts, identify unsegmented regions, and classify cells based on size. Segmented objects below a defined pixel area threshold were assumed to be “oversegmented” or background noise and were merged with neighbouring cells. Conversely, objects with abnormally large areas were excluded as background or segmentation artefacts (see further details in Supplementary Notes). The resulting processed masks were used for further analysis.

### Rosette annotation and prediction

For automatic rosette segmentation, we trained an Attention U-Net deep learning model^102^ on a manually annotated (i.e. human-in-the-loop workflow) subset of 268 time-series images. To prioritise multicellular geometric features over intensity cues, the model was trained exclusively on two geometric inputs derived from cell segmentations: binary cell boundary maps and morphologically dilated boundaries rather than raw fluorescence. Model performance was monitored on a 20% held-out validation set using Dice coefficient. Following inference, all predictions underwent manual review and correction. We quantified the method’s efficiency by calculating the speed-up factor of this model-assisted workflow compared to two baselines: manual identification on segmented images and manual identification on raw data (see further details in Supplementary Notes). To ensure reproducibility and facilitate scalable processing across all datasets, the entire analysis workflow—from segmentation to quantification—was orchestrated using a custom pipeline with managed software environments (see further details in Supplementary Notes).

### Statistical analysis and graphical representation of data

Data visualization and analysis were performed using R^103^ (v. 4.5.2 (2025-10-31), https://www.r-project.org/, see Supplementary Notes for packages used). All statistical tests were two-sided. Timepoint and treatment condition were treated as fixed effects, with replicate and animal identity included as random effects where appropriate; effect sizes were estimated as differences in Tukey-adjusted means with 95% confidence intervals. Cell area measurements exhibited a robust bimodal distribution consisting of two populations of small and large cells (see Zenodo public repository for raw data files 1, 3, and 5–8; 10.5281/zenodo.19726756. To statistically define these classes, a Gaussian Mixture Model (GMM) with two components was fitted to each image (see Supplementary Notes for details and public repository at Zenodo for raw data files 2, 4, 9–12; 10.5281/zenodo.19726756). Cells were classified as small or large based on Bayesian posterior probabilities. Across all datasets, the proportions of cell classes were stable (~25% small, ~75% large), and the size of the small-cell population remained invariant across treatments and timepoints. As a result, small cells were excluded from analyses of rosette density. Rosette density was calculated as the number of rosettes per 1000 large cells per image. For the rosette-density timeseries and daily fed datasets, temporal changes were assessed using linear mixed-effects models to account for replicate variability and to obtain model-adjusted means and *p*-values (see Supplementary Data 2, 5). Cell area dynamics were analysed separately for small and large cells. Cell area was also modelled using linear mixed-effects models from which estimated marginal means (EMMs) and Tukey-adjusted pairwise comparisons were extracted (see Supplementary Data 3 and 6). *P*-values from multiple comparisons were adjusted using Tukey’s method where applicable. Additional details on mixed-model fitting are provided in Supplementary Notes. For drug experiments, treated vs. control comparisons were assessed using Wilcoxon rank-sum tests and Kruskal–Wallis tests, supplemented with rank-transformed mixed models (see further details in Supplementary Notes and Supplementary Data 7–14). All reported means in the main text, *p*-values, and percentage changes refer to EMM-adjusted results. Rosette density and cell area were visualised using boxplots and violin plots, respectively. For manual/visual quantifications (see Supplementary Data 1), confidence intervals for proportions reported were calculated using Wilson’s method. Figures were exported as vector graphics (PDF), and final layouts were refined in Adobe Illustrator.

### Declaration of generative AI and AI-assisted technologies in the manuscript writing process

During the preparation of this manuscript, Large Language Models (OpenAI, https://chat.openai.com, Claude by Anthropic, https://claude.ai/ and Gemini by Google, https://gemini.google.com) were used to assist with coding and R script troubleshooting, as well as for polishing and grammar checking of the manuscript text. All outputs were critically reviewed and edited, and the authors take full responsibility for the final content.

### Reporting summary

Further information on research design is available in the Nature Portfolio Reporting Summary linked to this article.

## Supplementary information

Supplementary Information

Peer Review file

Description of Additional Supplementary Files

Supplementary Data 1-14

Supplementary Movie 1

Supplementary Movie 2

Reporting Summary

## Data Availability

The manual training dataset generated for the machine-learning pipeline to identify rosettes, pre-segmented image files, and the raw image segmentation and cell-level data generated in this study have been deposited in the Zenodo public data repository available under: 10.5281/zenodo.19726756. Transgenic lines are available upon request.

## Code availability

The complete source code and automated analysis pipeline are available under the MIT License at: 10.5281/zenodo.20479352. The trained Attention U-Net model weights are publicly hosted and accessible via Hugging Face at: https://huggingface.co/NoahBruderer/nematostella_rosette_detection.
