# Supplementary material for: Epithelial cell extrusion underlies starvation-induced cell loss in a sea anemone
Source: Nat Commun. 2026 Jun 17;17:7681. doi: 10.1038/s41467-026-74437-7 (PMC13433931; doi:10.1038/s41467-026-74437-7)
Supplement: Supplementary file 1 [file 41467_2026_74437_MOESM1_ESM.pdf]

Supplementary Information

Contains:

- **Supplementary Figures 1-9**
- **Supplementary Tables 1-2**
- **Supplementary Notes**
- **Supplementary References**

Supplementary Figures 1-9

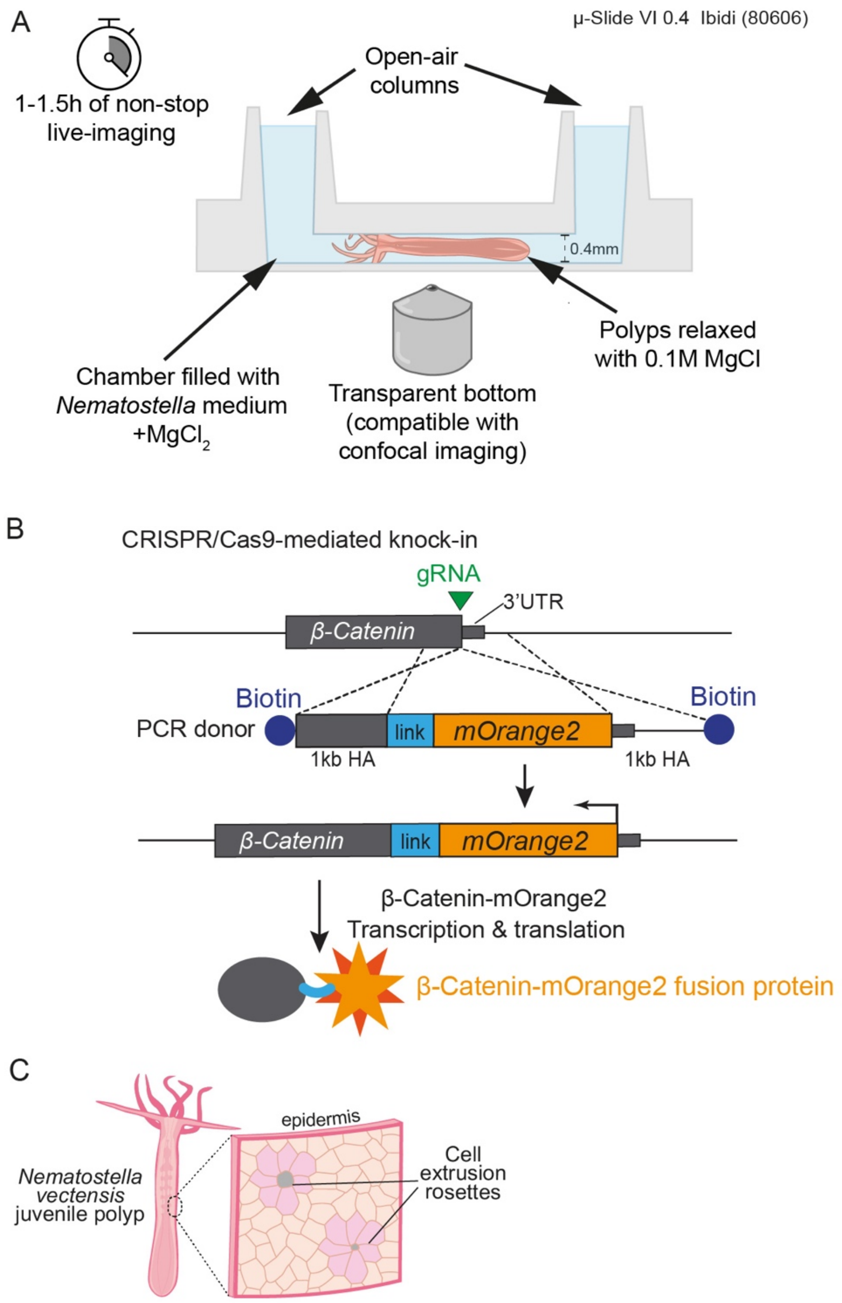

Supplementary Fig. 1. Live imaging set up and strategy for generating a β-Catenin-mOrange2 fusion protein transgenic line.

(A) Live imaging set-up for confocal imaging of *Nematostella* juvenile polyps. Juveniles were immobilised and imaged in commercially available Ibidi µ-Slide VI (80606) chambers with a 0.4mm channel height. Each chamber provides two open ports that allow continuous gas exchange, offering a more stable environment for live polyps compared to sealed coverslip preparations. (B) Schematic depicting the CRISPR/Cas9-mediated homology-directed recombination strategy to generate a β-

Catenin-mOrange2 knock-in allele. A single gRNA was used to induce a double-strand break close to the stop codon of the β-Catenin open-reading frame. Homology-directed repair was mediated by a donor construct with ±1 kb homology arms, encoding a linker ($GGGGS_2$) and encoding the mOrange2 reporter protein cloned in-frame to result in a C-terminal fusion protein. The donor DNA was biotinylated at the 5'-ends to prevent concatemerisation. Successful, flawless integration was validated on both transcript and genome levels (see Methods and Supplementary Table 2). (C) Schematic of a juvenile *Nematostella* polyp indicating the body region used for imaging of the epidermis throughout this work. Abbreviations: h: hour, gRNA, guide RNA; HA, homology arm; kb, kilobase; ORF, open reading frame and UTR, untranslated region.

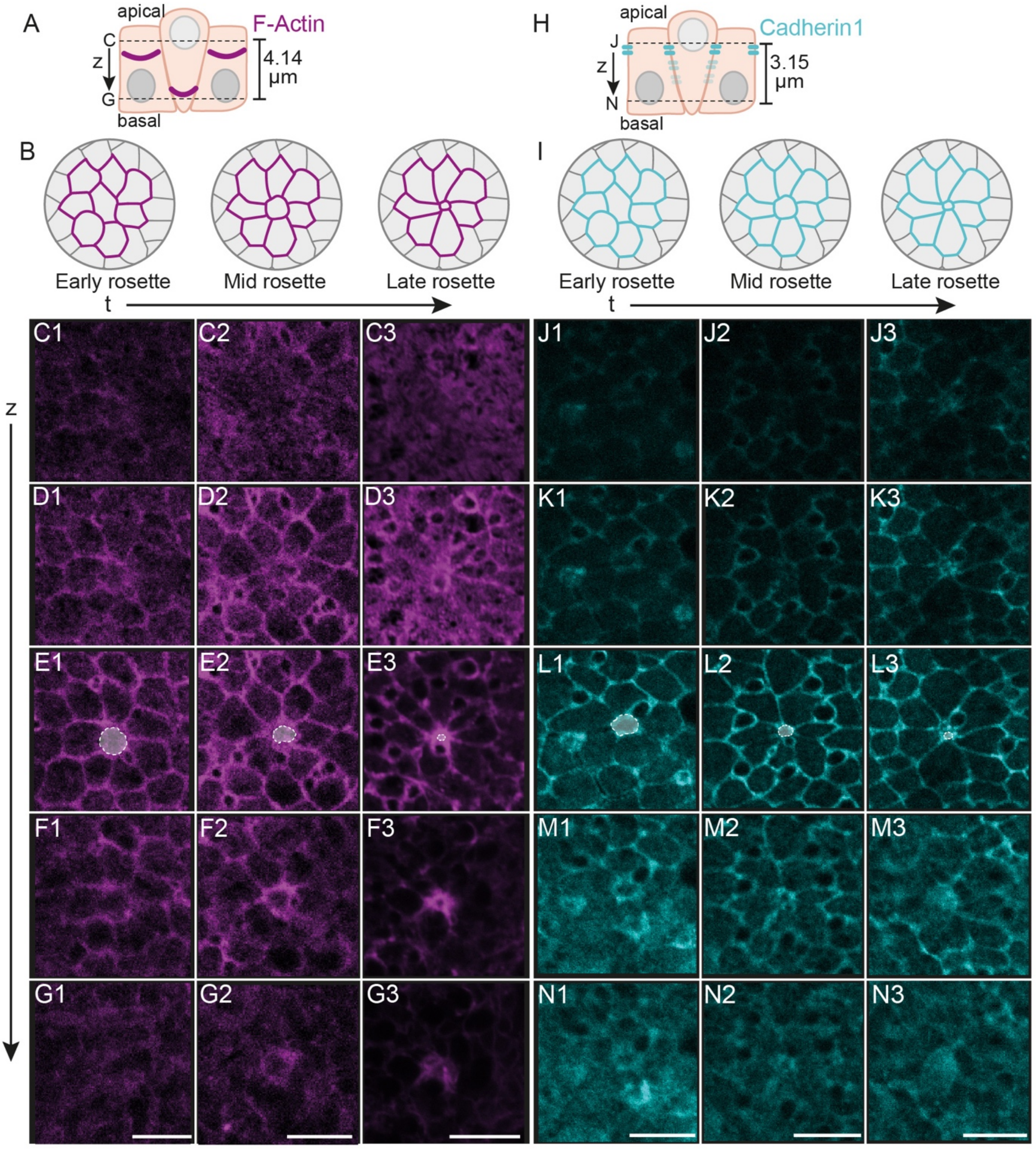

Supplementary Fig. 2. NvCadherin1 and F-actin are dynamically distributed along the apical-basal axis during cell extrusion.

(A, H) Schematics indicating the relative positions of F-actin (A) or NvCadherin1 (H) and of image panels (in C1-G3, J1-N3) along the z plane. (B, I) Schematic overviews of typical rosette shapes at early rosette, mid-rosette and late rosette stages. (C1-G3, J1-N3). Single plane confocal microscopy images of fixed juvenile body wall epidermis labelled by Phalloidin (F-actin; C1-G3) or an antibody detecting *Nematostella* Cadherin1 (J1-N3) along the z-axis from apical (C, J) to basal (G, N) and from early rosette (C1-G1, J1-N1) to late rosette stages (C3-G3, J3-N3). Shaded area in E1-E3,

L1-L3 highlight progressive constriction and extrusion of the central cell. Note the broad apical localisation of Cadherin1 and basal translocation of the central F-actin ring during extrusion. Scale bars: 10μm.

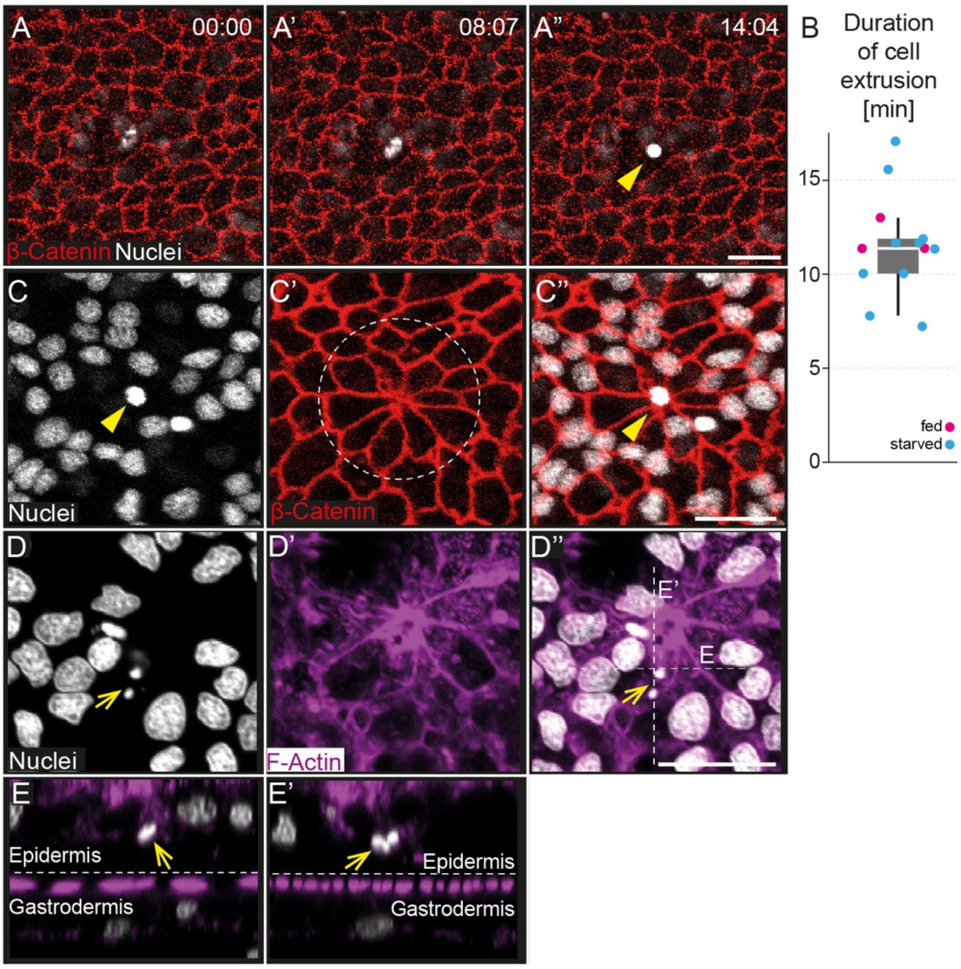

Supplementary Fig. 3. Examples of apical and putative basolateral cell extrusion events.

(A-A'', C-C'') Single plane *in vivo* confocal microscopy images of time-lapse recordings of β-Catenin-mOrange2 transgenic juvenile polyps combined with Hoechst nuclear staining. The condensed nuclei (yellow arrowheads) at the centre of a rosette indicates the apoptotic nature of the extruding cell, and its apical appearance (in A-A'') at the level of AJs indicates apical extrusion (see also Supplementary Movie 2). (B) Box plot showing the spread in duration of cell extrusion events based on *in* vivo time-lapse recordings of fed and starved juvenile polyps (n = 13 independent live recordings of rosette formation from 13 different individual polyps, representing 3 polyps about 1-2 days after feeding and 10 polyps starved for approx. 20 days) (see Supplementary Data 1). Box plot shows median value (middle bar), the first to third interquartile ranges (box) and whiskers indicate 1.5x IQR. Individual datapoints are plotted as dots, coloured depending on their feeding status (fed: fed; starved for 20 days: blue). (D–E′) Projection of confocal stack (D-D'') and z-axis reconstructions (E,E') of late rosette in juvenile polyp epidermis stained with phalloidin (F-actin) and nuclear staining

(Hoechst) reveals a basally positioned, highly fragmented nucleus (yellow arrows) indicating basal or lateral extrusion. Nuclear stain: Hoechst. Scale bars: 10µm. Time: min:sec.

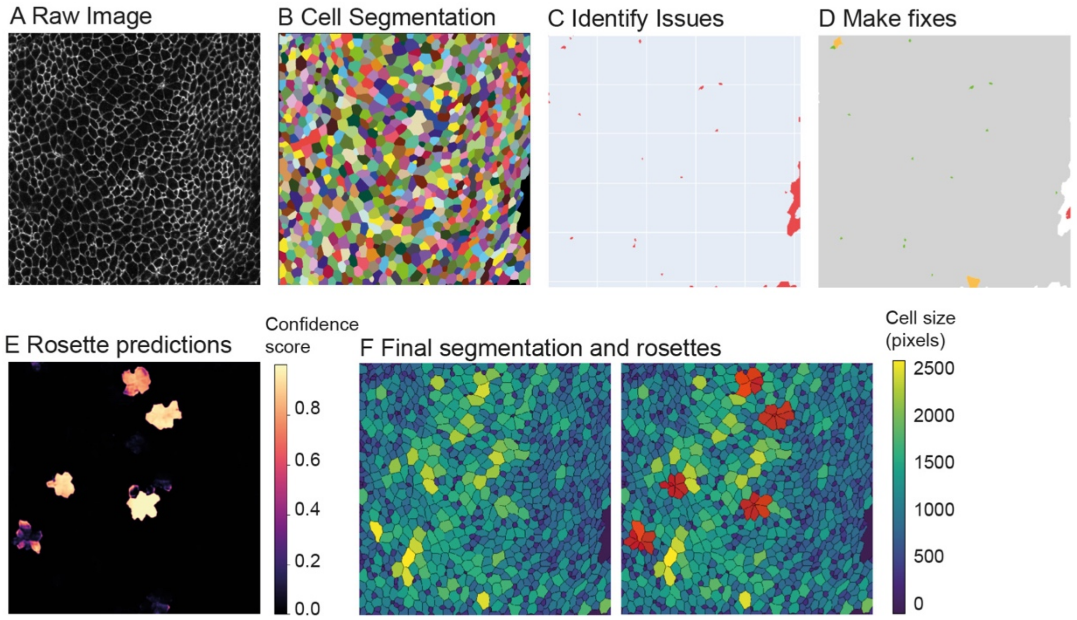

Supplementary Fig. 4. Illustrative example images of image segmentation and rosette ID pipeline from the confocal image projection to assigned rosettes and cell outlines.

(A) Projection of *in vivo* confocal microscopy image showing cell outlines labelled using a β-Catenin-mOrange2 transgenic juvenile polyp. Image represents an example used for subsequent image segmentation. (B) Result of initial cell segmentation using Cellpose on the image in (A), generating a pre-processed mask. (C-D) Quality control based on a cell segmentation image (B) to identify (C) and correct (D) for artefacts such as background, unsegmented, very small or very large cells. Cleaned mask after filtering out background, excluding large artefacts, and merging small fragments with neighbours (D). (E) Rosette likelihood map based on manually trained model. (F) Final annotated segmentation combining both cell outlines and rosette assignments. See Supplementary Data 1, 3, 5-8.

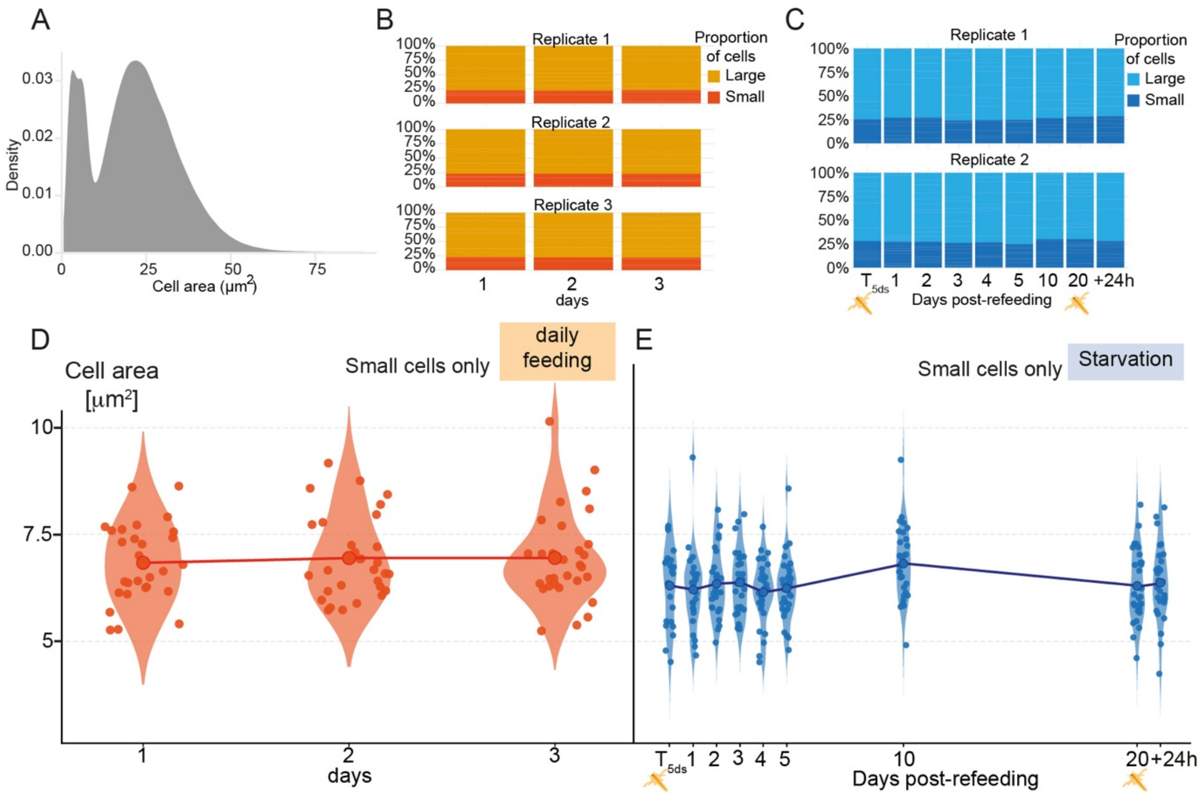

Supplementary Fig. 5. Quantification of cell area changes in daily fed and starved juvenile polyps reveals two populations of large and small cells.
(A) Binomial distribution of cell areas in the epidermis of all pooled polyps across the starvation dataset. (B, C) Bar plots show the relatively invariant proportions of cells with small (red, dark blue) and large (orange, light blue) areas per replicate in the 'daily fed' (B) or starvation (C) datasets. (D-E) Violin plots represent the distribution of small cell area during daily feeding (D) or starvation experiments (E; see Fig. 3A, B for setup). Note that the cell area of small cells stays within a narrow range across all sampling timepoints (D, E), while the cell area of larger cells changes dynamically during starvation (see Fig. 3D). Larger central dots represent means per timepoint, which are joined to the other means to form a trend line. Smaller dots represent mean small cell area per animal. See raw data files 1-4 in public repository (10.5281/zenodo.19726756) and Supplementary Data 3, 4, 6.

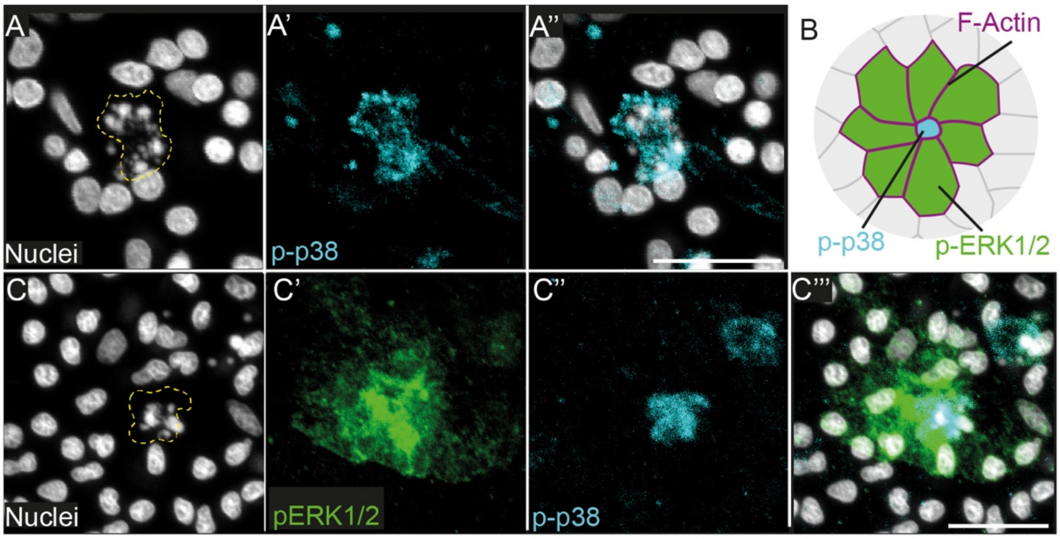

Supplementary Fig. 6. Phosphorylated p38-labelled (p-p38+) cells with fragmented nuclei located at the centre of phosphorylated-ERK1/2 (pERK1/2+) rosette.

(A–A'', C-C''') Projections of confocal imaging stacks of fixed epidermis labelled for p-p38, pERK1/2 and nuclei. Note the nuclear fragmentation (yellow dotted area) as an indication of late apoptosis in the p-p38+ cells within a pERK1/2 patch. Nuclear stain: Hoechst. Scale bars: 10μm. (B) Schematic summary of pERK1/2 and p-p38 labelling that together support that extruding cells are p-p38+.

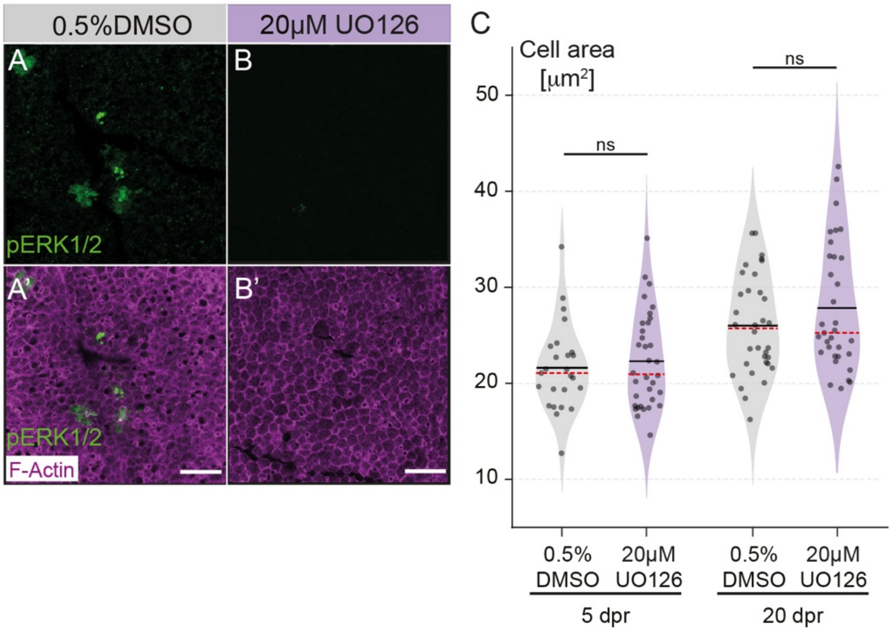

Supplementary Fig. 7. MEK inhibition by U0126 suppresses ERK1/2 phosphorylation without significantly affecting the cell area distribution of large cells.

(A-B') Projections of confocal imaging stacks of fixed epidermis of 5 days-starved polyps labelled for phosphorylated ERK1/2 (pERK1/2) and F-actin (phalloidin) after 2 hours incubation in 0.5% DMSO (A–A') or 20 µM U0126 (B–B'). Images are representative of the whole-body wall epidermis, with pERK1/2+ cell patches becoming undetectable after U0126 incubation. (C) Violin plots show the distribution of mean cell areas across larger cells in the epidermis after 2 hours of 0.5% DMSO (control) or 20 µM U0126 treatments at 5 days post-refeeding (dpr) or 20 dpr. Note that U0126 does not significantly change tissue density. Statistical significance was assessed using two-sided linear mixed-effects models (LMMs) on log-transformed mean cell area values. Pairwise comparisons of estimated marginal means (EMMs) were Tukey-adjusted. Means are represented by black full lines and medians as red dashed lines. Single data points represent means of large cell areas per animal. Significance codes for model adjusted P-values: ns = non-significant. Abbreviations: dpr: days post-refeeding. Scale bars: 20µm. See also Supplementary Data 10.

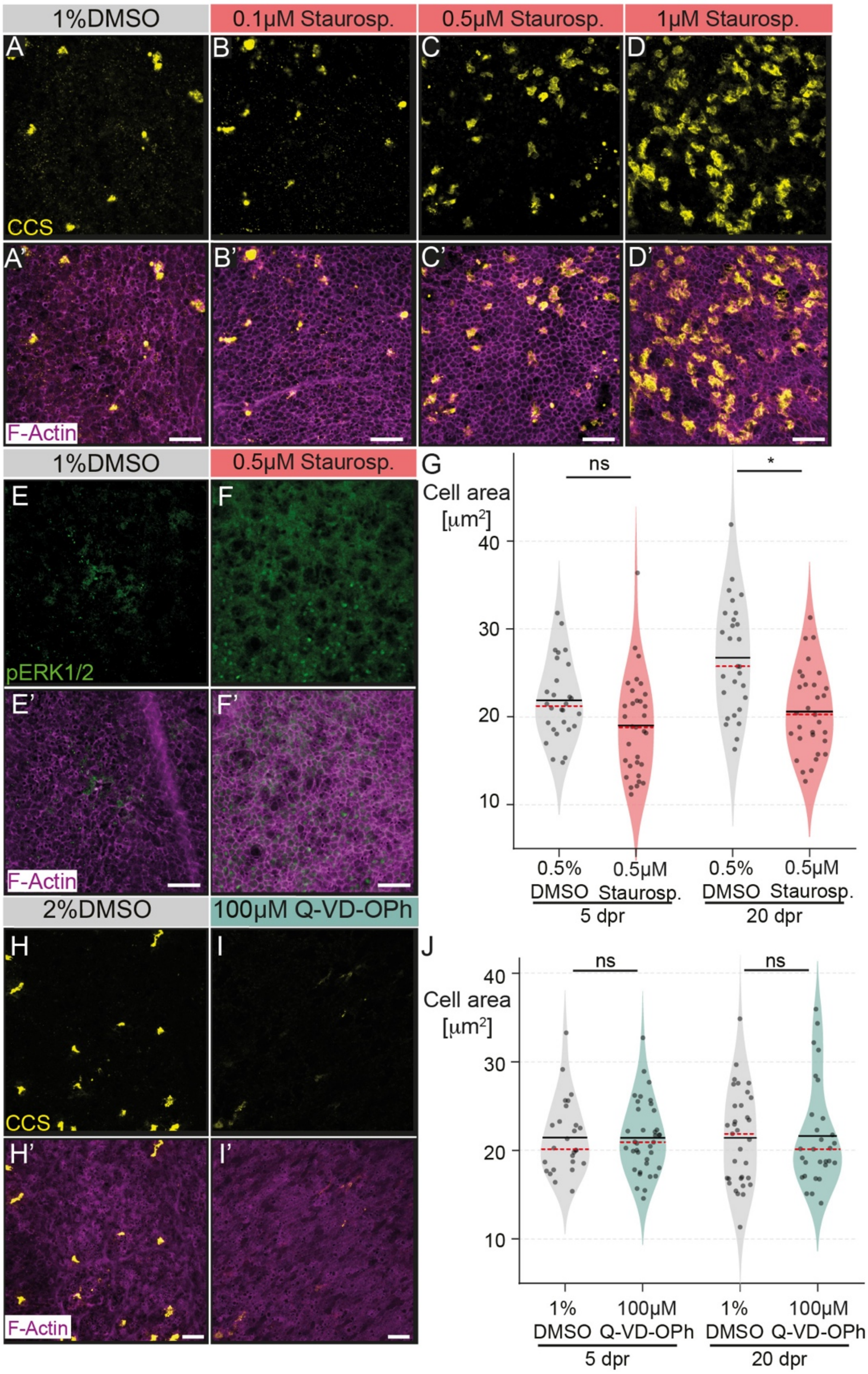

Supplementary Fig. 8. Effects of the apoptosis inducer Staurosporine and inhibitor Q-VD-OPh on the density of apoptotic cells and on cell area distributions.

(A–F′, H-I’) Projections of confocal imaging stacks of fixed epidermis of 20-day starved juvenile polyps labelled for cleaved Caspase substrate (CCS; A-D’, H-I’), phosphorylated ERK1/2 (pERK1/2; E-F’) and F-actin (Phalloidin, A’-I’) after incubation in DMSO (control), Staurosporine (6 hours) or Q-VD-OPh (2 hours). As expected,

Staurosporine incubation leads to a strong, concentration-dependent increase in CCS+ apoptotic cells (A-A') and a broad increase in pERK1/2 signal (E-F'), while Q-VD-OPh treatment strongly reduces CCS+ apoptotic cells across the epidermis (H-I'). (G, J) Violin plots show the distribution of mean cell areas across larger cells in the epidermis after 2 hours of 0.5% or 1% DMSO (control), 0.5μM Staurosporine or 100μM Q-VD-OPh treatments at 5 days post-refeeding (dpr) or 20 dpr. Mean cell areas among larger cells tended to decrease after Staurosporine treatment, which was statistically significant only at 20dpr. The mean cell areas were not significantly affected by Q-VD-OPh. Statistical significance was assessed using two-sided linear mixed-effects models (LMMs) on log-transformed mean cell area values, and pairwise comparisons of estimated marginal means (EMMs) were Tukey-adjusted. Means represented as black full line and medians as red dashed lines. Single data points represent mean large cell area per animal. Significance codes for model adjusted P-values: * $P<0.05$, ns = non-significant. Abbreviations: dpr: days post-refeeding, Staurosp.: Staurosporine. Scale bars: 20μm. See also Supplementary Data 12, 14.

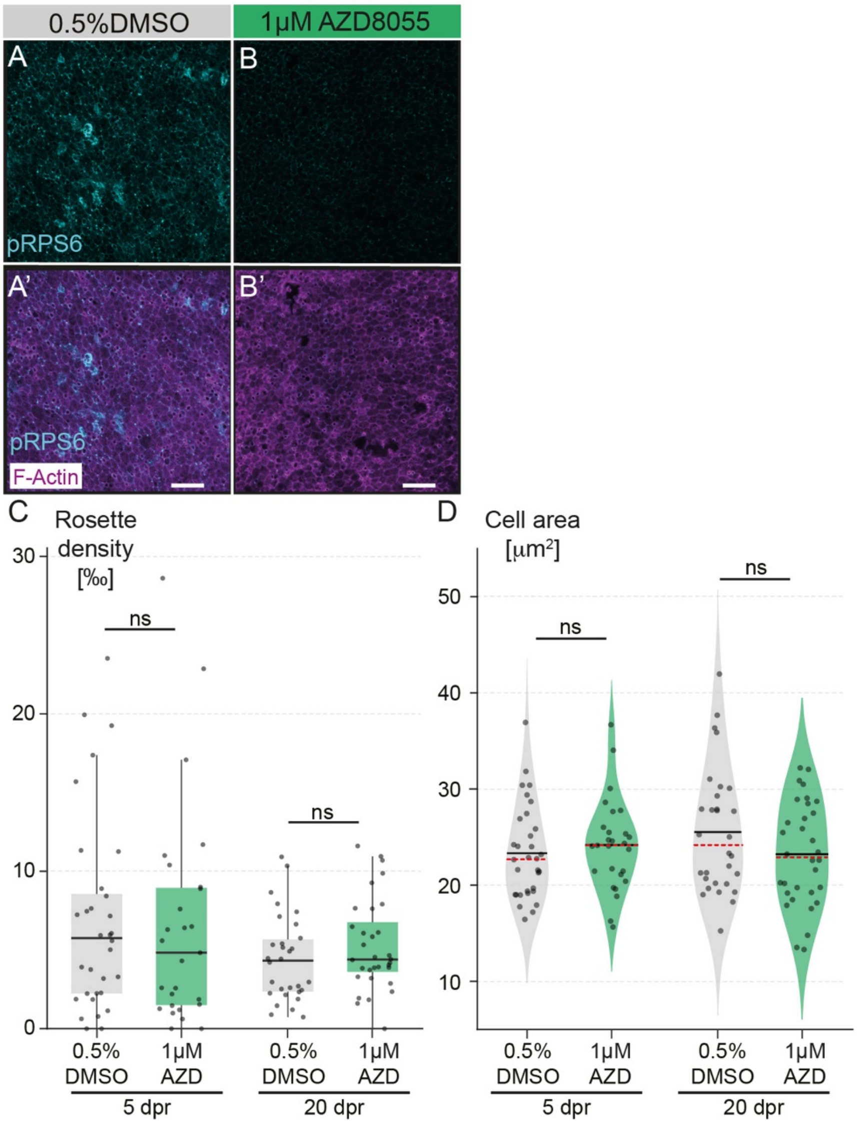

Supplementary Fig. 9. Inhibition of TOR signalling does not significantly change rosette density and cell area

(A-B') Projections of confocal imaging stacks of fixed epidermis of 5-days starved juvenile polyps labelled for phosphorylated ribosomal S6 protein (pRPS6) and F-actin (Phalloidin) after 2 hour-long 0.5% DMSO (A-A') or 1 μM AZD8055 (B-B') treatment. Representative image showing that phosphorylation of RPS6 becomes undetectable in the juvenile body wall epidermis after TOR signalling inhibition by AZD8055. Scale bars: 20μm. (C-D) Box plots (C) and violin plots (D) show the distribution of rosette densities (C) or cell areas of larger cells (D) in the epidermis after 2 hours of 0.5% DMSO (control) or 1μM AZD8055 treatments at 5dpr and 20dpr. Inhibition of TOR signalling using AZD8055 had no effects on mean rosette density (number of rosettes per 1000 large cells) or mean cell areas of larger cells. Box plots show median values (black full lines), the first to third interquartile ranges (box), whiskers indicate 1.5x IQR.

Single data points represent mean rosette density per animal. For rosette density analysis, normality was assessed using Shapiro–Wilk tests, control versus treatment comparisons were performed using two-sided Wilcoxon rank-sum tests and followed by linear mixed-effects models (LMMs). In violin plots, means are represented by black full lines and medians by red dashed lines. Data points represent means of large cell area per animal. Cell density comparisons were assessed using LMMs on log-transformed mean cell area values. Significance codes for model adjusted P-values: ns = non-significant. Abbreviations: dpr: days post-refeeding, AZD: AZD8055. Scale bars: 20µm. For both datasets, pairwise comparisons of estimated marginal means (EMMs) were Tukey-adjusted (see Supplementary Data 7, 8).

Supplementary Tables 1-2

Supplementary Table 1. Key resources table including reagent catalogue numbers and identifiers

REAGENT or RESOURCE	SOURCE	IDENTIFIER
**Antibodies**		
Cleaved Caspase Substrate Motif [DE(T/S/A)D] MultiMab®	Cell Signaling Technology	Cat# 8698
Phospho-p38 MAPK (Thr180/Tyr182) (D3F9) XP® (Alexa Fluor® 594 Conjugate)	Cell Signaling Technology	Cat# 8632S
Anti-Phospho-S6 Ribosomal Protein (Ser235/236)	Cell Signaling Technology	Cat# 4858
Phospho-p44/42 MAPK (Erk1/2) (Thr202/Tyr204) (D13.14.4E) XP®	Cell Signaling Technology	Cat# 4370T
Phospho-p38 MAPK (Thr180/Tyr182) (D3F9) XP®	Cell Signaling Technology	Cat# 4511T
Nv_Cadherin1	Courtesey of Uli Technau Lab	N/A
Goat anti-Rabbit IgG (H+L) DyLight 488 conjugate	LifeTech	Cat# 35552
Goat anti-Rat IgG (H+L), Alexa Fluor® 633 conjugate	LifeTech	Cat# A21094
**Other reagents**		
AZD8055	Nordic Biosite	Cat# hy-10422
Staurosporine	Sigma Aldrich	Cat# S4400
U0126	Merck	Cat# 662005
QV-D-OPh	MedChemExpress	Cat# hy-12305
TrueCut Cas9 protein v2	ThermoFisher	A36498
**Deposited data**		
Nematostella vectensis genome	https://mycocosm.jgi.doe.gov/Nemve1/Nemve1.home.html	N/A
**Experimental models: Organisms/strains**		

Nematostella vectensis	Michael Sars Centre (Rhode River MD strain)	N/A
Nematostella vectensis: β-Catenin-mOrange2 fusion protein transgenic knock-in line	This study	N/A
**Oligonucleotides**		
Primers for gRNA oligos and primers for PCR and sequencing of the transgenic line can be found in Supplementary Table 2	This study	N/A
**Recombinant DNA**		
Plasmid containing β-Catenin-mOrange2 donor for HDR-mediated knock-in in pJet1.2 backbone.	This study	N/A
**Software and algorithms**		
Primer3Plus	http://www.bioinformatics.nl/cgi-bin/primer3plus/primer3plus.cgi	N/A
CRISPOR	http://crispor.tefor.net/	N/A
Fiji	https://imagej.net/software/fiji/	N/A
Cellpose	https://www.cellpose.org	N/A
Napari	https://napari.org/dev/index.html	N/A
R Studio	R Core Team 2025	v.4.5.2 https://www.r-project.org/

Supplementary Table 2. List of resources for transgenic line generation, including primer sequences, single guide RNAs, homology arms, mOrange2 sequence.
Table containing list of single guide RNAs, corresponding melt-curve analysis primers and mutant genotyping primers for the generation of β-catenin-mOrange4 knock-in line (see also Supplementary Fig. 1B)

Name	Purpose	Sequence
βcatenin_exon8	genotyping reverse primer (transcript)	GCTGGTCAACTCTACGGACAGCCG
βcatenin_intron9	genotyping reverse primer (genomic)	GAAAGCCGCGTCCGACGTCAC
mOrange2_forward	genotyping primer (transcript/genomic)	CtGTCTGAAAGCCCTCGTAtGGtC
mOrange2_reverse	genotyping primer (transcript/genomic)	GCTGCACtGGCTTCTTtGCCTTG
βcatenin_UTR4	genotyping forward primer (transcript/genomic)	GGAGAGTGTATGCTACTCAACCCGAAGC
βcat-Ct-g161-guideRNA	CRIPSR/Cas9 guideRNA	GAAATTAATACGACTCACTATAGCAGAGGTCTGTGTCATACAGGTTTTAGAGCTAGAAATAGCAAG
βcat-HA1-FG	Homology Arm 1 forward primer	GCTCGAGTTTTTCAGCAAGATgCTAGCGCCGCCGCCACAAAAATTCTAGC
βcat-HA1mut_lnk-RG	Homology Arm 1 reverse primer	CACCCGATCCGCCTCCACCGAGGTCgGTaTCATAtAaAGGACCCCCTGGATTTTGGTAGTG
βcat-link-mOr2-FG	(GGGGS)2 linker	GACCTCGGTGGAGGCGGATCGGGTGGAGGCGGGTCGGTGAGCAAGGGCGAGGAGAATAACATGG
βCat-Ct-qP-II-F	CRISPR/Cas9 guide RNA forward primer	CGGGAGGACACTACCAAAATCC
Invariant T7 promotor/tracrRNA	Invariant reverse primer	AAAAGCACCGACTCGGTGCCACTTTTTCAAGTTGATAACGGACTAGCCTTATTTTAACTTGCTATTTCTAGCTCTAAAAC
βcat-HA2-F	Homology Arm 2 forward primer	TGAataatccacacccccctttggc
βcat-HA2-RGc	Homology Arm 2 reverse primer	TGTAGGAGATCTTCTAGAAAGATGCGATCGCcctcgacagatcatgcaatcgtgtcc
mOrange2 full sequence		GTGAGCAAGGGCGAGGAGAATAACATGGCaATCATCAAGGAGTTCATGaGaTTCAAGGTGaGaATG

		GAGGGCTCaGTGAACGGCCAC<br>GAGTTCGAGATCGAGGGCGAG<br>GGCGAGGGCaGaCCaTACGAGG<br>GCTTTCAGACaGCaAAGCTGAA<br>GGTGACaAAGGGTGGCCCaCTG<br>CCaTTCGCaTGGGACATCCTGT<br>CCCCaCATTTCACaTACGGCTCa<br>AAGGCaTACGTGAAGCACCCaG<br>CaGACATCCCaGACTACTTCAAG<br>CTGTCaTTCCCaGAGGGCTTCA<br>AGTGGGAGaGaGTGATGAACTA<br>CGAGGACGGCGGCGTGGTGAC<br>aGTGACaCAGGACTCaTCaCTcC<br>AaGACGGCGAGTTCATCTACAA<br>GGTGAAGCTGaGaGGCACaAAC<br>TTCCCaTCaGACGGCCCaGTgAT<br>GCAGAAGAAGACaATGGGCTGG<br>GAGGCaTCaTCaGAGaGaATGTA<br>CCCaGAGGACGGCGCaCTGAAG<br>GGCAAGATCAAGATGAGaCTGA<br>AGCTGAAGGACGGCGGCCACT<br>ACACaTCaGAGGTgAAGACaACa<br>TACAAGGCaAAGAAGCCaGTGC<br>AGCTGCCCGGCGCaTACATCGT<br>gGACATCAAGcTGGACATCACaT<br>CaCACAACGAGGACTACACaAT<br>CGTGGAACAGTACGAAaGaGCa<br>GAGGGCaGaCACTCaACaGGCG<br>GCATGGACGAGCTGTACAAG

Supplementary Notes

Rosette Detection Pipeline

Deep Learning Model Performance

To validate the rosette detection model, we evaluated its performance on a held-out test set comprising 20% of the annotated data. The model achieved a Dice coefficient (F1-score) of 0.54 at the image level and 0.61 at the patch level. While exact boundary delineation remained challenging due to biological variability, the model demonstrated a Recall of 0.65, indicating robust sensitivity for structure localisation. Nonetheless, pixel-level metrics penalise boundary imprecision: a rosette that is correctly localised, but whose predicted area only partially overlaps the ground truth contributes false-negative pixels without constituting a missed detection event. Because we validate the predictions manually, the important measure is whether rosettes are indicated by the model to speed up this process. Hence, we performed an event-based evaluation where we detect 82.9% of rosettes (223/269) with ≥10% coverage above a threshold of 0.4 (clearly visible to the human reviewer). Of the missed instances, the majority retained sub-threshold but visually detectable heatmap signal; see Supplementary Notes Table 1 for summary metrics and Supplementary Notes Table 2 for the full pixel-level coverage distribution at threshold 0.5.

Visual inspection confirmed that while the model effectively identified the core regions of rosette structures, it occasionally over-estimated boundaries or flagged dense cell aggregates or badly segmented areas (false positives). However, this tendency to prioritise recall (sensitivity) over precision was advantageous for the downstream workflow, as false positive candidates are rapidly dismissible by expert annotators, whereas false negatives would result in laborious additional annotations.

Automated Detection and Pipeline Efficiency

To ensure the rigorous quantification of rosette formation across images, we integrated rosette inferences into a fully automated pipeline. By utilising model predictions as “candidate proposals” rather than final ground truth, we implemented a human-in-the-loop workflow that standardised Regions of Interest (ROIs) and reduced observer fatigue. Consequently, the model-assisted curation increased rosette annotation speed by approximately 2.35-fold (<1 min/image) compared to naive manual annotation on segmented masks (~2 min/image), which was already improved from a completely manual annotation on raw images (~4 min/image). When combined

with the upstream automated cell segmentation, the integrated pipeline greatly accelerated the total quantification process by ~5-fold while ensuring expert-verified accuracy.

Image Acquisition and Preprocessing

Confocal image acquisition was performed using an Olympus FV3000 confocal microscope controlled by Olympus FV3000 control software (version 2.4.1.198). Images were acquired using a 60× silicone-immersion objective (UPLSAPO 60XS2, NA 1.3). The image size was 1024 × 1024 pixels with an effective pixel size of 0.134μm (zoom 1.55). Z-stacks consisted of an average range of 5-15 optical sections at 1μm step size. Fluorescence excitation was performed using the 561 nm laser (transmissivity 6.77%) detecting Alexa Fluor 532 (i.e.: mOrange2) on an HSD1 detector (gain 1.0, 567 V). The pinhole was set to 257μm. Images were acquired in line-sequential scanning mode with 2× frame averaging. Z-stacks were acquired for each field of view. To generate 2D representations for analysis, Maximum Intensity Projections (MIPs) were generated from the relevant Z-slices that produced the most completed visualisation of the epidermal layer. These projections are hereafter referred to as "images," with each representing a single field of view (available online at Zenodo data repository: 10.5281/zenodo.19726756) .

Automated Cell Segmentation

To extract the cellular topology necessary for rosette identification, we established a robust segmentation pipeline combining deep learning with topological curation.

Iterative Model Training (Human-in-the-Loop):

We utilised Cellpose[1–3] to segment cell membranes. To optimise performance for our specific tissue architecture, we employed an iterative human-in-the-loop fine-tuning strategy:

1. Initial Inference: Baseline segmentation was performed using the pre-trained cyto3 model.
2. Correction: Segmentation errors were manually corrected using the Cellpose GUI.
3. Fine-Tuning: The cyto3 model was retrained on the corrected dataset to improve subsequent inference.

4. Iteration: This cycle (Inference → Correction → Retraining) was repeated sequentially across 168 images until the model achieved consistent, high-fidelity segmentation of all cell membranes (Flow threshold: 0.4; Diameter: Auto-detect).

Post-Processing and Topological Curation Raw segmentation outputs often contain topological artifacts (e.g., intercellular gaps, edge distortions) that can distort downstream analysis. We developed a custom post-processing module to enforce tissue contiguity:

- Morphological Gap Closing: Small background regions (false positive intercellular gaps) were merged into the largest neighbouring cell to ensure a continuous tissue layer.
- Recovery of Missed Cells: Large unsegmented regions within the tissue proper (area > 50 pixels) were detected and instantiated as new cell identities to correct false negatives.
- Topological Filtering: "Isolated" segments not sharing boundaries with neighbouring cells (typically debris or out of focus regions) were removed.
- Edge Artifact Handling: Cells intersecting the field-of-view boundaries were excluded to prevent the analysis of incomplete or artificially truncated cell geometries.
- The final output consisted of curated, contiguous segmentation masks where each cell possesses a unique integer ID.

Deep Learning Model Architecture and Training

Network Architecture

The model utilises a 4-level encoder-decoder depth with additive Attention Gates applied at the three upsampling junctions. Feature maps increase from 64 filters (Level 1) to 1024 filters (Bottleneck). The input is a 2-channel tensor (512 × 512 × 2) consisting of:

- Cell Boundaries: Binary map with boundaries dilated to increase feature visibility.
- Cell Masks: Binary map with thin inner cell boundary lines.

The output layer utilises a 1 × 1 convolution with a Sigmoid activation function to generate pixel-wise probabilities.

Parameter	Value
Loss Function	Combined Loss (0.5× BCE +0.5× Dice)
Optimiser	AdamW
Learning Rate	1 × 10−4
Batch Size	4
Early Stopping	Patience of 15 epochs (monitoring validation loss)

Supplementary Notes Table 1. Training Hyperparameters ("Champion" Settings).

Data Augmentation

To prevent overfitting on geometric shapes, a "hard" augmentation regime was applied during training using the Albumentations library:

- Random Rotation: p = 0.5
- Elastic Deformation: α = 120, σ = 6, p = 0.4
- Affine Transforms: Scale 0.9–1.1x, Shift ±10%, p = 0.6
- Coarse Dropout: Simulating segmentation errors (p = 0.3)

Ground Truth Generation for Rosette Detection

With high-quality cell segmentations established, we generated a ground truth dataset to train the downstream rosette detection model. Annotation Protocol Curated segmentation masks for 268 samples (available online at Zenodo data repository: 10.5281/zenodo.19726756) were loaded into napari[4] and overlaid with the original fluorescence images to provide biological context. Rosette cells were manually annotated based on the following criteria: Structural Organization: A group of minimum 5 cells that share a common central axis or that coalesce into a round central and small cell (extruding cell). Annotated cells were assigned a unique label per rosette, creating a labelled mask of "Rosette vs. Background" used to train the Deep Learning model.

Rosette Detection Model

To automate the identification of rosettes, we implemented a deep learning approach based on Convolutional Neural Networks (CNNs)[5]. Specifically, we utilised the Attention U-Net architecture[6], a modification of the standard U-Net designed to enhance sensitivity to local features.

Architecture and Inputs Unlike standard CNNs, the Attention U-Net employs additive attention gates within its skip connections. This mechanism allows the model to selectively weight features passed from the encoder to the decoder, effectively learning to suppress irrelevant background regions while highlighting the specific multicellular organizations characteristic of rosettes.

The model was trained exclusively on geometric inputs, specifically, binary cell boundary maps and morphologically dilated cell boundaries derived from segmentation data, without access to raw fluorescence intensity channels.

Model performance was evaluated on a held-out validation set (20% of the total dataset, 54 images, 269 rosette instances; random split, seed = 42) using the Dice coefficient and F1-score to ensure robust generalisation.

**Metric**	**Value**
Pixel-level Dice	0.54
Pixel-level F1	0.60
Pixel-level Recall	0.65
Event-level recall (≥1 predicted pixel, threshold 0.5)	88.8% (239/269)
Rosettes with ≥10% pixel coverage at threshold 0.4	82.9% (223/269)
Completely missed (zero heatmap signal)	11.2% (30/269)

Supplementary Notes Table 2. Rosette detection model performance on held-out validation set. Pixel-level metrics treat each pixel independently and penalise boundary imprecision. Event-level recall counts a rosette as detected if ≥1 predicted pixel falls within its ground-truth mask (threshold 0.5). Coverage-visibility reports the fraction of rosettes with ≥10% of their pixels predicted positive at threshold 0.4, corresponding to clearly visible heatmap signal for the human reviewer. Validation set: 54 images, 269 rosette instances, random split (seed = 42).

Coverage	N. rosettes	%	Cumulative %
0% — no predicted pixels	30	11.2%	11.2%
0–20% (detected, weak signal)	32	11.9%	23.1%
20–40%	12	4.5%	27.5%
40–60%	15	5.6%	33.1%
60–80%	53	19.7%	52.8%
80–100%	127	47.2%	100.0%

Supplementary Notes Table 3. Distribution of pixel-level model coverage per ground-truth rosette instance. For each of the 269 rosettes in the held-out validation set, the fraction of constituent pixels predicted positive at threshold 0.5 was computed. Rosettes in the 0% bin were completely absent from the prediction heatmap; all other bins represent detected rosettes with varying degrees of spatial coverage. The majority of rosettes (88.8%) received at least partial prediction signal, and 47.2% had >80% of their area covered.

The expert annotator reviewed the continuous prediction heatmap overlaid on the full cell segmentation mask in napari, with all cell boundaries visible across the entire image (i.e. not only regions flagged by the model). This ensured that rosettes absent from the model's predictions could be identified and added during review. This semi-automated workflow combines the scalability of deep learning with human expertise, correcting false positives/negatives and refining boundaries to generate a curated high-quality dataset for downstream analysis.

Computational Pipeline and Reproducibility

All data preprocessing, model training and evaluation steps were orchestrated using Snakemake pipeline[7,8] to ensure consistent execution and automated dependency tracking. Software environments were managed using Poetry to strictly pin package versions (e.g., PyTorch, Albumentations) for reproducibility[9]. The complete source code and automated analysis pipeline (Fig. 1) are available at https://doi.org/10.5281/zenodo.20479352 under MIT License.

Quantification and Statistical Analysis

Metric Definitions following the curation of rosette masks, quantitative metrics were extracted using custom Python scripts.

- Rosette Density: Defined as the number of validated rosettes per normalised tissue area (e.g., rosettes per 1000 segmented cells).
- Rosette Valency: Defined as the number of distinct cell identities constituting a single rosette.
- Cell area: Square pixel/cell (0.1337μm/px) extracted with scikit-image library[10].

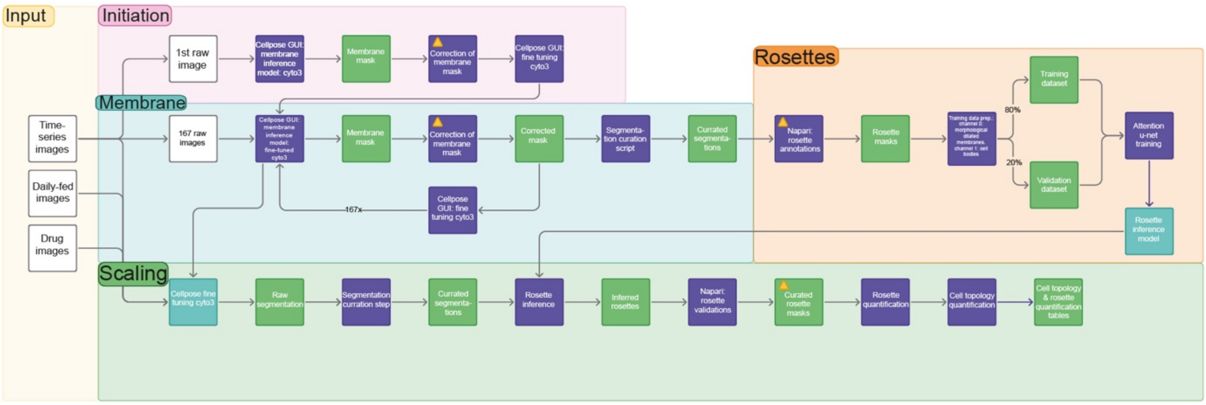

Supplementary Notes Fig. 1. Image segmentation and rosette ID bioinformatic pipeline.

Computational hardware and resources

All model training and inference was performed on an Apple MacBook Pro (M2 pro, 64GB unified memory) using the PyTorch MPS backend (Apple Silicon GPU acceleration). Training converged within a few hours on the M2 MacBook Pro. Inference time per image is negligible (<1 minute). Carbon footprint is minimal given the hardware and training duration.

- Model available on Hugging Face: https://huggingface.co/NoahBruderer/nematostella_rosette_detection
- Custom code script available on GitHub: https://doi.org/10.5281/zenodo.20479352
- Training data set available on Zenodo: https://doi.org/10.5281/zenodo.19726756

Cell classification via Gaussian Mixture Models (GMM)

All statistical analyses and graphical representations of data were performed using R (v. 4.5.2 (2025-10-31), https://www.r-project.org/[11]) and the following packages: `mclust` (v. 6.1.2[12]), `ggplot2` (v. 4.0.0[13]), `dplyr` (v. 1.1.4[14]), `tidyr` (v. 1.3.1[15]), `lme4` (v. 1.1.37[16]), `lmerTest` (v. 3.1.3[17], using Type III ANOVA, Satterthwaite's approximation), and `emmeans` (v. 2.0.0[18]). All statistical tests were two-sided. To categorise the bimodal populations of epidermal cells in *Nematostella vectensis*

juveniles, cell areas were analysed using Gaussian Mixture Models (GMMs). Bimodality reflects two consistent populations: small cells, which remain stable in size and proportion across conditions, and large cells, which are dynamic and of interest for rosette formation and cell area analysis.

Data preprocessing

Raw data output tables provided in a public data repository (Zenodo: 10.5281/zenodo.19726756, raw data files 1, 3, 5-8). Cell areas were acquired from confocal image stacks and converted from pixel units (pixels²) to physical units (µm²) using a scaling factor of 1 px² = 0.01786µm². To ensure biological boundaries, a hard upper limit of 100µm² was applied to exclude abnormally large cells. Extreme outliers were further removed on a per-image basis by filtering out the top 0.01% and bottom 0.01% of cell area values. Images with fewer than 10 remaining cell measurements after filtering were excluded from subsequent Gaussian mixture model (GMM) fitting (see Supplementary Notes Table 4).

GMM fitting

For each image, log-transformed cell areas (log1p(area)) were modelled using a two-component Gaussian mixture model fitted via the expectation–maximisation (EM) algorithm, as implemented in the `mclust` R package (model "E", assuming equal variance between components). Posterior probabilities were computed for each cell to quantify classification confidence. The boundary between *Small* and *Large* cells was defined as the midpoint between the back-transformed means of the two Gaussian components. Cells with areas below this threshold were classified as *Small*, whereas those above were classified as *Large*.

Outputs and reproducibility

The final output for each image included each cell's area, log-transformed area, GMM-based classification and posterior probability (see public data repository 10.5281/zenodo.19726756, cell-level data files 2, 4, and 9–12). All downstream analyses, including rosette density and cell area dynamics, were performed using these classified populations, with a primary focus on the *Large* cell subset. The procedure was implemented in R using the `mclust` (v. 6.1.2[12]) and `tidyr` (v. 1.3.1[15]) packages. Reproducibility was ensured by setting a fixed random seed (e.g., `set.seed(123)`)

Dataset	Total cells	Cells removed (outliers + upper limit)	% Cells removed
Timeseries	432,695	9,044	2.09%
Baseline	156,999	3,288	2.10%
AZD8055	204,107	4,246	2.08%
U0126	227,964	6,761	2.97%
Q-VD-OPh	244,087	5,065	2.07%
Staurosporine	228,531	5,479	2.40%

Supplementary Notes Table 4. Cells removed in each dataset following filtering steps to detect outliers and segmentation errors.

Cells removed include those filtered by the 100µm² upper limit and the 0.01% top/bottom quantile filtering. All subsequent analyses of rosettes and cell area used the remaining cells classified via GMM.

Linear mixed model fitting for rosette density and cell area analysis

Across all datasets, all statistical tests were two-sided, timepoint and treatment condition were included as fixed effects; replicate and animal identity were included as random effects. Effect sizes are represented by differences in estimated marginal means with associated 95% confidence intervals.

Starvation dataset

For each image, quantified the number of rosettes and total number of cells was quantified, then proportion was calculated using number of rosettes per 1,000 large cells per image, and then averaged per animal. Individual cell areas were measured from segmented images and classified as Large or Small using GMM-based clustering; mean cell area per animal was calculated. Linear mixed-effects models (LMMs) were fitted with packages `lme4` (v. 1.1.37[16]) and `lmerTest` (v. 3.1.3[17]. Significance of fixed effects was assessed using ANOVA. The resulting LLMs were used:

- Rosette density:
  - `rosettes per 1000 large cells ~ timepoint + (1 | replicate/animal)`
- Cell area:
  - `log(mean cell area) ~ timepoint + (1 | animal)`

Here `timepoint` was treated as a fixed effect; animal identity nested within replicate was included as a random intercept to account for repeated measures and hierarchical structure. LMMs account for repeated measures and hierarchical structure where random effects capture variation among replicates and animals.

Random effects SD	Residual SD	Fixed effect (Timepoint)	Notes
Animal: 1.513; Replicate: 1.528	7.899	$F_{(8,\ 481.8)}$ = 8.31, p = 1.382 × 10^{-10}	Significant variation over time; residuals −1.929 to 3.511

Supplementary Notes Table 5. Assessing the results of the linear models fitted on the rosette density analysis from the starvation dataset via analysis of variance (ANOVA). Two-sided linear mixed-effects models (LMM) with Tukey-adjusted EMM pairwise comparisons were used for data analysis. Corresponding to data in Fig. 2B.

Cell class	Random effects SD	Residual SD	Fixed effect (Timepoint)	Notes
Large	0.049	0.166	$F_{8,229}$ = 8.29, p = 7.335 × 10^{-10}	Temporal changes captured; model fit reasonable
Small	0.021	0.129	$F_{8,232}$ = 1.66, p = 0.108	No significant temporal variation

Supplementary Notes Table 6. Assessing the results of the linear mixed-effects models (LMMs) fitted on the cell area analysis (large and small cell subsets, respectively) from the starvation dataset via Type III ANOVA with Satterthwaite approximation for denominator degrees of freedom. All tests were two-sided. Pairwise comparisons of estimated marginal means (EMMs) were Tukey-adjusted. Corresponding to data in Fig. 2F-H.

No significant batch effects were observed (variance component for replicate similar to animal-level variance), and residual diagnostics showed no major deviations from assumptions. Estimated marginal means were computed per timepoint using emmeans (R package `emmeans` (v. 2.0.0[18]), with degrees of freedom estimated using the Kenward–Roger approximation. Tukey-adjusted pairwise comparisons were used to assess differences between timepoints. Rosette data shows random effects that are small relative to residual variance, indicating robustness across individuals and replicates. Cell area analysis shows that large cells show significant time-dependent changes while small cells remain stable. Minor contribution of random effects indicates that individual differences do not drive observed trends. After model fitting, Estimated

Marginal Means (EMMs) and pairwise Tukey-adjusted comparisons were used to summarise model effects and for reporting intext (see Supplementary Data 5, 6).

Daily fed dataset

Identical approach as in starvation dataset, in which rosette density per 1,000 large cells is calculated and cell area analysis is split between large and small. Mean cell area was log-transformed to satisfy normality assumptions in the models. The following LMMs were used:

- Rosette density:
  - `rosettes per 1000 large cells ~ timepoint + (1 | replicate)`
- Cell area:
  - `log(mean cell area) ~ timepoint + (1 | replicate)`

Random effects SD	Residual SD	Fixed effect (Timepoint)	Notes
Replicate: 0	5.39	$F_{2,177}$ = 1.32, p = 0.27	Temporal effect not significant; replicate variability negligible

Supplementary Notes Table 7. Assessing the results of the linear models fitted on the rosette density analysis from the daily fed dataset via analysis of variance (ANOVA). Two-sided linear mixed-effects models (LMM) with Tukey-adjusted EMM pairwise comparisons were used for data analysis. Corresponding to data in Fig. 2A.

Cell Type	Random effects SD	Residual SD	Fixed effect (Timepoint)	Notes
Large	0.041	0.173	$F_{2,5.9}$ = 0.16, p = 0.85	No significant temporal variation
Small	0.040	0.136	$F_{2,4.7}$ = 0.085, p = 0.92	No significant temporal variation

Supplementary Notes Table 8. Assessing the results of the linear models fitted on the cell area analysis (large and small cell area subsets, respectively) from the daily fed dataset via analysis of variance (ANOVA). Two-sided linear mixed-effects models (LMM) with Tukey-adjusted EMM pairwise comparisons were used for data analysis. Corresponding to data in Fig. 2C-E.

Minor random effects indicate negligible between-replicate variation. Both large and small cell remain stable across timepoints. After model fitting EMMs and pairwise

Tukey-adjusted comparisons were used to summarize model effects and for reporting intext (see Supplementary Data 2-4).

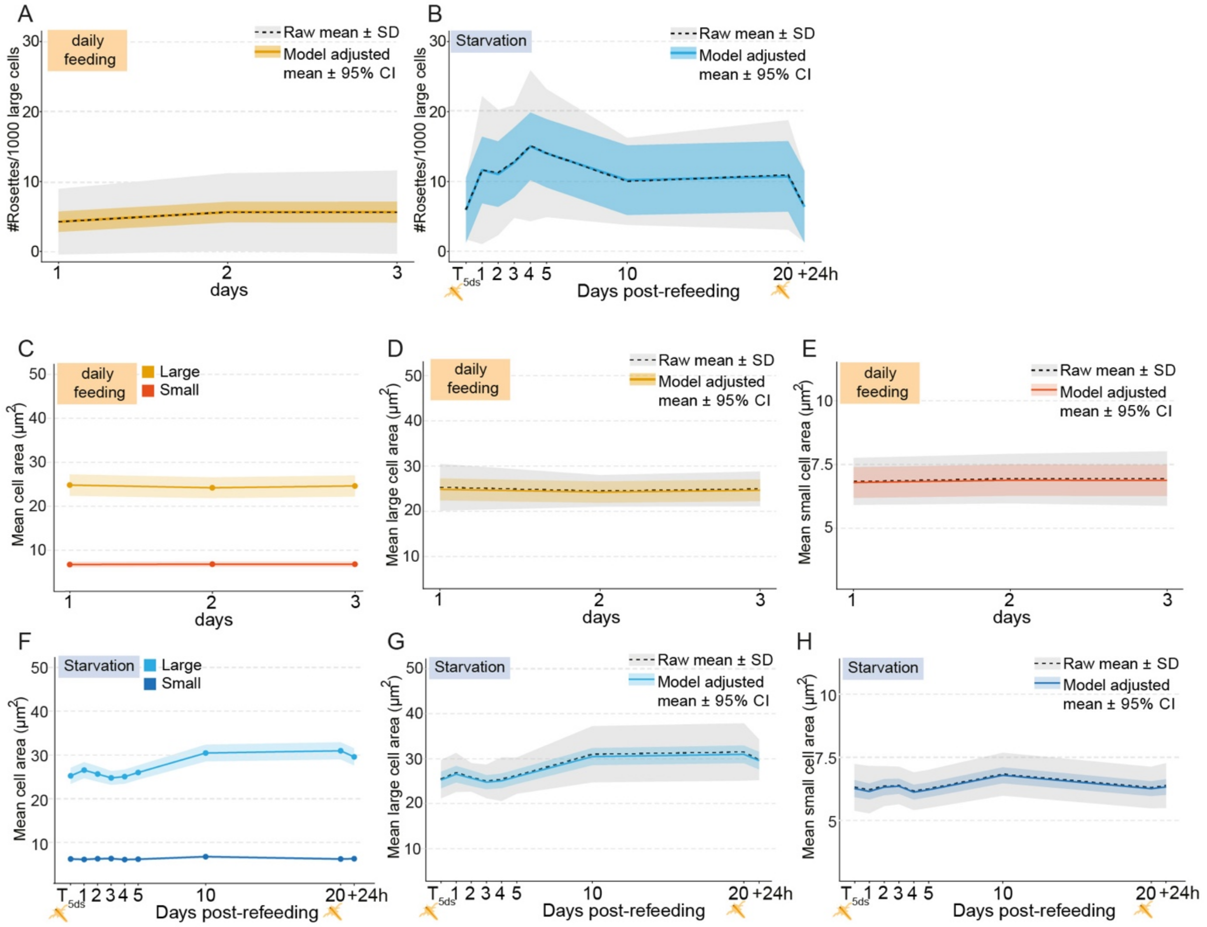

Supplementary Notes Fig. 2. Supplementary statistical modelling graphs. (A) Daily fed dataset - Mean rosette density: raw mean + SD vs model-adjusted mean + 95% CI. (B) Starvation dataset – Mean rosette density: raw mean + SD vs model-adjusted mean + 95% CI. (C) Daily fed dataset: Model-adjusted means cell area (+ 95% CI) for large and small cell populations separately. (D) Daily fed dataset: Raw large cell area mean + SD vs model-adjusted mean + 95% CI. (E) Daily fed dataset: Raw small cell area mean + SD vs model-adjusted mean + 95% CI. (F) Starvation dataset: Model-adjusted means cell area (+ 95% CI) for large and small cell populations separately. (G) Starvation dataset: Raw large cell area mean + SD vs model-adjusted mean + 95% CI. (H) Starvation dataset: Raw small cell area mean + SD vs model-adjusted mean + 95% CI. Abbreviations: SD: standard deviation, CI: confidence interval, ds: days starved. See also Supplementary Data 2, 3, 5, 6.

Drug dataset

Rosette density was obtained following the same methods as for the timeseries datasets, normalised as rosettes per 1,000 large cells per animal. Cell area was measured per animal and classified as Large or Small based on GMM classification

(see cell-level tables in the raw data files 9-12 found in public data repository: 10.5281/zenodo.19726756). Mean cell area per class was computed. In the rosette density data, because distributions were non-normal (verified with Shapiro–Wilk tests), control vs treatment comparisons for each timepoint (5 and 20 dpr) Wilcoxon rank-sum significance tests were used, and replicate effects were assessed using Kruskal–Wallis tests. Linear mixed-effects models (LMMs) were fitted for both rosette density and log-transformed mean cell area to account for hierarchical/nested structure. LMMs were used as the primary inferential framework to account for nested structure, while non-parametric tests were used as robustness checks for control–treatment comparisons. Cell area models were fitted separately per timepoint to avoid overparameterization and because treatment effects were assessed independently at each stage. Because log-transformation produced approximately normal residuals for cell area models, inference was based on the LMM framework without additional non-parametric testing.

- Rosette density:
  - `rosettes per 1000 large cells ~ Condition*timepoint + (1 | replicate)`
- Cell area:
  - `log(mean cell area)~ Condition + (1 | replicate)`

Treatment	Timepoint	Normality (Shapiro p)	Wilcoxon (W, p)	Kruskal–Wallis Replicate effect (χ^2, df, p)	Random effects SD (Rep / Res)	Notes
Staurosporine	T5	Control: 0.0076; Treated: 1.94×10^{-5}	W = 447, p = 0.994	χ^2 = 29.5, df = 2, p = 3.86×10^{-7}	Rep: 2.471; Res: 4.457	Strong replicate effect; no treatment effect
	T20	Control: 0.154; Treated: 1.34×10^{-5}	W = 490, p = 0.404	χ^2 = 10.2, df = 2, p = 0.0062	Rep: 2.471; Res: 4.457	Moderate replicate effect; no treatment effect
AZD8055	T5	Control: 9.89×10^{-4}; Treated: 1.98×10^{-4}	W = 463, p = 0.643	χ^2 = 19.3, df = 2, p = 6.40×10^{-5}	Rep: 1.586; Res: 4.849	Strong replicate effect; no treatment effect
	T20	Control: 0.0536;	W = 402, p = 0.277	χ^2 = 0.080, df	Rep: 1.586;	No replicate or

		Treated: 0.0741		= 2, p = 0.961	Res: 4.849	treatment effect
U0126	T5	Control: 3.04×10^{-3}; Treated: 7.60×10^{-4}	W = 226, p = 0.0023	χ^2 = 16.0, df = 2, p = 3.38×10^{-4}	Rep: 1.266; Res: 4.587	Significant treatment effect; replicate effect present
	T20	Control: 2.66×10^{-5}; Treated: 1.24×10^{-5}	W = 448, p = 0.160	χ^2 = 2.95, df = 2, p = 0.229	Rep: 1.266; Res: 4.587	No treatment or replicate effect
Q-VD-OPh	T5	Control: 0.00219; Treated: 0.136	W = 538, p = 0.433	χ^2 = 0.993, df = 2, p = 0.609	Rep: 0.608; Res: 3.269	No treatment or replicate effect
	T20	Control: 0.0318; Treated: 0.0622	W = 615, p = 0.0546	χ^2 = 6.27, df = 2, p = 0.0435	Rep: 0.608; Res: 3.269	Weak treatment trend; replicate effect present

Supplementary Notes Table 9. Statistical analysis of rosette density in the drug dataset for each treatment condition and timepoint. Normality was assessed using Shapiro–Wilk tests; control versus treatment comparisons were performed using two-sided Wilcoxon rank-sum tests; replicate effects were assessed using Kruskal–Wallis tests. Linear mixed-effects model (LMM) pairwise comparisons used Tukey-adjusted estimated marginal means (EMMs). Resulting data found in Supplementary Data 7, 9, 11, 13.

Treatment	Effect	NumDF	DenDF	F value	p-value	Notes
Staurosporine	Condition	1	113.10	0.83	0.365	No treatment effect
	Timepoint	1	113.09	0.012	0.914	No temporal effect
	Condition × Timepoint	1	113.06	0.92	0.339	No interaction
AZD8055	Condition	1	115.28	0.033	0.855	No treatment effect
	Timepoint	1	115.24	4.01	0.0475	Significant temporal change
	Condition × Timepoint	1	115.17	0.598	0.441	No interaction
U0126	Condition	1	121.46	7.07	0.0089	Significant treatment effect
	Timepoint	1	120.67	3.08	0.0816	Trend only
	Condition × Timepoint	1	120.93	3.62	0.0596	Borderline interaction

Q-VD-OPh	Condition	1	119.50	7.06	0.00894	Significant treatment effect
	Timepoint	1	119.29	0.165	0.686	No time effect
	Condition × Timepoint	1	119.36	0.501	0.480	No interaction

Supplementary Notes Table 10. Linear mixed-effects model (LMM) analysis of rosette density in the drug dataset. Rosette density (rosettes per 1,000 large cells) was modelled as a function of treatment condition, timepoint, and their interaction, with replicate included as a random effect. Fixed effects were assessed using Type III ANOVA with Satterthwaite's approximation for degrees of freedom. All statistical tests were two-sided. Post hoc pairwise comparisons of estimated marginal means (EMMs) were performed using Tukey adjustment for multiple comparisons.

Treatment	Timepoint	Cell Type	Replicate SD	Residual SD	Fixed effect (Condition)	Notes
Staurosporine	T5	Large	0.112	0.235	$F_{1,56}$ = 6.61, p = 0.0128	Significant decrease in treated
		Small	0.107	0.127	$F_{1,56}$ = 2.04, p = 0.158	No significant effect
	T20	Large	0.203	0.168	$F_{1,55}$ = 27.62, p = 2.48×10^{-6}	Strong decrease in treated
		Small	0.069	0.132	$F_{1,55}$ = 2.93, p = 0.093	Weak/non-significant trend
AZD8055	T5	Large	0.060	0.192	$F_{1,55}$ = 0.36, p = 0.551	No effect
		Small	0.109	0.137	$F_{1,55}$ = 1.78, p = 0.188	No effect
	T20	Large	0.139	0.215	$F_{1,58}$ = 2.84, p = 0.097	Weak trend (decrease)
		Small	0.013	0.127	$F_{1,58}$ = 0.12, p = 0.728	No effect
U0126	T5	Large	0.000	0.210	$F_{1,57}$ = 0.28, p = 0.597	No effect
		Small	0.000	0.133	$F_{1,57}$ = 3.74, p = 0.058	Weak trend (increase)
	T20	Large	0.121	0.188	$F_{1,63}$ = 1.44, p = 0.234	No effect

		Small	0.000	0.124	$F_{1,65}$ = 0.42, p = 0.517	No effect
Q-VD-OPh	T5	Large	0.000	0.184	$F_{1,61}$ = 0.0013, p = 0.972	No effect
		Small	0.000	0.118	$F_{1,61}$ = 0.0034, p = 0.954	No effect
	T20	Large	0.000	0.252	$F_{1,60}$ = 0.021, p = 0.885	No effect
		Small	0.000	0.147	$F_{1,60}$ = 0.0014, p = 0.971	No effect

Supplementary Notes Table 11. Linear mixed-effects model (LMM) analysis of cell area in the drug dataset. Log-transformed mean cell area was modelled separately for large and small cell populations at each timepoint, with treatment condition as a fixed effect and replicate included as a random effect. Fixed effects were assessed using Type III ANOVA with Satterthwaite's approximation for degrees of freedom. All statistical tests were two-sided. Post hoc pairwise comparisons of estimated marginal means (EMMs) were performed using Tukey adjustment for multiple comparisons.

For all inhibitors, LMMs accounted for replicate-level variability; in most cases, this variance was negligible. LMM contrasts were broadly consistent with non-parametric tests, confirming that replicate variability did not substantially alter significance of comparisons. Log-transformation of mean area ensured residuals approximated normality. Type III ANOVA with Satterthwaite approximation; Kenward–Roger used for EMMs. In several models, replicate-level variance was estimated as ~0 (singular fit), indicating negligible batch effects. After model fitting, EMMs and pairwise Tukey-adjusted comparisons were used to summarize model effects and for reporting intext. Pairwise comparisons from EMMs were consistent with model assumptions.

Supplementary References

1. Stringer, C., Wang, T., Michaelos, M. & Pachitariu, M. Cellpose: a generalist algorithm for cellular segmentation. *Nat. Methods* **18**, 100–106 (2021).
2. Pachitariu, M. & Stringer, C. Cellpose 2.0: how to train your own model. *Nat. Methods* **19**, 1634–1641 (2022).
3. Stringer, C. & Pachitariu, M. Cellpose3: one-click image restoration for improved cellular segmentation. *Nat. Methods* **22**, 592–599 (2025).
4. Sofroniew, N. *et al.* napari: a multi-dimensional image viewer for Python. *Zenodo* (2022). doi:10.5281/zenodo.3555620
5. LeCun, Y., Bengio, Y. & Hinton, G. Deep learning. *nature* **521**, 436–444 (2015).
6. Oktay, O. *et al.* Attention u-net: Learning where to look for the pancreas. *ArXiv Prepr. ArXiv180403999* (2018).
7. Köster, J. & Rahmann, S. Building and documenting workflows with python-based snakemake. in *German Conference on Bioinformatics 2012* 49–56 (Schloss Dagstuhl–Leibniz-Zentrum für Informatik, 2012).
8. Mölder, F. *et al.* Sustainable data analysis with Snakemake. *F1000Research* **10**, 33 (2025).
9. Eustace, S. & Contributors. Poetry: Python packaging and dependency management made easy. (2018). https://python-poetry.org/
10. Van der Walt, S. *et al.* scikit-image: image processing in Python. *PeerJ* **2**, e453 (2014).
11. R Core Team. *R: A Language and Environment for Statistical Computing*. (R Foundation for Statistical Computing, Vienna, Austria, 2025).
12. Scrucca, L., Fraley, C., Murphy, T. B. & Raftery, A. E. *Model-Based Clustering, Classification, and Density Estimation Using Mclust in R*. (Chapman and Hall/CRC, New York, 2023). doi:10.1201/9781003277965.
13. Wickham, H. ggplot2: Elegant Graphics for Data Analysis. in *ggplot2: Elegant Graphics for Data Analysis* (ed. Wickham, H.) 241–253 (Springer International Publishing, Cham, 2016). doi:10.1007/978-3-319-24277-4_12.

14. Wickham, H., Çetinkaya-Rundel, M. & Grolemund, G. *R for Data Science: Import, Tidy, Transform, Visualize, and Model Data*. (O'Reilly Media, Inc., 2023).

15. Wickham, H., Vaughan, D. & Girlich, M. tidyr: Tidy Messy Data. https://doi.org/10.32614/CRAN.package.tidyr (2024) doi:10.32614/CRAN.package.tidyr.

16. Bates, D., Maechler, M., Bolker, B. & Walker, S. lme4: Linear Mixed-Effects Models using lme4. 1.1-38 https://doi.org/10.32614/CRAN.package.lme4 (2025).

17. Kuznetsova, A., Brockhoff, P. B. & Christensen, R. H. B. lmerTest Package: Tests in Linear Mixed Effects Models. *J. Stat. Softw.* **82**, 1–26 (2017).

18. Lenth, R. V. & Piaskowski, J. emmeans: Estimated Marginal Means, aka Least-Squares Means. https://doi.org/10.32614/CRAN.package.emmeans (2025) doi:10.32614/CRAN.package.emmeans.
