# Supplementary material for: Epithelial cell extrusion underlies starvation-induced cell loss in a sea anemone
Source: Nat Commun. 2026 Jun 17;17:7681. doi: 10.1038/s41467-026-74437-7 (PMC13433931; doi:10.1038/s41467-026-74437-7)
Supplement: Supplementary file 2 [file 41467_2026_74437_MOESM2_ESM.pdf]

nature portfolio

Peer Review File

Epithelial cell extrusion underlies starvation-induced cell loss in a sea anemone

Corresponding Author: Dr Patrick Steinmetz

This file contains all reviewer reports in order by version, followed by all author rebuttals in order by version.

Version 0:

Reviewer comments:

Reviewer #1

(Remarks to the Author)
In this paper, Fournon-Berodia and colleagues describe for the first time cell extrusion in a non-bilaterian animal, the sea anemone Nematostella Vectansis. Using a combination of live imaging and quantitative characterization of rosette/extrusion figures in fixed samples (using a home trained deep learning pipeline) they also characterized very interesting dynamics during the process of starvation allowing to connect clearly modulation of extrusion rate with body size adjustment and nutrient intake. Using cleaved caspase staining and pan caspase inhibitor, they confirm that most of the extrusion are preceded by caspase activation and rely on caspase activity. Surprisingly however, caspase activation does not seem to be sufficient to promote extrusion. The direction of extrusion is mostly apical, with a subset of basal extrusion. The authors also used a combination of drug perturbation and immunostaining to outline a number of pathways modulated in vicinity of dying cells, including a local activation of ERK (as previously found in mammalian cell culture and Drosophila) which seems to dumpen the global rate of death, as well as local mTOR activation.

Overall, the paper provides a number of very novel insights, regarding first the conservation of the process of extrusion (whose phenomenology seems very similar to what was described in other animals) as well as the wide conservation of the local ERK feedback. The paper provides a number of very interesting technological innovation, including live imaging with subcellular resolution of nematostella and a semi automatic pipeline to characterize cell shape and cell extrusion on a large number of fixed samples. Overall, this paper is an important proof of principle that Nematostella could be an excellent model system to study epithelial homeostasis and the role of cell death and cell extrusion in tissue plasticity and adaptation to environmental changes. There are actually very few system to my knowledge allowing to study the link between body size/organ size variation and environment changes which such resolution (apart maybe from the fly gut, where long term imaging is absolutely not trivial). This, combined with the important proof of conservation of extrusion and ERK, justify completely the publication in a journal with a large readership.

The phenomenology is convincing and well performed and the data overall very solid. The part describing the link between starvation and extrusion modulation still have some mechanistic gaps but I don't think this is the main purpose of this article and I don't expect a full understanding of the process at this stage. I still have however some suggestions that could help to clarify some interpretation that would mostly require novel analysis and/or discussion, as well as some details on the methodology.

Main suggestions :

1. At this stage, it remains difficult to interpret the link between food/starvation and modulation of cell extrusion, mostly because of the protocol used by the authors. What was initially the rational to first starve the anemone for 5 days, feed for 1h and starve again ? In this context, it is very hard to disentangle which part of the dynamics is related to starving and which part is related to the transient refeeding. Could the author provide further details on the choice of the protocol ? On a similar line, the authors observe after 10 and 20 days an overall high number of rosettes compared to fully fed condition. Why this high levels is not observed on they first data point after 5 days of starvation ? Is it because 5 days is not sufficient to trigger an increase (and this would have been observed later one even without a 1h feeding), or could it be possible that the 1h feeding has a immediate impact of extrusion (which would seem surprising). I am not calling here for further experiment, but mostly suggesting to add further discussion on these points in the paper.

2. So far, two important parameters are lacking from the starvation analysis to really understand the relevance of cell extrusion for tissue size regulation: namely the evolution of body size and the number of cell division. I will certainly not request the authors to redo all their measurement, but they may have already relevant data in their dataset. Considering that extrusion last 10-20 min, cytokinesis is probably taking place on a similar timescale and should therefore be detectable on fixed sample and movies if happening at a similar rate. Did the author first observed any cell division in the few movies they obtained ? If so, could they give a rough estimate of their probability (relative to extrusion) ? Altenatively, do they find very obvious round cells (most likely cytokinetic) in their fixed sample and could they give a rough estimate of their frequency relative to rosette ? I do understand that the main point of the manuscript is to prove the conservation of extrusion and some local feedback and not necessarily to characterize its role in size plasticity, but having some sense of the role of cell division (which might be completely neglectable relative to extrusion) would help to see whether the overall dynamics is indeed dominated by cell death. It would have been also highly relevant to compare full body size dynamics in context modulating the number of rosette figures (e.g. : ERK inhibition, zvad fmk), but if the data is not there, that can be leaved for futur studies.

3. The rosette detection recall (0.64) suggest that many events are missed by the pipeline (close to 40%). It was not clear to me whether the combined manual curation also included addition of false negative. Could the authors estimate roughly the “F1 score” of their automatic detection combined with manual curation compared with a totally manual detection ? My point here is to get a sense of how many rosette may have been missed, specially if the manual curation only discard false positive and does not deal with false negative.

4. The variation of cell size in the different perturbative experiments is interesting but sometimes hard to interpret. For instance, how to explain the absence of variation of cell size despite the increase of rosette upon inhibition of ERK ? Could it be explained by less division and/or an alteration of whole body size ? It would be interesting to discuss this point. Same for the z-vad-fmk treatment (where there is no change of density despite a reduction of rosette).

5. As rightly acknowledged by the author in the discussion, a variation of rosette figure density in fixed sample may not be related to change of the probability of cell death but to a modulation of the total duration of extrusion. The authors discuss this point for ERK, but it may also be important for the staurosporine treatment. It is surprising in this context that the broad caspase activation does not lead to more rosette detection and one alternative interpretation could be that cell expulsion takes place through completely different remodeling (e.g. : more junction remodeling and T1 transition) hence not leading to clear rosette figure, or alternatively much faster extrusion. In absence of live imaging of this context, I would also suggest to be cautious on the interpretation and clearly indicate these alternative options in the discussion.

6. The observation of caspase in vast majority of rosette early on and the impact of z-vad-fmk on rosette frequency suggest clearly that the vast majority of extrusion are caspase dependant and associated with apoptosis. I believe the authors can conclude more firmly on this in the manuscript.

7. The link between mTOR activation and nutrient recycling through apoptotic body remain a bit weak at this stage. To make such claim, the authors should at least try to correlate the proportion of debris within each neigbouring cells with variation of mTor activity. Also,in this framework, mTor activation should not be observed in early rosette prior to cell body fragmentation. Did the authors observed mTor activation only near late apoptotic cells ? If the authors observed already some mTor activation with early rosette, that would probably argue for an alternative mode of activation. There could be many other reason and ways to activate Tor in those cells (for instance this could be epitatically related to ERK). Overall, the observation of mTor activation is novel and very interesting, but I would probably leave the interpretation for nutrient recycling in the discussion at this stage as it is very interpretative and not clearly demonstrated by the current data.

On a similar topic, in the discussion the authors propose that late constant increase of rosette might be related to nutrient. If so, one may expect that Tor inhibition should lead to an increase of rosette. The basal levels may be too low to see an effect of Tor inhibition in starve context, but there should be a clear impact on rosette in fed animal. Did the author used the Tor inhibitor in a fed context and checked its impact on extrusion/cell death ?

8. The authors propose in the discussion a link between crowding/density and the first wave of extrusion. What are the evidence that the first wave of extrusion is indeed related to cell density changes ? Unless I am mistaken, there is no increase of density between fed animal and during the first days of starvation, which according to me probably argue against a key role of crowding/cell density. Going along the same line, we should then see an increase of cell density upon apoptosis inhibition (caspase inhibition or ERK inhibition) but this is not what the authors see. I would suggest to clarify the arguments supporting the role of crowding or change the statement if this is not fitting with the observable.

Other minor points :

Line 70 : when mentioning “ERK signaling is currently thought to mediate extrusion in a non-cell autonomous fashion” the author should quote Aikin et al elife 2020 (the two reference of this sentence only covered the protective effect of ERK)

Line 124: the duration of extrusion can vary a lot between tissue and the extrusion of enterocyte taking several hours is more of an exception in Drosophila. In the fly embryo, the pupal notum, pupal leg or the abdomen it takes on average 15-30minutes jut like nematostella (check Villars et al Nat Comm 2022 PMC9233712, Toyama et all Science 2008 PMC2757114, Teng et al Development 2017 10.1242/dev.139865, Monier et al Nature 2015, PMID: 25607361). I would suggest to change/nuance this statement.

Line 219, the correct reference for ERK feedback is Valon et al Dev Cell 2021 and not Villars et al 2022

Line 380: the reference provided are not totally correct. First I don't think any of these references have shown that extruding cells produce a stress factor as stated by the author (I would correct this), secondly, only Gagliardi et al and Valon et al 2021 did characterize the prosurvival impact of ERK. Aikin rather showed long range waves promoting cell extrusion. Takeushi characterized Calcium waved generating centripetal movement promoting cell expulsion. Same issue line 387 (none of the paper cited here work on fly).

(Remarks on code availability)

Reviewer #2

(Remarks to the Author)
In the manuscript "Epithelial cell extrusion underlies starvation-induced cell loss in a sea anemone," Fournon-Berodia and colleagues describe the process of epithelial cell extrusion and its relationship to apoptosis and nutrient-dependent tissue dynamics in the sea anemone Nematostella. This process (cell extrusion and rosette formation) has previously been extensively studied in Drosophila and vertebrates, and thus the chief novelty here is the phylogenetic extension to cnidarians and linking the nutrient-dependent growth and shrinkage of Nematostella to cell loss through extrusion. After carefully and quantitatively describing the phenomenon, the authors use a series of pharmaceutical inhibition experiments to probe key nodes in the extrusion pathway- namely apoptosis and ERK activation. Overall, they find that in Nematostella starvation may regulate tissue size through epithelial cell extrusion.

Major comments:
1. Descriptive elements. While important and interesting, I felt the initial narrative of the paper was bogged down in the lengthy descriptive sections and Figures 2 and 3 add little to the key results that could not be summarized in the text but largely moved to supplementary material. Furthermore, the process of extrusion is primarily occurring in the Z-plane, yet XZ scans are not depicted in the figures, making them difficult to interpret. I think the paper could be made clearer and more direct by including XZ data to illustrate extrusion in the main figures.

2. Staurosporine experiments. The authors rely solely on pharmaceutical inhibitor experiments to probe the role of cell death in the epithelial extrusion. Presumably in Nematostella it is challenging or impossible to knock down gene products in juvenile polyps and time consuming to generate relevant mutant alleles. While this is not necessarily a fatal flaw, some effort should be made to control and add depth to the analysis. Of primary concern to this reviewer, the Staurosporine experiments ubiquitously induce stress and in some cases apoptosis, which is an effect on the global field of cells. Extrusion, by definition, involves selection and removal of a single cell and likely requires non-autonomous action in the surrounding wild-type cells. Hence, I am confused how inducing global stress (evidenced by broad activation of ERK1/2) can allow any meaningful interpretation regarding single cells extrusion (as stated in MS line 290). This limitation needs to be directly addressed or the section removed.

3. Extrusion and drug dynamics. In the case of the MEK, Staurosporine, and Q-VD-OPh treatments, animals were exposed to the drug two hours before analysis. How was this single time point determined for all three compounds? To fully evaluate the dynamics of drug induced phenotypes, some rationale should be provided and differential treatment times evaluated for each drug individually. Further, washout experiments would provide important controls for the interpretation of drug effects.

4. Cell death. The authors reported CCS+ central rosette cells throughout all stages, which seems consistent with the interpretation that dying cells are extruded as a direct consequence of apoptosis, as observed in many other organismal systems. Instead, the authors state (line 274) that "extruding cells predominantly undergo apoptosis during the process." I wondered whether the conclusion that dying cells undergo apoptotic extrusion as in other organisms is equally if not more valid, especially in light of the reduction of rosette density following treatment with Q-VD-OPh. Given the concerns about field-wide stress induction in the Staurosporine experiments, how do the authors rule out that extrusion is simply triggered by apoptosis of single cells?

Minor issues.
The authors should revise the entire text for conciseness and clarity, particularly in sections where many contractions are used. These seems to be technically accurate, but I had to repeatedly refer back to the definitions of dpr, T5ds, per mille, "refeeding" etc. Maybe more importantly, mixed verb tenses in the text and legends should be corrected to more uniformly describe published work in present tense and current results in past tense.

(Remarks on code availability)

Version 1:

Reviewer comments:

Reviewer #1

(Remarks to the Author)
The authors have adressed all my concernes mostly by providing further explanations and justifications regarding various experimental choice and adding elements in the discusion. I am now fully supportive for publication.

Just one very minor correction for a reference (similar to one of my previous point) : Line 63, the references should be corrected (Aikin et al only characterised the role of ERK wave in extrusion, if the authors want to mention the prosurvival effect they should quote Gagliardi et al 2021 and Valon et al 2021).

(Remarks on code availability)

Reviewer #2

(Remarks to the Author)
The authors have satisfactorily addressed my concerns, I look forward to seeing this interesting and highly rigorous work published.

(Remarks on code availability)

Open Access This Peer Review File is licensed under a Creative Commons Attribution 4.0 International License, which permits use, sharing, adaptation, distribution and reproduction in any medium or format, as long as you give appropriate credit to the original author(s) and the source, provide a link to the Creative Commons license, and indicate if changes were made.
In cases where reviewers are anonymous, credit should be given to 'Anonymous Referee' and the source.
The images or other third party material in this Peer Review File are included in the article’s Creative Commons license, unless indicated otherwise in a credit line to the material. If material is not included in the article’s Creative Commons license and your intended use is not permitted by statutory regulation or exceeds the permitted use, you will need to obtain permission directly from the copyright holder.
To view a copy of this license, visit https://creativecommons.org/licenses/by/4.0/

Point-by-point response to reviewers' comments

Summary of responses

- We added new quantification data showing that ~71% of pRPS6+ rosettes contain condensed nuclear fragments, supporting an association between TOR activation and apoptotic material.
- We added XZ and YZ views to Figure 3 to show basal translocation of β-catenin-mOrange2 during extrusion.
- We revised our interpretation of extrusion dynamics, replacing our previous cell density-driven model and instead proposing that increased extrusion after refeeding reflects a transient mismatch between proliferation and tissue accommodation.
- We clarified the limitations and interpretation of pharmacological perturbations and justified the choice of short 2-hour incubation windows across drug treatments.
- We clarified the starvation/refeeding protocol and sampling strategy to improve interpretability of extrusion dynamics
- We improved clarity, readibility and consistency throughout the manuscript, including terminology, figure annotations and verb tense usage.

Detailed responses

Reviewer #1 (Remarks to the Author):

In this paper, Fournon-Berodia and colleagues describe for the first time cell extrusion in a non-bilaterian animal, the sea anemone Nematostella Vectansis. Using a combination of live imaging and quantitative characterization of rosette/extrusion figures in fixed samples (using a home trained deep learning pipeline) they also characterized very interesting dynamics during the process of starvation allowing to connect clearly modulation of extrusion rate with body size adjustment and nutrient intake. Using cleaved caspase staining and pan caspase inhibitor, they confirm that most of the extrusion are preceded by caspase activation and rely on caspase activity. Surprisingly however, caspase activation does not seem to be sufficient to promote extrusion. The direction of extrusion is mostly apical, with a subset of basal extrusion. The authors also used a combination of drug perturbation and immunostaining to outline a number of pathways modulated in vicinity of dying cells, including a local activation of ERK (as previously found in mammalian cell culture and Drosophila) which seems to dumpen the global rate of death, as well as local mTOR activation.

Overall, the paper provides a number of very novel insights, regarding first the

conservation of the process of extrusion (whose phenomenology seems very similar to what was described in other animals) as well as the wide conservation of the local ERK feedback. The paper provides a number of very interesting technological innovation, including live imaging with subcellular resolution of nematostella and a semi automatic pipeline to characterize cell shape and cell extrusion on a large number of fixed samples. Overall, this paper is an important proof of principle that Nematostella could be an excellent model system to study epithelial homeostasis and the role of cell death and cell extrusion in tissue plasticity and adaptation to environmental changes. There are actually very few system to my knowledge allowing to study the link between body size/organ size variation and environment changes which such resolution (apart maybe from the fly gut, where long term imaging is absolutely not trivial). This, combined with the important proof of conservation of extrusion and ERK, justify completely the publication in a journal with a large readership.

The phenomenology is convincing and well performed and the data overall very solid. The part describing the link between starvation and extrusion modulation still have some mechanistic gaps but I don't think this is the main purpose of this article and I don't expect a full understanding of the process at this stage. I still have however some suggestions that could help to clarify some interpretation that would mostly require novel analysis and/or discussion, as well as some details on the methodology.

Main suggestions:

1. At this stage, it remains difficult to interpret the link between food/starvation and modulation of cell extrusion, mostly because of the protocol used by the authors. What was initially the rational to first starve the anemone for 5 days, feed for 1h and starve again ? In this context, it is very hard to disentangle which part of the dynamics is related to starving and which part is related to the transient refeeding. Could the author provide further details on the choice of the protocol ?

1a) We thank the reviewer for raising this issue, which allowed us to further clarify and explain the choice of protocol. The starvation/refeeding protocol was chosen to strictly control feeding history across animals and to precisely define the onset of starvation. The brief 1-hour refeeding pulse ensures a reproducible starting point for subsequent starvation while avoiding variability associated with *ad libitum* feeding. At the same time, this pulse is sufficient to trigger a full proliferative response (Pascual-Carreras, 2025; doi:10.1371/journal.pbio.3003525), allowing us to follow its dynamics during subsequent starvation. We have revised the Results section (l. 148–158) to more clearly explain this rationale and the underlying constraints.

On a similar line, the authors observe after 10 and 20 days an overall high number of rosettes compared to fully fed condition. Why this high levels is not observed on they first data point after 5 days of starvation ? Is it because 5 days is not sufficient to trigger an increase (and this would have been observed later one even without a 1h feeding), or could it be possible that the 1h feeding has a immediate impact of extrusion (which would seem surprising). I am not calling here for further experiment, but mostly suggesting to add further discussion on these points in the paper.

1b) We thank the reviewer for raising these questions, which helped identifying an ambiguity in the sampling strategy description in the Results, Methods and the legend of Fig. 3B. Importantly, the 'first data point after 5 days of starvation' (T5ds) was sampled prior to the 1-hour refeeding. We have rephrased the Results text (l. 153, l. 184), Methods (l. 583-4) and Fig. 3B caption (l. 1014-5) to clarify this point and avoid any misunderstanding.

This clarification raises the question of why extrusion rates are higher after refeeding (at 5dpr) compared to T5ds. To address this, we have revised the Results to explicitly report these differences (l. 188-90) and include T5ds in the comparison of cell densities, which are similar between daily fed animals, T5ds, and during the first days after refeeding (l. 176). We have furthermore expanded the Discussion (l. 433-437) to speculate about possible explanations. In particular, we now propose that extrusion is not solely determined by tissue density but may reflect a transient mismatch between cell proliferation and the tissue's capacity to accommodate newly added cells. Low extrusion at T5ds may be explained by low proliferation in a tissue where accommodation is not limiting, given a history of daily feeding. In contrast, the increase in rosette density following refeeding may result from a rapid proliferation burst that is not immediately matched by tissue accommodation, potentially due to limited nutrient resources for extracellular matrix production following the short refeeding pulse.

In addition, we now discuss the possibility that extrusion after daily feeding may transiently increase between 0 and 5 days, as suggested by previous observations of elevated cell loss during this period (Garschall et al. 2024; doi:10.1242/dev.202926), and subsequently decline by T5ds. Together, these additions clarify that extrusion dynamics may depend on both feeding history and nutritional state and cannot be explained by tissue density alone.

2. So far, two important parameters are lacking from the starvation analysis to really understand the relevance of cell extrusion for tissue size regulation: namely the evolution of body size and the number of cell division. I will certainly not request the authors to redo all their measurement, but they may have already relevant data in their dataset. Considering that extrusion last 10-20 min, cytokinesis is probably taking place on a similar timescale and should therefore be detectable on fixed sample and movies if happening at a similar rate. Did the author first observed any

cell division in the few movies they obtained ? If so, could they give a rough estimate of their probability (relative to extrusion) ? Altenatively, do they find very obvious round cells (most likely cytokinetic) in their fixed sample and could they give a rough estimate of their frequency relative to rosette ?

I do understand that the main point of the manuscript is to prove the conservation of extrusion and some local feedback and not necessarily to characterize its role in size plasticity, but having some sense of the role of cell division (which might be completely neglectable relative to extrusion) would help to see whether the overall dynamics is indeed dominated by cell death. It would have been also highly relevant to compare full body size dynamics in context modulating the number of rosette figures (e.g. : ERK inhibition, zvad fmk), but if the data is not there, that can be leaved for futur studies.

2) We thank the reviewer for raising this point. We agree that quantifying changes in cell proliferation, total cell numbers and body size would be highly informative to assess the relative contributions of cell extrusion and proliferation to tissue and body size regulation, both under physiological conditions and following inhibitor treatments. Addressing this comprehensively would, however, require integrating whole-body imaging with flow cytometry approaches, which is beyond the scope of the present study. In addition, the short incubation times used for pharmacological perturbations were specifically chosen to capture acute effects on rosette formation while minimizing secondary effects. This likely also explains why we do not detect significant changes in epidermal cell density after most drug treatments (please see also 4) and response 3a) to Reviewer #2).

Nevertheless, we re-examined our live and fixed imaging datasets as suggested by the reviewer but did not detect any clear mitotic events. This likely reflects the very short duration of M-phase in *Nematostella*, as supported by our previous work, where phospho-Histone H3-positive cells were rarely detected by flow cytometry or imaging despite robust EdU incorporation following 30-minute pulses (Pascual-Carreras et al., 2025; doi:10.1371/journal.pbio.3003525). Together, these observations indicate that mitoses are difficult to capture in snapshots or short recordings in the *Nematostella* epidermis.

While we did not directly quantify proliferation in the epidermis in this study, we previously found that whole-polyp proliferation rates are low during starvation and transiently increase after feeding before declining again (Garschall et al., 2024; doi:10.1242/dev.202926). We have incorporated this context in the revised Results (l. 148–158; and Discussion (l. 424ff; Fig. 7G–I).

3. The rosette detection recall (0.64) suggest that many events are missed by the pipeline (close to 40%). It was not clear to me whether the combined manual curation also included addition of false negative. Could the authors estimate roughly

the “F1 score” of their automatic detection combined with manual curation compared with a totally manual detection ? My point here is to get a sense of how many rosette may have been missed, specially if the manual curation only discard false positive and does not deal with false negative.

3) We thank the reviewer for highlighting the need for clarification regarding model performance and the role of manual curation. We have revised the manuscript accordingly by editing the Supplementary Methods section 'Deep Learning Model Performance' and by adding additional results illustrating the event detection rate in section '4.5. Rosette Detection Model' of the Supplementary Methods. These provide the following clarifications:

The reported recall reflects pixel-level accuracy, which strongly penalizes boundary imprecision and therefore underestimates event-level performance.

- **Does a recall of 0.64 imply that ~40% of events are missed**? No. The reported recall reflects pixel-level accuracy, which strongly penalizes boundary imprecision and therefore underestimates event-level performance. When evaluated at the level of individual rosettes, 82.9% of events (223/269) contained ≥10% correctly predicted pixels and were thus detected by the model. Even in cases with partial segmentation, these predictions generated clear visual signals that were readily identifiable during manual review.
- **Does manual curation include recovery of false negatives**? Yes. Manual curation was not limited to model-flagged regions. Instead, the human-in-the-loop reviewed the full prediction heatmap overlaid on the entire image, allowing identification and addition of missed events (false negatives) as well as removal of false positives. This approach ensures that event detection is not constrained by the initial model output
- **Can a combined F1 Score be estimated?** A formal F1 score for the combined automatic and manual workflow would require an independent, fully manual ground truth annotation, which is beyond the scope of this study. However, because the full image is systematically reviewed and missed events are actively recovered, we expect the effective false-negative rate of the curated dataset to approach that of human annotation. While we have not explicitly quantified human annotator error (e.g., via multiple independent annotations), we estimate it to be in the low single-digit percentage range.

4. The variation of cell size in the different perturbative experiments is interesting but sometimes hard to interpret. For instance, how to explain the absence of variation of cell size despite the increase of rosette upon inhibition of ERK ? Could it be explained by less division and/or an alteration of whole body size ? It would be interesting to

discuss this point. Same for the z-vad-fmk treatment (where there is no change of density despite a reduction of rosette).

4) We agree with the reviewer that these are all interesting and relevant questions. Shortly, we believe that our 2-hour incubation periods have been too short to result in significant, global changes in cell density. These short incubation times were chosen to reduce any secondary effects as much as possible, but we are aware that this choice may have limited the interpretation of effect on a tissue- or even whole-body level. We have added this possibility to the discussion (l. 366-72 and 397-99). We kindly refer the reviewer to our response 3a) to Reviewer #2, for a more detailed explanation of our rationale.

5. As rightly acknowledged by the author in the discussion, a variation of rosette figure density in fixed sample may not be related to change of the probability of cell death but to a modulation of the total duration of extrusion. The authors discuss this point for ERK, but it may also be important for the staurosporine treatment. It is surprising in this context that the broad caspase activation does not lead to more rosette detection and one alternative interpretation could be that cell expulsion takes place through completely different remodeling (e.g. : more junction remodeling and T1 transition) hence not leading to clear rosette figure, or alternatively much faster extrusion. In absence of live imaging of this context, I would also suggest to be cautious on the interpretation and clearly indicate these alternative options in the discussion.

5) We fully agree with the reviewer's concerns and have added speed and rosette-independent expulsion mechanisms as alternative explanations to the discussion of the Staurosporine experiments (l. 391-95).

6. The observation of caspase in vast majority of rosette early on and the impact of z-vad-fmk on rosette frequency suggest clearly that the vast majority of extrusion are caspase dependant and associated with apoptosis. I believe the authors can conclude more firmly on this in the manuscript.

6) We thank the reviewer for this suggestion. We have revised the Results (l.281-3) and Discussion (l. 399–402) to more clearly and firmly state that Caspase-mediated apoptosis appears necessary, but not sufficient, for extrusion initiation.

7. The link between mTOR activation and nutrient recycling through apoptotic body remain a bit weak at this stage. To make such claim, the authors should at least try to correlate the proportion of debris within each neigbouring cells with variation of mTor activity.

7a) We thank the reviewer for this suggestion. Direct co-labelling of pRPS6 (TOR activity) and CCS would be required to quantitatively correlate apoptotic debris with TOR signalling in rosette cells. However, this is currently not technically feasible, as

both antibodies are raised in rabbit. To address this point, we instead quantified the presence of condensed nuclear debris within pRPS6+ rosettes. We found that ~71% of pRPS6+ rosettes contain nuclear fragments, consistent with the presence of apoptotic material in rosette cells with active TOR signalling. We have included these new quantifications in the Results (l. 299-301) and the Discussion (l. 407-8).

Also,in this framework, mTor activation should not be observed in early rosette prior to cell body fragmentation. Did the authors observed mTor activation only near late apoptotic cells ? If the authors observed already some mTor activation with early rosette, that would probably argue for an alternative mode of activation.

7b) We thank the reviewer for the suggestion. This point was already addressed in our initial manuscript, and we have further extended the analysis in the revised version by increasing the sample size to 24 rosettes. We confirm our previous observation that TOR activation is largely restricted to late-stage rosettes (22/24; ~92%), with only two instances detected at mid-stage. We updated the Results (l. 297-99) accordingly.

There could be many other reason and ways to activate Tor in those cells (for instance this could be epitatically related to ERK). Overall, the observation of mTor activation is novel and very interesting, but I would probably leave the interpretation for nutrient recycling in the discussion at this stage as it is very interpretative and not clearly demonstrated by the current data.

7c) We thank the reviewer for this comment. As suggested, we have reduced the emphasis on nutrient recycling in the Results (l. 284-309) and toned down our interpretation of TOR activation in the Discussion (l. 403-16). We now present the efferocytosis hypothesis only briefly as a motivation for investigating TOR signalling and adopt a more careful interpretation in the Results (l. 286-88). In the Discussion, we have also incorporated the previously missing point that TOR activity is not required for rosette formation into our argumentation (l. 409-10).

On a similar topic, in the discussion the authors propose that late constant increase of rosette might be related to nutrient. If so, one may expect that Tor inhibition should lead to an increase of rosette. The basal levels may be too low to see an effect of Tor inhibition in starve context, but there should be a clear impact on rosette in fed animal. Did the author used the Tor inhibitor in a fed context and checked its impact on extrusion/cell death ?

7d) We thank the reviewer for this interesting suggestion. We agree that testing whether reduced nutrient availability in fed animals affects extrusion would be highly informative. However, pharmacological inhibition of TOR does not directly mimic a simple reduction in nutrient or energy levels, as the cellular energy or metabolic status (e.g. ATP levels) is not solely determined by TOR signalling and can vary independently. Consistent with this, our data show that TOR inhibition does not

significantly affect rosette density, indicating that extrusion is not directly controlled by TOR activity in this system. Moreover, because TOR signaling integrates multiple inputs and directly regulates processes such as cell growth and proliferation, its inhibition in fed animals would introduce pleiotropic effects, making it difficult to disentangle direct from indirect consequences on extrusion.

We therefore interpret our results as suggesting that the influence of prolonged starvation on extrusion may be mediated through TOR-independent mechanisms. Exploring these links, for example via directly manipulating cellular ATP levels or signaling pathways (e.g. AMPK), would be an important direction for future work, but is beyond the scope of the present study.

8. The authors propose in the discussion a link between crowding/density and the first wave of extrusion. What are the evidence that the first wave of extrusion is indeed related to cell density changes ? Unless I am mistaken, there is no increase of density between fed animal and during the first days of starvation, which according to me probably argue against a key role of crowding/cell density. Going along the same line, we should then see an increase of cell density upon apoptosis inhibition (caspase inhibition or ERK inhibition) but this is not what the authors see. I would suggest to clarify the arguments supporting the role of crowding or change the statement if this is not fitting with the observable.

8) We thank the reviewer for this important point regarding the Discussion. We agree that our initial interpretation was too simplistic and that our data do not support a direct link between cell density and extrusion. In particular, as the reviewer correctly notes, we do not observe an increase in tissue density between continuously fed animals and polyps at T_{5ds}, prior to the rise in extrusion. We have therefore revised both the Results (l. 194-96) and Discussion (l. 424-443) to clarify that extrusion is unlikely to be driven by cell density alone. Instead, we propose that the increase in extrusion following refeeding reflects a transient imbalance between cell proliferation and tissue's capacity to accommodate new cells (e.g., due to limited ECM production relative to rapid cell addition).

Regarding the reviewer’s suggestion that inhibition of apoptosis or ERK signalling should increase cell density, we note that the 2-hour treatments used in our experiments were designed to capture acute effects and are likely too short to result in measurable changes in cell density. We have clarified this point in the revised Results (l. 219, 261-3) and Discussion (l. 369-72; 397-99; see also response 3a) to reviewer #2).

Other minor points:

Line 70: when mentioning “ERK signaling is currently thought to mediate extrusion in a non-cell autonomous fashion” the author should quote Aikin et al elife 2020 (the two reference of this sentence only covered the protective effect of ERK)

We apologize for this omission and have corrected this issue (Ref. 17; l. 63).

Line 124: the duration of extrusion can vary a lot between tissue and the extrusion of enterocyte taking several hours is more of an exception in Drosophila. In the fly embryo, the pupal notum, pupal leg or the abdomen it takes on average 15-30minutes jut like nematostella (check Villars et al Nat Comm 2022 PMC9233712, Toyama et all Science 2008 PMC2757114, Teng et al Development 2017 10.1242/dev.139865, Monier et al Nature 2015, PMID: 25607361). I would suggest to change/nuance this statement.

We thank the reviewer for pointing this out. We have changed the statement accordingly (l. 117-20).

Line 219, the correct reference for ERK feedback is Valon et al Dev Cell 2021 and not Villars et al 2022

We apologize for the mistake, which we have corrected (Ref. 16, l. 202).

Line 380: the reference provided are not totally correct. First I don’t think any of these references have shown that extruding cells produce a stress factor as stated by the author (I would correct this), secondly, only Gagliardi et al and Valon et al 2021 did characterize the prosurvival impact of ERK. Aikin rather showed long range waves promoting cell extrusion. Takeushi characterized Calcium waved generating centripetal movement promoting cell expulsion. Same issue line 387 (none of the paper cited here work on fly).

We thank the reviewer for suggesting these corrections, which we have implemented (l. 351-52, 356-57).

Reviewer #2 (Remarks to the Author):

In the manuscript "Epithelial cell extrusion underlies starvation-induced cell loss in a sea anemone," Fournon-Berodia and colleagues describe the process of epithelial cell extrusion and its relationship to apoptosis and nutrient-dependent tissue dynamics in the sea anemone Nematostella. This process (cell extrusion and rosette formation) has previously been extensively studied in Drosophila and vertebrates, and thus the chief novelty here is the phylogenetic extension to cnidarians and linking the nutrient-dependent growth and shrinkage of Nematostella to cell loss through extrusion. After carefully and quantitatively describing the phenomenon, the authors use a series of pharmaceutical inhibition experiments to probe key nodes in the extrusion pathway- namely apoptosis and ERK activation. Overall, they find that in Nematostella starvation may regulate tissue size through epithelial cell extrusion.

Major comments:
1. Descriptive elements. While important and interesting, I felt the initial narrative of the paper was bogged down in the lengthy descriptive sections and Figures 2 and 3 add little to the key results that could not be summarized in the text but largely moved to supplementary material.

1a) We thank the reviewer for this suggestion. While we appreciate the concern regarding the length of the descriptive sections, we have chosen to retain these parts and Figures 2 and 3 in the main manuscript. We consider the phenomenological description of cell extrusion in a non-bilaterian animal to be a central contribution and key novelty of this study. These establish an essential reference framework for future quantitative and mechanistic analyses of cell extrusion in *Nematostella*. We therefore believe that presenting these observations and quantifications in the main figures, rather than in the Supplementary Material, will make them more accessible and useful for the field.

Furthermore, the process of extrusion is primarily occurring in the Z-plane, yet XZ scans are not depicted in the figures, making them difficult to interpret. I think the paper could be made clearer and more direct by including XZ data to illustrate extrusion in the main figures.

1b) We thank the reviewer for this helpful suggestion. During live imaging, we faced a trade-off between increasing z-resolution or temporal resolution. Given the short duration of rosette formation (~12 mins), we prioritised temporal resolution (2-3 stacks/min) over high z-resolution, which would have required several minutes per stack and risked under-sampling the dynamics of the process. Despite the relatively low z-resolution, we believe that the added XZ reconstructions substantially improves the visualisation of basal translocation of the β-catenin-mOrange2 protein. We have therefore implemented the reviewer's suggestion and added xz and xy views to Figure 2H1-I6.

2. Staurosporine experiments. The authors rely solely on pharmaceutical inhibitor experiments to probe the role of cell death in the epithelial extrusion. Presumably in Nematostella it is challenging or impossible to knock down gene products in juvenile polyps and time consuming to generate relevant mutant alleles. While this is not necessarily a fatal flaw, some effort should be made to control and add depth to the analysis. Of primary concern to this reviewer, the Staurosporine experiments ubiquitously induce stress and in some cases apoptosis, which is an effect on the global field of cells. Extrusion, by definition, involves selection and removal of a single cell and likely requires non-autonomous action in the surrounding wild-type cells. Hence, I am confused how inducing global stress (evidenced by broad activation of ERK1/2) can allow any meaningful interpretation regarding single cells

extrusion (as stated in MS line 290). This limitation needs to be directly addressed or the section removed.

2) We thank the reviewer for raising this important point. We agree that Staurosporine induces a global stress response and therefore does not fully recapitulate the physiological context of epithelial cell extrusion. However, at the concentration and duration used throughout the manuscript (0.5μM, 2-hour incubation), apoptosis occurs predominantly in single, spatially dispersed cells rather than in large contiguous regions, as shown in Supplementary Fig. 8C,C'. We therefore consider this spatial pattern of apoptosis induction suitable to assess whether isolated apoptotic cells are able to induce rosette formation. Under these conditions, we did not observe an increase in rosette density, indicating that apoptosis in individual cells is not sufficient to trigger rosette formation in *Nematostella*.

As already noted in the original manuscript, Staurosporine also induces a broad increase in ERK activity across the epidermis, consistent with a global stress response. We therefore interpret these experiments cautiously and use them primarily to test the sufficiency of apoptosis for extrusion initiation, rather than as a model for physiological extrusion. We have clarified these points and toned down our conclusion in the Results (l. 261-3; 269-71) and Discussion (l. 387-88).

3. Extrusion and drug dynamics. In the case of the MEK, Staurosporine, and Q-VD-OPh treatments, animals were exposed to the drug two hours before analysis. How was this single time point determined for all three compounds? To fully evaluate the dynamics of drug induced phenotypes, some rationale should be provided and differential treatment times evaluated for each drug individually.

3a) We thank the reviewer for raising this point, which allows us to clarify our choice of treatment duration. To determine an appropriate incubation time, we surveyed the literature (particularly in *Nematostella* and *Hydra*), where treatments are often ≥24 h. Such long exposures are not compatible with our aim to study a rapid process such as cell extrusion (~12 minutes). We therefore performed pilot experiments using a range of concentrations and incubation times between 2 and 24 h, and selected 2 hours as the shortest duration expected to allow sufficient drug penetration and achieve effective perturbation while minimizing secondary effects. This was confirmed, for example, by loss of pERK1/2 upon U0126 treatment and reduction of CCS signal upon Q-VD-OPh treatment (Fig. S7-8). Using a consistent incubation time across all treatments also enables comparability between conditions. This rationale has now been clarified in the revised Methods (l. 519-24).
We agree that a full time-course analysis would provide additional insight into the dynamics of drug-induced phenotypes. However, such analyses are beyond the scope of the present study, which focuses on studying extrusion dynamics under defined starvation and perturbation conditions.

Further, washout experiments would provide important controls for the interpretation of drug effects.

3b) We thank the reviewer for this suggestion. We agree that washout experiments can be informative to assess reversibility and to distinguish primary from secondary effects, particularly in the context of long-term drug treatments. In our study, however, we deliberately used short 2-hours incubation times to minimize secondary effects while capturing acute changes in rosette formation and dynamics (see Response 3a) above).

Under these conditions, we observed either modest or no significant changes in rosette and tissue density, suggesting that the measured effects reflect acute responses rather than cumulative or irreversible alterations. We therefore believe that washout experiments would be unlikely to add additional insight or substantially change the interpretation of our results. We have clarified that our treatments are primarily intended to capture acute responses in the revised Methods (l. 519-24) and Discussion (l. 369-72).

4. Cell death. The authors reported CCS+ central rosette cells throughout all stages, which seems consistent with the interpretation that dying cells are extruded as a direct consequence of apoptosis, as observed in many other organismal systems. Instead, the authors state (line 274) that “extruding cells predominantly undergo apoptosis during the process.” I wondered whether the conclusion that dying cells undergo apoptotic extrusion as in other organisms is equally if not more valid, especially in light of the reduction of rosette density following treatment with Q-VD-OPh.

4a) We thank the reviewer for this comment, and as also pointed out by reviewer #1 (comment 6), we have adjusted the results section as suggested (l. 253–255) and in addition also revised the discussion (l. 400–401) to more clearly and firmly state the consistent association of Caspase activity in extruding cells in the *Nematostella* epidermis.

Given the concerns about field-wide stress induction in the Staurosporine experiments, how do the authors rule out that extrusion is simply triggered by apoptosis of single cells?

4b) With regard to the tissue-wide stress induced by Staurosporine treatment, as also raised in comment 2, we acknowledge the methodological limitations of this approach and have revised the Results (l. 260-63, 269-271) and Discussion (l. 387-90) to clarify our interpretation. Importantly, although Staurosporine induces a global stress response, we predominantly detect CCS+ cells as isolated, spatially dispersed events rather than widespread, simultaneous apoptosis across the epidermis. This suggests that, under our experimental conditions, apoptosis is induced in individual

cells, allowing us to assess whether single apoptotic events are sufficient to trigger extrusion.

Minor issues.
The authors should revise the entire text for conciseness and clarity, particularly in sections where many contractions are used. These seems to be technically accurate, but I had to repeatedly refer back to the definitions of dpr, T5ds, per mille, “refeeding” etc. Maybe more importantly, mixed verb tenses in the text and legends should be corrected to more uniformly describe published work in present tense and current results in past tense.

We thank the reviewer for this helpful feedback. We have revised the manuscript to improve clarity, consistency and readability, as well as to harmonize verb tenses throughout the text and figure legends. Specifically:

- We further clarified the abbreviation 'dpr' by explicitly adding it to define 'days post-refeeding' also into Fig. 3.

- To improve clarity to our sampling strategy in the experiments described in Fig. 4, 5, S8, S9, we added asterisks to the sampling scheme in Fig. 3B and revised the legend accordingly (l. 1018).

- We rephrased and expanded the description of the sampling strategy in the Results (l. 151-53) to clarify the meaning of T5ds, refeeding, and related terms. We also clarified later in the text that T5ds corresponds to the time point before feeding (l.184).

- We have carefully revised and standardize verb tenses throughout the manuscript, using present tense for published work and past tense for our results, as suggested.
