# Supplementary material for: Epithelial cell extrusion underlies starvation-induced cell loss in a sea anemone
Source: Nat Commun. 2026 Jun 17;17:7681. doi: 10.1038/s41467-026-74437-7 (PMC13433931; doi:10.1038/s41467-026-74437-7)
Supplement: Supplementary file 3 [file 41467_2026_74437_MOESM3_ESM.pdf]

Description of Additional Supplementary Files

Supplementary Data 1 – Manual quantifications of cell extrusion time–lapse data, pERK1/2+ rosettes, CCS+ rosettes and pRSP6+ rosettes (Fig. 1, Fig. 4 B', C', D', Fig. 5 A', B', C')

Supplementary Data 2 – Rosette density analysis of daily fed dataset (Fig. 3E)

Supplementary Data 3 – Cell area analysis of daily fed dataset (Fig. 3C, Supplementary Fig. 5 D)

Supplementary Data 4 – Statistical analysis between daily fed vs long starved (T10–T20) datasets (Fig. 3C–F, Supplementary Fig. 5 D, E)

Supplementary Data 5 – Rosette density analysis of starvation dataset (Fig. 3F)

Supplementary Data 6 – Cell area analysis of starvation dataset (Fig. 3D, Supplementary Fig. 5E)

Supplementary Data 7 – Rosette density analysis after AZD8055 treatment (Supplementary Fig. 9C)

Supplementary Data 8 – Cell area analysis after AZD8055 treatment (Supplementary Fig. 9D)

Supplementary Data 9 – Rosette density analysis after U0126 treatment (Fig. 4E)

Supplementary Data 10 – Cell area analysis after U0126 treatment (Supplementary Fig. 7C)

Supplementary Data 11 – Rosette density analysis after Q–VD–OPh treatment (Fig. 5E)

Supplementary Data 12 – Cell area analysis after Q–VD–OPh treatment (Supplementary Fig. 8J)

Supplementary Data 13 – Rosette density analysis after Staurosporine treatment (Fig. 5E)

Supplementary Data 14 – Cell area analysis after Staurosporine treatment (Supplementary Fig. 8G)

Supplementary Movie 1 – *In vivo* timelapse recording using confocal microscopy showing epidermal cell extrusion in a β–Catenin–mOrange2 CRISPR/Cas9 transgenic *Nematostella vectensis* juvenile polyp

Supplementary Movie 2 – *In vivo* timelapse recording using confocal microscopy showing putative apical cell extrusion of a condensed, Hoechst–labelled nucleus in the epidermis of a β–Catenin–mOrange2 transgenic *Nematostella* juvenile polyp.
