# Supplementary material for: Epithelial cell extrusion underlies starvation-induced cell loss in a sea anemone
Source: Nat Commun. 2026 Jun 17;17:7681. doi: 10.1038/s41467-026-74437-7 (PMC13433931; doi:10.1038/s41467-026-74437-7)
Supplement: Supplementary file 4 [file 41467_2026_74437_MOESM7_ESM.pdf]

nature portfolio

Corresponding author(s): Patrick R. H. Steinmetz

Last updated by author(s): Jun 1, 2026

Reporting Summary

Nature Portfolio wishes to improve the reproducibility of the work that we publish. This form provides structure for consistency and transparency in reporting. For further information on Nature Portfolio policies, see our Editorial Policies and the Editorial Policy Checklist.

Statistics

For all statistical analyses, confirm that the following items are present in the figure legend, table legend, main text, or Methods section.

n/a	Confirmed	
☐	☒	The exact sample size (*n*) for each experimental group/condition, given as a discrete number and unit of measurement
☐	☒	A statement on whether measurements were taken from distinct samples or whether the same sample was measured repeatedly
☐	☒	The statistical test(s) used AND whether they are one- or two-sided<br>*Only common tests should be described solely by name; describe more complex techniques in the Methods section.*
☐	☒	A description of all covariates tested
☐	☒	A description of any assumptions or corrections, such as tests of normality and adjustment for multiple comparisons
☐	☒	A full description of the statistical parameters including central tendency (e.g. means) or other basic estimates (e.g. regression coefficient) AND variation (e.g. standard deviation) or associated estimates of uncertainty (e.g. confidence intervals)
☐	☒	For null hypothesis testing, the test statistic (e.g. *F*, *t*, *r*) with confidence intervals, effect sizes, degrees of freedom and *P* value noted<br>*Give P values as exact values whenever suitable.*
☒	☐	For Bayesian analysis, information on the choice of priors and Markov chain Monte Carlo settings
☐	☒	For hierarchical and complex designs, identification of the appropriate level for tests and full reporting of outcomes
☒	☐	Estimates of effect sizes (e.g. Cohen's *d*, Pearson's *r*), indicating how they were calculated

Our web collection on statistics for biologists contains articles on many of the points above.

Software and code

Policy information about availability of computer code

Data collection	Data acquisition was performed using proprietary microscope control software associated with the Olympus FV3000 confocal microscope (Olympus v. 2.4.1.198). No additional custom or third-party software was used for data collection beyond microscope control and image acquisition in this study.
Data analysis	Image data processing and analysis were performed using a combination of open-source computational tools and custom code, as described in the Methods and Supplementary Notes. Image preprocessing performed using Fiji (based on ImageJ). Initial cell segmentation was carried out using Cellpose (Cyto3). Automatic rosette detection was implemented using a custom deep learning pipeline based on an Attention U-Net model. The model (huggingface.co/NoahBruderer/nematostella_rosette_detection) was trained on manually annotated datasets and applied across all imaging datasets. The full analysis workflow, including segmentation, classification, and quantification, was executed using custom scripts (https://doi.org/10.5281/zenodo.20479352) within a managed computational environment (see Supplementary Notes for details). Statistical analysis and data visualization were performed in R (v. 4.5.2). Cell classification via Gaussian Mixture Models, GMM fitting and downstream analysis: All statistical analyses and graphical representations of data were performed using R (v. 4.5.2 (2025-10-31), https://www.r-project.org/) and the following packages: mclust (v. 6.1.2), ggplot2 (v. 4.0.0), dplyr (v. 1.1.4), tidyr (v. 1.3.1), lme4 (v. 1.1.37), lmerTest (v. 3.1.3, using Type III ANOVA, Satterthwaite's approximation), emmeans (v. 2.0.0), mclust (v. 6.1.2),

For manuscripts utilizing custom algorithms or software that are central to the research but not yet described in published literature, software must be made available to editors and reviewers. We strongly encourage code deposition in a community repository (e.g. GitHub). See the Nature Portfolio guidelines for submitting code & software for further information.

Data

Policy information about availability of data

All manuscripts must include a data availability statement. This statement should provide the following information, where applicable:

- Accession codes, unique identifiers, or web links for publicly available datasets
- A description of any restrictions on data availability
- For clinical datasets or third party data, please ensure that the statement adheres to our policy

The manual training dataset generated for the machine-learning pipeline to identify rosettes, pre-segmented image files, and the raw image segmentation and cell-level data generated in this study have been deposited in the Zenodo public data repository available under: 10.5281/zenodo.19726756. Transgenic lines are available upon request.

Research involving human participants, their data, or biological material

Policy information about studies with human participants or human data. See also policy information about sex, gender (identity/presentation), and sexual orientation and race, ethnicity and racism.

Reporting on sex and gender	n/a
Reporting on race, ethnicity, or other socially relevant groupings	n/a
Population characteristics	n/a
Recruitment	n/a
Ethics oversight	n/a

Note that full information on the approval of the study protocol must also be provided in the manuscript.

Field-specific reporting

Please select the one below that is the best fit for your research. If you are not sure, read the appropriate sections before making your selection.

☒ Life sciences ☐ Behavioural & social sciences ☐ Ecological, evolutionary & environmental sciences

For a reference copy of the document with all sections, see nature.com/documents/nr-reporting-summary-flat.pdf

Life sciences study design

All studies must disclose on these points even when the disclosure is negative.

Sample size

Sample size for the machine-learning image segmentation pipeline model training dataset consisted of: 268 confocal images; 214 training / 54 validation and 269 annotated rosette instances in validation set. Sample sizes were not determined using a formal a priori statistical power calculation. Instead, sample sizes were based on established practice in quantitative imaging studies in Nematostella vectensis and similar developmental and cell biology systems, as well as on pilot experiments assessing variability in cell segmentation and rosette frequency.

For image segmentation pipeline datasets, approximately 10–15 animals per condition or timepoint were analysed, with 2–3 image stacks acquired per animal, resulting in multiple independent observations per biological replicate. For the starvation and daily feeding experiments, 2 and 3 independent biological replicates, respectively, were included per timepoint.

In addition, the drug dataset comprised images across four different drug treatments, two timepoints, and corresponding DMSO controls, with three biological replicates per condition. This sampling depth was chosen to ensure robust estimation of replicate-to-replicate variability in mixed-effects models and to allow reliable estimation of condition and timepoint effects despite inherent biological and imaging heterogeneity.

For the immunohistochemistry image data no a priori sample size was established. Instead, experiments were repeated at least 3 times to ensure consitency of the stainings, and therefore a representative image was then selected to exemplify results in the manuscript.

Data exclusions

Data exclusion was limited to automated, predefined quality-control steps applied uniformly across all quantitative datasets prior to statistical analysis.

In the image-based segmentation and rosette ID data analyses, cells were excluded during preprocessing if they failed segmentation quality criteria, including extreme outliers in cell area (top and bottom 0.01% of values within each image) or exceeded a biologically defined maximum size threshold (100 µm²) as detailed in the Supplementary Notes. Entire images were excluded from GMM-based classification only if fewer than 10 segmented cells remained after quality control or if Gaussian mixture model fitting failed to converge, as detailed in the Supplementary Notes.

All exclusion steps were applied uniformly across conditions and were not based on experimental outcome. No manual exclusion of data points was performed after inspection of results.

No animals, images, or experimental conditions were excluded based on statistical significance or biological interpretation.

Replication	All major experimental findings were reproduced across independent biological replicates.<br>Starvation experiments were conducted in two independent biological replicates, and drug perturbation experiments were performed across three independent biological replicates per condition. Daily feeding experiments were performed using three independent biological batches.<br>For imaging-based quantifications (cell area, rosette density, and class proportions), consistent trends were observed across replicates and were further confirmed using mixed-effects models accounting for replicate structure. In all cases where multiple statistical approaches were used (parametric mixed models and non-parametric tests), results were consistent in direction and interpretation.<br>No major findings were observed to be non-reproducible between independent replicates; variability was captured quantitatively through the inclusion of replicate as a random effect in statistical models.<br>Similarly for immunofluorescence image data, video data and manual quantifications, all observations were reproducible. Specifically for video data and manual quantifications the number of observations is indicated in the Supplementary Data 1 .
Randomization	For the image segmentation pipeline, images were randomly assigned to train/validation sets (80/20 split) (see Supplementary Notes). Animals were allocated to experimental conditions based on experimental design rather than random assignment.<br>For starvation experiments, animals were sampled based on feeding regime. For drug treatments, animals were distributed across treatment and control conditions in parallel, with each condition including independent biological replicates. Controls were always processed in parallel with corresponding treated samples (DMSO-matched controls).<br>No formal randomization procedure was applied, as experimental grouping was determined by developmental stage (juveniles of similar size), feeding regime, or pharmacological exposure, which are inherently time-structured interventions.
Blinding	Blinding was not implemented during image acquisition or analysis.<br>This is because image segmentation, rosette detection, and cell classification were performed using automated or semi-automated computational pipelines (Cellpose-based segmentation, Gaussian mixture models, and deep-learning-based rosette prediction), followed by standardized quantitative analysis in R.<br>Where manual correction was required (e.g., segmentation refinement or rosette annotation training sets), it was performed prior to statistical comparisons and without knowledge of downstream group-level outcomes in the final analysis pipeline.<br>Given the computational nature of the quantification and the objective, algorithm-based definition of outcomes, blinding was not considered necessary.

Reporting for specific materials, systems and methods

We require information from authors about some types of materials, experimental systems and methods used in many studies. Here, indicate whether each material, system or method listed is relevant to your study. If you are not sure if a list item applies to your research, read the appropriate section before selecting a response.

Materials & experimental systems

n/a	Involved in the study
☐	☒ Antibodies
☒	☐ Eukaryotic cell lines
☒	☐ Palaeontology and archaeology
☐	☒ Animals and other organisms
☒	☐ Clinical data
☒	☐ Dual use research of concern
☒	☐ Plants

Methods

n/a	Involved in the study
☒	☐ ChIP-seq
☒	☐ Flow cytometry
☒	☐ MRI-based neuroimaging

Antibodies

Antibodies used	Cadherin1 antibody (rat polyclonal, Pukhlyakova et al. 2019, 1:500) was custom-made and gifted by the Technau lab, as described in the Methods section. In addition, the following commercial antibodies were used: Cleaved Caspase Substrate Motif [DE(T/S/A)D] MultiMab® (Rabbit monoclonal, Cell Signaling Technology, Cat# 8698, 1:200), Phospho-p38 MAPK (Thr180/Tyr182) (D3F9) XP® Alexa Fluor® 594 conjugate (Rabbit monoclonal, Cell Signaling Technology, Cat# 8632S, 1:50), Anti-Phospho-S6 Ribosomal Protein (Ser235/236) (Rabbit monoclonal, Cell Signaling Technology, Cat# 4858, 1:200), Phospho-p44/42 MAPK (Erk1/2) (Thr202/Tyr204) (D13.14.4E) XP® (Rabbit monoclonal, Cell Signaling Technology, Cat# 4370T, 1:100–1:200), and Phospho-p38 MAPK (Thr180/Tyr182) (D3F9) XP® (Rabbit monoclonal, Cell Signaling Technology, Cat# 4511T, 1:100). Secondary antibodies included Goat anti-Rabbit IgG (H+L) DyLight 488 conjugate (Life Technologies, Cat# 35552, 1:1000) and Goat anti-Rat IgG (H+L) Alexa Fluor® 633 conjugate (Life Technologies, Cat# A21094, 1:1000).
Validation	The custom antibody detecting Cadherin-1 used in our experiments was kindly provided by the Technau lab. It is a rat polyclonal antibody corresponding to Nematostella Cadherin1 and was validated by Western blot and immunofluorescence (doi: https://doi.org/10.1242/dev.174623). All commercial antibodies used in this study had been previously successfully implemented in Hydra and Nematostella (doi:https://doi.org/10.1016/j.cub.2025.10.061, https://doi.org/10.1073/pnas.2204122119, https://doi.org/10.1371/journal.pbio.3003525). In addition, for the commercial antibodies multiple sequence alignments with the published epitope against the Nematostella conserved region were performed to assess compatibility and we validated all primary antibodies using immunofluorescence.

Animals and other research organisms

Policy information about studies involving animals; ARRIVE guidelines recommended for reporting animal research, and Sex and Gender in Research

Laboratory animals	Nematostella vectensis polyps derived from CH6 females and CH2 males from the original culture by Hand & Uhlinger 1992, as described in the Methods section.
Wild animals	n/a
Reporting on sex	The sex cannot be determined in juvenile polyps, and thus all data shown is from a mix of males and females.
Field-collected samples	n/a
Ethics oversight	No ethical approval is required for working with lab-reared sea anemones Nematostella vectensis for biological research.

Note that full information on the approval of the study protocol must also be provided in the manuscript.

Plants

Seed stocks	n/a
Novel plant genotypes	n/a
Authentication	n/a
